# Effects of the Coculture Initiation Method on the Production of Secondary Metabolites in Bioreactor Cocultures of *Penicillium rubens* and *Streptomyces rimosus*

**DOI:** 10.3390/molecules28166044

**Published:** 2023-08-13

**Authors:** Tomasz Boruta, Anna Ścigaczewska, Agnieszka Ruda, Marcin Bizukojć

**Affiliations:** Department of Bioprocess Engineering, Faculty of Process and Environmental Engineering, Lodz University of Technology, ul. Wolczanska 213, 93-005 Lodz, Poland

**Keywords:** coculture, secondary metabolites, *Penicillium rubens*, *Streptomyces rimosus*, stirred tank bioreactor

## Abstract

Bioreactor cocultures involving *Penicillium rubens* and *Streptomyces rimosus* were investigated with regard to secondary metabolite production, morphological development, dissolved oxygen levels, and carbon substrate utilization. The production profiles of 22 secondary metabolites were analyzed, including penicillin G and oxytetracycline. Three inoculation approaches were tested, i.e., the simultaneous inoculation of *P. rubens* with *S. rimosus* and the inoculation of *S. rimosus* delayed by 24 or 48 h relative to *P. rubens*. The delayed inoculation of *S. rimosus* into the *P. rubens* culture did not prevent the actinomycete from proliferating and displaying its biosynthetic repertoire. Although a period of prolonged adaptation was needed, *S. rimosus* exhibited growth and the production of secondary metabolites regardless of the chosen delay period (24 or 48 h). This promising method of coculture initiation resulted in increased levels of metabolites tentatively identified as rimocidin B, 2-methylthio-cis-zeatin, chrysogine, benzylpenicilloic acid, and preaustinoid D relative to the values recorded for the monocultures. This study demonstrates the usefulness of the delayed inoculation approach in uncovering the metabolic landscape of filamentous microorganisms and altering the levels of secondary metabolites.

## 1. Introduction

Microorganisms are a rich source of chemical substances of great industrial importance, including pharmaceutically relevant metabolites and enzymes [1,2]. The common approach to producing these valuable molecules is to employ monocultures (axenic cultures) of the selected production strains. However, the conditions of monocultivation greatly differ from those normally observed in the natural environment, where interactions among microorganisms are inevitable. The metabolic characteristics of microorganisms evolve in the context of their surrounding communities [3]. Many microbial species establish symbiotic relationships with other organisms that support mutual development or provide protection against harmful factors [4,5]. Other species compete for nutrients or space, using secondary metabolites and other “chemical weapons” to inhibit the growth of their competitors. Notably, every microbial species has a repertoire of secondary metabolites that are important for survival, e.g., metal-transporting agents or protective pigments [6]. To date, knowledge regarding the induction of secondary metabolic pathways for industrial applications is still limited. Microorganisms can be stimulated to produce secondary metabolites by manipulating the growth conditions or media composition [7]. A novel strategy is to grow different microbial species together in a single vessel to promote either cooperative or competitive interactions. Such an approach, referred to as coculture, is an effective unconventional method that unlocks the biosynthetic potential of microorganisms [8,9]. The investigation of cocultures opens the door for the discovery of novel metabolic pathways, an improved understanding of the interactions between microorganisms, and the development of methods for the increased production of secondary metabolites. Most importantly, this approach allows for expanding the diversity of chemical compounds produced by microorganisms [10]. Cocultures may involve species of the same (e.g., bacterium vs. bacterium) or different (e.g., fungus vs. bacterium) kingdoms [11]. Depending on the goals, microbial cocultivation can be carried out on laboratory plates, in glass flasks, or by using large-scale stirred tank bioreactors. Fundamental experiments regarding the influence of cocultivation on fungal secondary metabolism were performed using Petri dishes [12], and today, the plate-based approach is still widely used for the investigation of microbial interactions. Regarding submerged conditions, flat-bottom flasks and Erlenmeyer flasks are among the most frequently used coculture vessels. One example of a flask-based cocultivation study is the investigation of the effects exerted by the bacterium *Mycobacterium smegmatis* on the fungus *Aspergillus niger*. An important observation made in the study concerned the enhancement of the cytotoxic activity of the cultivation broth extract on prostate cancer cells [13]. An example of bioreactor cocultivation was reported by Luti and Mavituna [14], who focused on the improvement of undecylprodigiosin production by *Streptomyces coelicolor* in response to *E. coli*.

Fungal and bacterial cocultivation has become a powerful tool to enhance microbial secondary metabolism. *Penicillium rubens* is one of the most common household molds and is known to proliferate vigorously under high-humidity conditions. It is also recognized for producing β-lactam antibiotics, especially penicillin G [15]. This microorganism served as a coculture partner of *Aspergillus terreus* in shake flask experiments performed by Boruta et al. [16,17]. It was observed that *P. rubens* showed a tendency to be outperformed by *A. terreus* unless given an advantage in the form of delayed inoculation or modified ratios of the preculture volume. However, to the best of our knowledge, *P. rubens* has never been studied in the context of stirred tank bioreactor cocultivation. In the present work, *P. rubens* was cocultivated with the actinomycete, *S. rimosus*, a filamentous bacterium that exhibits a broad spectrum of secondary metabolites. *S. rimosus* is mostly recognized for its ability to produce a widely used antibiotic known as oxytetracycline [18]. In the previous work of Boruta and Ścigaczewska [19], *S. rimosus* was confronted with several filamentous microorganisms in shake flask cultures. The pairing of *S. rimosus* and *Streptomyces noursei* resulted in enhanced oxytetracycline production. Moreover, the authors investigated the influence of the coculture initiation time on oxytetracycline formation. They delayed the introduction of *S. rimosus* into the growth medium relative to *S. noursei* inoculation, which resulted in the lack of *S. rimosus* domination and inhibited biosynthesis of its secondary metabolites. A similar approach was applied by Boruta et al. [20], where *S. rimosus* and *A. terreus* were cocultured in stirred tank bioreactors. Two out of nine experiments were carried out with shifted *S. rimosus* inoculation times, which led to drastic changes in terms of the cocultivation outcomes (i.e., the domination of *S. rimosus* over *A. terreus* was nonexistent in these two experiments). To date, the interactions between the actinomycete, *S. rimosus*, and the fungus, *P. rubens*, in bioreactor cocultures have not been addressed in the literature.

The aim of this work was to study the influence of the coculture initiation method on the secondary metabolism of *Penicillium rubens* ATCC 28089 and *Streptomyces rimosus* ATCC 10970. The experiments were performed in stirred tank bioreactors, where each coculture was investigated simultaneously with the corresponding monocultures of *P. rubens* and *S. rimosus*.

## 2. Results

### 2.1. Secondary Metabolic Repertoire in Mono- and Cocultures

The experiments led to the detection of a broad biosynthetic landscape of 22 secondary metabolites (Table 1).

The identities of oxytetracycline and penicillin G were confirmed with the use of analytical standards. The remaining metabolites were tentatively identified on the basis of mass spectrometry data. However, their identities were not confirmed due to a lack of standards. Hence, with the exception of oxytetracycline and penicillin G, the secondary metabolites discussed here should be regarded as “putative” or “tentatively identified”. Among the detected products, sixteen molecules were assigned to the metabolic spectrum of *S. rimosus*, whereas the remaining six were suggested to be the products of *P. rubens*. In addition to oxytetracycline, the group of *S. rimosus* metabolites encompassed the molecules previously identified in the “*A. terreus* vs. *S. rimosus*” cocultivation study [20], including the metabolites representing the rimocidin family, i.e., rimocidin, CE-108, and rimocidin (27-ethyl), the products of the milbemycin family, i.e., milbemycin A_3_, milbemycin A_3_ + [4O], and milbemycin β_11_ + [4O]. Furthermore, the analysis revealed the presence of rimosamide A, which was previously reported for *S. rimosus* [28]. To the best of our knowledge, the remaining metabolites identified here have not yet been detected in *S. rimosus* but have been found in members of the *Streptomyces* genus, including rimocidin B, turgichelin, spinoxazine A, lucensomycin, 7-demethyl-glucopiericidin A, and the metabolite referred to as the “unnamed angucycline metabolite previously isolated from *Streptomyces* sp. QL37” [31]. The exception was 2-methylthio-cis-zeatin, a metabolite not yet found in *Streptomyces* but identified in *Actinomycetia*, the class that includes the *Streptomyces* genus. As far as the products of *P. rubens* are concerned, the analysis revealed the presence of penicillin G, chrysogine, benzylpenicilloic acid, and metabolites that, to the best of our knowledge, were not previously found in *P. rubens* but have been identified in other fungi representing the *Penicillium* genus, namely, adenophostin B, cyclopiamide D, and preaustinoid D. For comparative purposes, the total ion chromatograms (TICs) illustrating the evolution of chemical profiles over the course of PRSR1, PRSR2, and PRSR3 cultivations were analyzed. In the PRSR1 run (Figures S4–S11), the TICs corresponding to the coculture and *S. rimosus* monoculture overlapped throughout the duration of the process. In the PRSR2 (Figures S12–S20) and PRSR3 (Figures S21–S30) experiments, the overlap of TICs was observed only in the initial phase of cocultivation. More specifically, the last time the TICs overlapped was at the moment of *S. rimosus* inoculation, i.e., at t = 24 h and t = 48 h in the PRSR2 and PRSR3 runs, respectively. In addition to the metabolites presented in Table 1, the traces of the product with *m/z* value of longicatenamycin B were also detected (with Δ*m/z* = +0.0019 for the [M + H]^+^ ion) in the samples from the PRSR1 coculture and the PRSR1 monoculture of *S. rimosus*. This metabolite was recently characterized by Li et al. [40] as the metabolite of *S. rimosus*.

### 2.2. Production of Secondary Metabolites by S. rimosus

**Figure 1:**
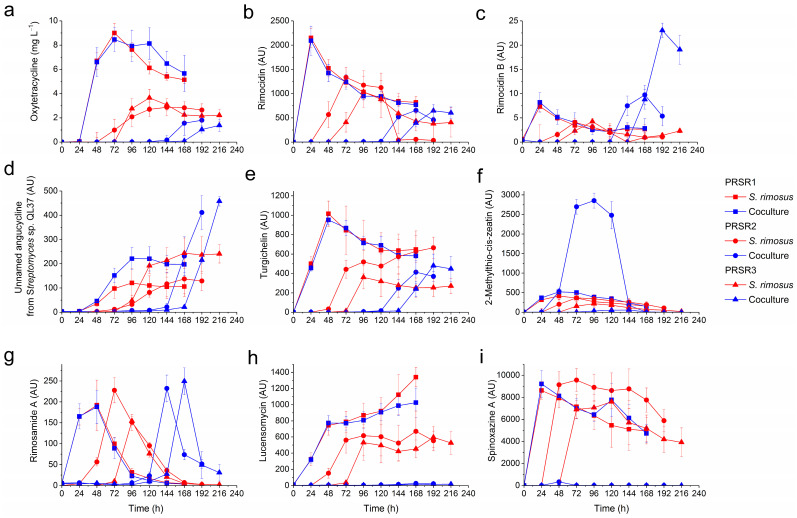
Production profiles of secondary metabolites biosynthesized by *S. rimosus* in mono- and cocultures over the course of the PRSR1, PRSR2, and PRSR3 experiments, namely, oxytetracycline (**a**) and the metabolites tentatively identified as rimocidin (**b**), rimocidin B (**c**), unnamed angucycline from *Streptomyces* sp. QL37 (**d**), turgichelin (**e**), 2-methylthio-cis-zeatin (**f**), rimosamide A (**g**), lucensomycin (**h**), and spinoxazine A (**i**). The mean values from three replicates are shown with error bars representing ± standard deviation. AU-auxiliary units.

In the PRSR1 coculture, which was initiated with the simultaneous inoculation of *S. rimosus* and *P. rubens*, the production profiles of *S. rimosus* metabolites (Figure 1) in most cases closely resembled those recorded for the corresponding *S. rimosus* monocultures. For example, the PRSR1 curves representing the coculture and monoculture levels of rimocidin (Figure 1b), rimocidin B (Figure 1c), turgichelin (Figure 1e), and rimosamide A (Figure 1g) practically overlapped. However, there were two secondary metabolites for which the PRSR1 coculture levels exceeded those observed for the monoculture variant, namely, 2-acetyl-2-decarboxamido-oxytetracycline (Figure S37) and the “unnamed angucycline metabolite previously isolated from *Streptomyces* sp. QL37” (Figure 1d).

In the PRSR2 and PRSR3 cocultures, *P. rubens* was introduced into the bioreactor 24 and 48 h prior to *S. rimosus* inoculation, respectively. In these experiments, one could observe three distinct outcomes in terms of the secondary metabolite production by *S. rimosus*, namely, the blocked biosynthesis of a given metabolite, biosynthesis that was observable but led to lower product levels than in the corresponding *S. rimosus* monoculture, or biosynthesis that resulted in higher metabolite levels than those observed in the monoculture. The first of these outcomes were recorded for spinoxazine A (Figure 1i), lucensomycin (Figure 1h), and 7-demethyl-glucopiericidin A (Figure S36), which were practically not produced in the PRSR2 and PRSR3 cocultures despite being found in the PRSR2 and PRSR3 monocultures. The second scenario was noted for oxytetracycline (Figure 1a), rimocidin (Figure 1b), CE-108 (Figure S31), 27-ethyl rimocidin (Figure S32), milbemycins (Figures S33–S35), and 2-acetyl-2-decarboxamido-oxytetracycline (Figure S37). The maximum levels of these metabolites in the PRSR2 and PRSR3 cocultures did not exceed those reached in the corresponding monocultures. Furthermore, the start of the production was visibly delayed compared to the monocultures, and the time of delay depended on the molecule. For example, the biosynthesis of oxytetracycline (Figure 1a) was confirmed after 48 h of *S. rimosus* growth in the PRSR2 and PRSR3 monocultures, whereas in the PRSR2 and PRSR3 cocultures, this metabolite was first detected as late as 120 h after *S. rimosus* inoculation, when trace amounts of oxytetracycline were found in the broth. In the case of rimocidin (Figure 1b), the onset of production in PSRS2 and PRSR3 cocultures was delayed by 96 h relative to their monoculture counterparts. Finally, in the third recorded scenario, the levels of several metabolites were found to be higher in the cocultures than in their monoculture counterparts. In this group, the stimulating effect of cocultivation could be observed either in one of the runs (either in PRSR2 or PRSR3) or in both, depending on the metabolite. The biosynthesis of rimocidin B (Figure 1c), which was the only member of the rimocidin family that displayed the cocultivation-related improvement in titer, was found to be visibly stimulated in the PRSR2 and PRSR3 cocultures, with the PRSR3 coculture being the most effective variant in this context. Similar stimulatory effects were noted for the biosynthesis of the “unnamed angucycline metabolite previously isolated from *Streptomyces* sp. QL37” (Figure 1d). In addition, the PRSR3 coculture led to a considerable increase in rimosamide A (Figure 1g) and turgichelin (Figure 1e) levels compared to the monocultures, while the PRSR2 coculture led to the stimulated formation of 2-methylthio-cis-zeatin (Figure 1f). With regard to the “cocultivation-enhanced” group of metabolites, it was observed that the time to reach the maximum titers in the PRSR2 and PRSR3 cocultures was visibly delayed compared to that in the monocultures (Figure 1b–e). The exception was 2-methylthio-cis-zeatin (Figure 1f), which was detected at 24 h after *S. rimosus* inoculation regardless of the cultivation mode (i.e., mono- or cocultivation).

### 2.3. Production of Secondary Metabolites by P. rubens

**Figure 2:**
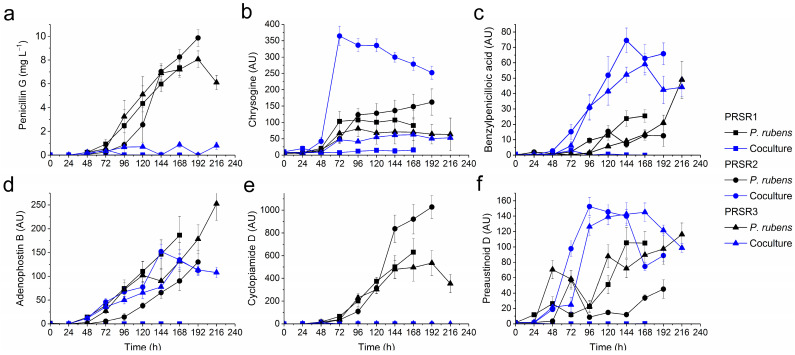
Production profiles of secondary metabolites biosynthesized by *P. rubens* in mono- and cocultures over the course of the PRSR1, PRSR2, and PRSR3 experiments, namely, penicillin G (**a**) and the metabolites tentatively identified as chrysogine (**b**), benzylpenicilloic acid (**c**), adenophostin B (**d**), cyclopiamide D (**e**), and preaustinoid D (**f**). The mean values from three replicates are shown with error bars representing ±standard deviation. AU-auxiliary units.

The production of *P. rubens* secondary metabolites in the PRSR1 coculture was found to be completely blocked, as opposed to the biosynthetic repertoire displayed in the PRSR1 fungal monoculture (Figure 2). In the PRSR2 and PRSR3 cocultures, on the other hand, the outcome of the mono- vs. coculture comparison varied from metabolite to metabolite.

Regarding the production of penicillin G (Figure 2a), cocultivation clearly exerted negative effects on the biosynthetic process. Among the tested cocultures, only PRSR3 resulted in detectable levels, although the corresponding titers were very low, i.e., not exceeding 1 mg L^−1^ (Figure 2a). An even stronger inhibitory effect was noted with regard to cyclopiamide D production (Figure 2e), which was completely turned off as a result of cocultivation. In contrast, there was an evident boost in benzylpenicilloic acid (Figure 2c) and preaustinoid D (Figure 2f) levels in the PRSR2 and PRSR3 cocultures compared to the corresponding *P. rubens* monocultures. Furthermore, the stimulation of adenophostin B (Figure 2d) and chrosogine (Figure 2b) biosynthesis was recorded for the PRSR2 cocultivation. The levels of *P. rubens* metabolites in the PRSR2 and PRSR3 cocultures increased only until a certain time point (e.g., 144 or 168 h in the case of adenophostin B production in the PRSR2 and PRSR3 experiments, respectively, as shown in Figure 2d) and then declined toward the end of the cocultivation process.

### 2.4. Dissolved Oxygen Profiles

**Figure 3:**
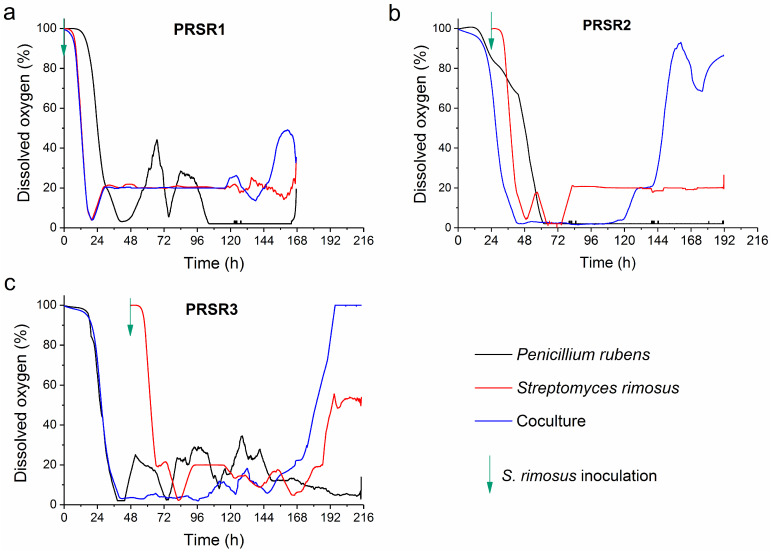
Dissolved oxygen (DO) levels in the monocultures and cocultures of *S. rimosus* and *P. rubens* over the course of the PRSR1 (**a**), PRSR2 (**b**), and PRSR3 (**c**) experiments. The time of *S. rimosus* inoculation is depicted by an arrow.

In the PRSR1 experiment, in which the coculture was simultaneously inoculated with the spores of both species (at time t = 0 h, Figure 3a), it was evident that during the whole duration of the process (i.e., except the period from 144 to 168 h), the DO curve of the coculture overlapped with that observed for the *S. rimosus* monoculture. In other words, from 0 to 144 h of the run, the DO profile of the PRSR1 coculture behaved as if *P. rubens* was absent from the broth (Figure 3a). The situation was markedly different in the PRSR2 process (Figure 3b), where the spores of *S. rimosus* were introduced at t = 24 h into the bioreactor with growing *P. rubens* to initiate the cocultivation. At the same moment (i.e., at 24 h), the monoculture of *S. rimosus* was started (Figure 3b). 

The first observation was that the PRSR2 monoculture of *P. rubens* required as much as 60 h to display a DO level of 0%, whereas the corresponding coculture needed only 42 h to reach this state (Figure 3b). Thus, it was evident that *S. rimosus* contributed to oxygen utilization in the coculture. Another observation was made regarding the DO profiles after they dropped to the 0% level. In the case of the *P. rubens* monoculture, the DO curve remained at the 0% plateau, while in the *S. rimosus* monoculture and the coculture, an abrupt increase in DO occurred (Figure 3b), albeit at different time points (at t = 72 h and t = 120 h for the *S. rimosus* monoculture and coculture, respectively). A unique factor in the PRSR2 coculture profile compared with the corresponding *S. rimosus* and *P. rubens* monoculture variants is the behavior exhibited after 140 h of the run (Figure 3b). During this final period of the PRSR2 run, the DO in the coculture showed continuous changes and temporarily reached levels as high as 90%, whereas, in the *P. rubens* and *S. rimosus* monocultures, the DO curves remained at 0 and 20%, respectively, for the remainder of the experiment (Figure 3b). In the third investigated bioprocess, namely, PRSR3, the coculture was initiated by inoculating the *S. rimosus* spores into the 48-h culture of *P. rubens* (Figure 3c). Within almost the whole period between 48 and 150 h, the DO levels in the *P. rubens* monoculture were higher than those in the coculture. Then, behavior similar to what was seen in the PRSR2 coculture was recorded, i.e., the DO levels increased abruptly in the coculture and *S. rimosus* monoculture, while the DO levels in the *P. rubens* monoculture showed an opposite trend and kept decreasing toward the end of the run (Figure 3c). In the period between 172 and 216 h, the DO profile in the *P. rubens* monoculture was evidently below the curves corresponding to the coculture and *S. rimosus* monoculture. It should also be mentioned that, unlike the PRSR2 counterpart, the DO profile in the PRSR3 coculture reached 100% and remained at this level until the end of the experiment (Figure 3c).

### 2.5. Kinetics of Substrate Utilization

**Figure 4:**
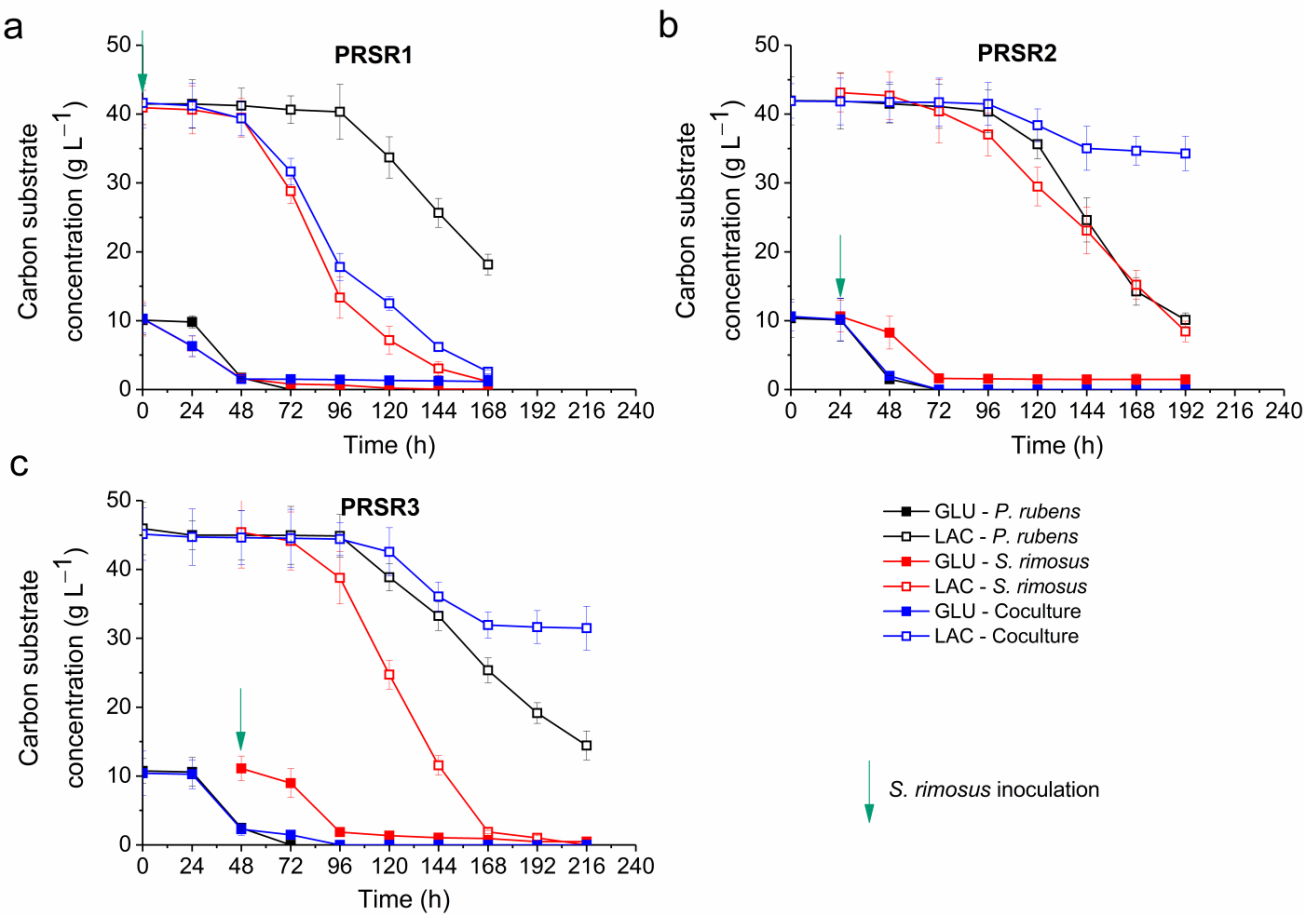
Levels of carbon substrates (glucose and lactose) in the monocultures and cocultures of *S. rimosus* and *P. rubens* over the course of the PRSR1 (**a**), PRSR2 (**b**), and PRSR3 (**c**) experiments. The time of *S. rimosus* inoculation is depicted by an arrow. The mean values from three replicates are shown with error bars representing ± standard deviation. GLU—glucose; LAC—lactose.

In the PRSR1 experiment, the glucose and lactose concentration profiles of the coculture resembled those recorded for the *S. rimosus* monoculture; however, they were not identical, i.e., the sugar concentration values in the coculture were slightly higher than those in the *S. rimosus* monoculture (Figure 4a). The monoculture of *P. rubens* started to assimilate glucose and lactose relatively late compared to the two other variants. It was evident that the utilization of lactose by *P. rubens* was not at comparable levels to that observed in the coculture and *S. rimosus* monoculture (Figure 4a). 

The situation looked markedly different in the PRSR2 run (Figure 4b), in which the spores *S. rimosus* were introduced with a 24-h delay. Here, the glucose concentration profiles of the *P. rubens* monoculture, and the coculture overlapped throughout the cultivation despite the addition of *S. rimosus* spores (Figure 4b). In the case of lactose, the concentration profile in the coculture followed that in the *P. rubens* monoculture but only until 96 h. Afterward, the lactose concentration curves in the *P. rubens* monoculture, and coculture fell apart, and lactose was hardly utilized in the coculture after 144 h of the run, remaining at the level of approximately 37 g L^−1^. In the *P. rubens* monoculture, the lactose concentration decreased by more than 30 g L^−1^ from 96 to 192 h (Figure 4b). A similar decrease in lactose levels was noted for the *S. rimosus* monoculture (Figure 4b). It should be mentioned that in the PRSR2 run, the lactose profiles corresponding to the coculture and *S. rimosus* monoculture differed markedly (Figure 4b). In the PRSR3 experiment, in which *S. rimosus* was inoculated with a 48-h delay (Figure 4c), the glucose concentration profiles of the coculture and *P. rubens* monoculture were practically identical only until 48 h. Then, the level of glucose dropped to zero at 72 h in the *P. rubens* monoculture, while the coculture required 96 h to completely utilize this sugar (Figure 4c). The lactose profiles for the coculture and *P. rubens* monoculture overlapped until 96 h. Then, the levels of lactose recorded in the coculture were clearly above those observed in the monoculture of *P. rubens*. In the coculture, the lactose concentration remained at 33 g L^−1^ from 168 to 261 h, while in the *P. rubens* monoculture, it decreased to approximately 15 g L^−1^ at 216 h (Figure 4c). At the same time, the consumption of lactose in the *S. rimosus* monoculture was greater than those in the other two variants and dropped to zero at 216 h (Figure 4c).

**Figure 5:**
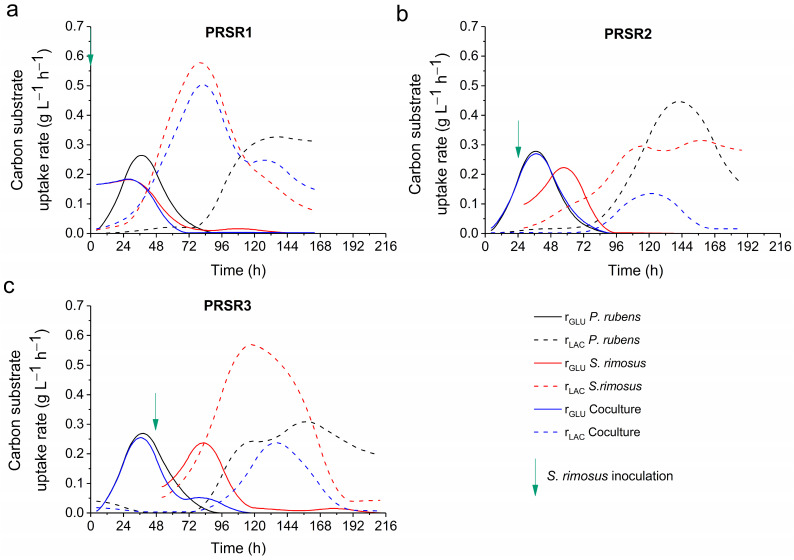
Volumetric uptake rates of glucose r_GLU_ and lactose r_LAC_ in the monocultures and cocultures of *S. rimosus* and *P. rubens* over the course of the PRSR1 (**a**), PRSR2 (**b**), and PRSR3 (**c**) experiments. The time of *S. rimosus* inoculation is depicted by an arrow. GLU—glucose; LAC—lactose.

Analysis of the glucose and lactose uptake rates (Figure 5) confirmed the aforementioned observations. In the PRSR1 experiment, the monoculture of *P. rubens* exhibited a unique pattern of uptake rates compared with the coculture and *S. rimosus* monoculture. At the same time, the profiles of the coculture and *S. rimosus* monoculture were highly similar, albeit not identical (Figure 5a). The maximum lactose uptake rate r_LAC_ for the *P. rubens* monoculture was equal to 0.27 g LAC L^−1^ h^−1^ at t = 36 h, while those for the *S. rimosus* monoculture and coculture exceeded 0.5 g LAC L^−1^ h^−1^ at approximately 72 h of the PRSR1 process (Figure 5a). 

The PRSR2 and PRSR3 experiments were similar to each other with regard to the glucose uptake rates r_GLU_ in the period between 0 and 72 h, with the maximum values reaching almost 0.3 g GLU L^−1^ h^−1^ at 36 h (Figure 5b,c). Regarding the lactose uptake rates r_LAC_, their values in the coculture were lower than those recorded in the *S. rimosus* and *P. rubens* monocultures (Figure 5b,c).

### 2.6. Morphological Analysis

**Figure 6:**
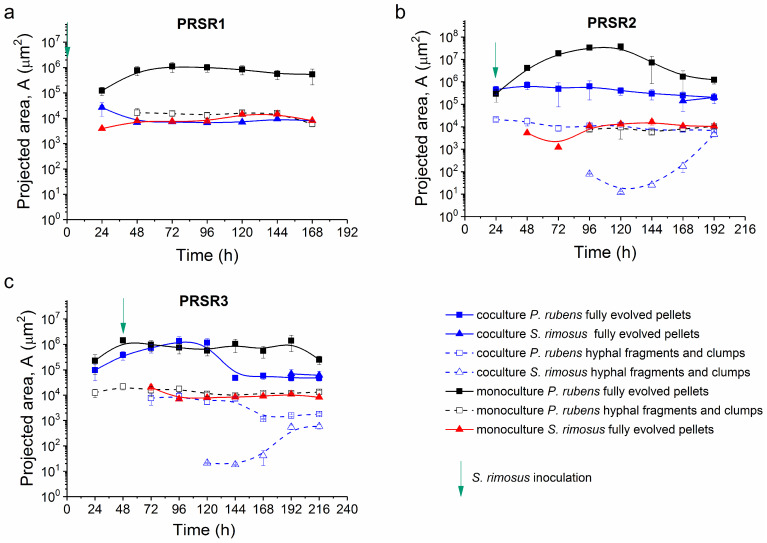
Projected area (A) of fully evolved pellets and hyphal fragments and clumps in the monocultures and cocultures of *S. rimosus* and *P. rubens* over the course of the PRSR1 (**a**), PRSR2 (**b**), and PRSR3 (**c**) experiments. The time of *S. rimosus* inoculation is depicted by an arrow. The mean values calculated for at least 30 morphological objects and the confidence bands (significance level α = 0.05) are depicted.

The morphological analysis (Figure 6, Figures S38 and S39) involved three parameters [41] calculated on the basis of microscopic images (Figure S40): projected area (A), which refers to the object size (number of pixels in an object multiplied by squared unit of calibration); elongation (E), which describes the shape (squared quotient of transversal and longitudinal deviation of pixels in an object along the line of regression); and morphology number (Mo) introduced by Wucherpfennig et al. [42], which combines the influence of size and shape within a single parameter value (it is proportional to the square root of projected area and roughness, and inversely proportional to elongation and maximum object diameter). 

In the case of filamentous microorganisms such as *P. rubens* and *S. rimosus*, their primary morphological forms are always developed during the first 24–48 h of their growth. This process was clearly seen in experiment PRSR1. The projected area values of the spores introduced to the bioreactors at t = 0 h were equal to 8 µm^2^ for *P. rubens* and 0.38 µm^2^ for *S. rimosus*. During the first 24 h of PRSR1, the microorganisms grew rapidly and formed pellets, i.e., spherical morphological forms with areas of 1.2 × 10^5^ µm^2^ for *P. rubens* in the monoculture and 3.9 × 10^3^ µm^2^ for *S. rimosus* in the monoculture (Figure 6a). *P. rubens* pellets in the PRSR1 coculture were present only at 24 h of the process. Within the next hours of cultivation, they vanished completely due to the presence of *S. rimosus* in the coculture (Figure 6a and Figure S40). At 48 h of the PRSR1 experiment, *S. rimosus* pellets in the monoculture and the coculture became more homogeneous. The projected area of the *S. rimosus* monoculture increased to 7.8 × 10^3^ µm^2^, and that of the coculture decreased to 6.8 × 10^3^ µm^2^ (Figure 6a). The *p*-values lower than 0.01 calculated for these two projected area values indicate significant differences. However, in the next hours of cultivation, the *S. rimosus* pellet sizes in the monoculture and coculture were almost identical. Their projected area values were 7.5 × 10^3^ µm^2^ (monoculture) and 7.2 × 10^3^ µm^2^ (coculture). A *p*-value higher than 0.05 confirmed the lack of significant differences. Similar sizes of *S. rimosus* objects in the mono- and cocultures at this time suggest that the presence of *P. rubens* in the coculture had no significant effect on *S. rimosus* growth. Thus, *P. rubens* pellets did not survive the PRSR1 cocultivation, whereas, in the monoculture, they were present until the end of the experiment. The other morphological forms witnessed in PRSR1, namely, hyphae and clumps, were exclusively formed in the *P. rubens* monoculture (Figure 6a).

In the PRSR2 experiment, fungal and actinomycete pellets appeared after 24 h of growth. At this time of the process, hyphae and clumps were present only in the *P. rubens* monoculture (Figure 6b). In the PRSR2 coculture, the pellets of *P. rubens* were observed until the end of the experiment. Their sizes in the cocultivation bioreactor before *S. rimosus* inoculation were similar to those from the *P. rubens* monoculture. At 24 h of PRSR2, the area of *P. rubens* pellets were 4.5 × 10^5^ µm^2^ in the coculture and 2.9 × 10^5^ µm^2^ in the monoculture (*p* > 0.05). Then, the size of the *P. rubens* pellets in the coculture showed a decreasing trend (Figure 6b). Hyphae and clumps of *S. rimosus* were formed at 96 h of the PRSR2 coculture, while the pellets appeared at 168 h. At 192 h, the mean projected area of *S. rimosus* pellets was the same as the value recorded for the pellets of *P. rubens* (Figure 6b).

Compared with PRSR2, the pellets of *P. rubens* in the PRSR3 run showed a prolonged period of growth, as illustrated by the values of the projected area (Figure 6c). The peak value of the projected area (A = 1.3 × 10^6^ µm^2^) of *P. rubens* pellets was reached at 96 h in the PRSR3 process. Within the next hours of cocultivation, the projected area of *P. rubens* exhibited a decreasing trend (at 144 h, a projected area of 4.8 × 10^4^ µm^2^ was recorded). Importantly, the decrease in the *P. rubens* pellet size in the PRSR3 coculture was accompanied by the visible emergence of *S. rimosus* hyphal fragments and clumps (Figure 6c).

The shapes of the pellets observed during the experiments were irregular, as indicated by the morphology number at the level of Mo = 0.4 or even lower (Figure S38). The value of Mo = 0.9, which corresponds to the circular objects, was recorded only in the PRSR2 monoculture of *P. rubens*. Moreover, the morphology number obtained for hyphal fragments and clumps (within the range of 0.1–0.35) was always lower than the corresponding value for pellets, which confirms the correct separation of morphological objects into these two fractions. The values of elongation presented in Figure S39 were also in agreement with the theoretical considerations, i.e., the less elongated pellets displayed elongation values not higher than E = 2, whereas hyphae and clumps reached levels as high as E = 3.6 (see the *P. rubens* monoculture in the PRSR2 run at 120 h, Figure S39).

## 3. Discussion

In a previous study focused on the bioreactor cocultivation of filamentous microorganisms [20], it was demonstrated that the simultaneous inoculation of *S. rimosus* with the filamentous fungus *A. terreus* led to a state characterized by the inhibited growth of *A. terreus* and blocked production of fungal secondary metabolites. Giving *A. terreus* a 24-h growth advantage led to the opposite outcome, i.e., the fungus prevented *S. rimosus* from developing and producing its metabolites. In the present work, initiating the coculture by the simultaneous inoculation of *S. rimosus* and *P. rubens* resulted in *P. rubens* being outperformed *by S. rimosus*. Considering the results obtained for the cocultivation of *A. terreus* and *S. rimosus* [20], it was expected that providing an additional 24-h growth period for *P. rubens* would lead to a reversed outcome, i.e., the actinomycete being outperformed by the fungus. However, these predictions turned out to be incorrect. Although the presence of *P. rubens* biomass caused a delay in *S. rimosus* biomass development in the PRSR2 coculture, the actinomycete exhibited visible growth and biosynthetic activity, as illustrated by the morphological events that were monitored microscopically, and the chemical profiles reflected by the TICs. The propagation of *S. rimosus* was “awakened” in the mature stage of the PRSR2 coculture, and this event was accompanied by the triggered production of secondary metabolites, e.g., oxytetracycline, rimocidin, rimocidin B, rimosamide A, and turgichelin. This is a clear indication that the performance of *P. rubens* against *S. rimosus* was not comparable with the outcomes of the “*A. terreus* vs. *S. rimosus*” coculture. While the already-developed filamentous biomass of *A. terreus* greatly restricted the proliferation of *S. rimosus* and disabled its expansion [20], *P. rubens* was found to be far less effective in this respect. At this point, the question remained as to what would happen if *P. rubens* had even more time to establish its monoculture and generate higher cell densities before being confronted by *S. rimosus*. In response to this question, the study was further continued to include the PRSR3 process, in which the actinomycete was inoculated with a longer (i.e., 48-h) delay. We are not aware of any previous reports on the submerged cocultivation of filamentous species that addressed the impact of different inoculation delay times. The PRSR3 experiment clearly demonstrated that even after the prolonged period of undisturbed growth under monoculture conditions, *P. rubens* was as vulnerable to the proliferation of *S. rimosus* as it was in the PRSR2 run. Nevertheless, in the context of secondary metabolite production, changing the *S. rimosus* inoculation delay period from 24 h (in PRSR2) to 48 h (in PRSR3) was not without consequences. Two notable effects were the evident increase and decrease of rimocidin B and chrysogine levels, respectively. In the case of chrysogine, the metabolite of *P. rubens* was intriguing since applying the 48-h delay prevented the stimulatory effects that were witnessed when the 24-h delay was used. In other words, even though *P. rubens* had more time to develop before confronting *S. rimosus* in the PRSR3 coculture than in the PRSR2 coculture; the biosynthesis of chrysogine in the former process was definitely less efficient than that in the latter process. This shows that a given inoculation scheme leads to a unique set of cocultivation outcomes, which may be counterintuitive at times and always require experimental verification.

The “*S. rimosus* vs. *P. rubens*” cocultures were subjected to a multilevel analysis. The results describing the production metabolites, the utilization of sugars, and DO correlate well with the microscopic images, and the morphological analysis, including the microscopic analysis in the study, allowed for obtaining a broader perspective on the events taking place in the coculture. It was clear that *S. rimosus* managed to develop its biomass despite being inoculated into a 24-h or 48-h culture of *P. rubens*. However, it did not mean that the growth of *S. rimosus* influenced the coculture to such an extent that the coculture followed the bioprocess kinetics exhibited by the *S. rimosus* monoculture. This issue is perfectly illustrated by the time courses of lactose utilization. As opposed to the corresponding monoculture variants, the PRSR2 and PRSR3 cocultures did not show lactose consumption during the final two days of the run. This was also the time when the DO levels of the PRSR2 and PRSR3 cocultures were evidently higher than the values recorded in the monocultures. This demonstrates that the PRSR2 and PRSR3 cocultures had unique kinetic characteristics after the growth of *S. rimosus* and the production of its secondary metabolites had become visible. It also showed that sugar and oxygen utilization in cocultures cannot be predicted based on the values recorded for the monocultures.

A common observation regarding the DO profiles in the PRSR1, PRSR2, and PRSR3 cocultures was that during the final day of the process, the highest oxygen levels were always seen in the coculture, followed by the results recorded for the monoculture of *S. rimosus* and, finally, by the visibly lower (i.e., close to zero) values exhibited by the *P. rubens* monoculture. It is possible that the relatively high DO concentration during the final 2 days of the PRSR2 and PRSR3 cocultures contributed to the exceptionally high levels of rimocidin B and the “unnamed angucycline metabolite previously isolated from *Streptomyces* sp. QL37”. Even in the PRSR1 cocultivation, which resembled the monocultivation of *S. rimosus* in terms of the exhibited secondary metabolic repertoire and growth morphology, the DO level in the period from 144 to 168 h was clearly higher than that in the corresponding *S. rimosus* monoculture. Considering the fact that the production of two secondary metabolites of *S. rimosus*, namely, 2-acetyl-2-decarboxamido-oxytetracycline and the “unnamed angucycline metabolite previously isolated from *Streptomyces* sp. QL37”, was enhanced in the PRSR1 coculture relative to the *S. rimosus* monoculture, it was clear that *P. rubens* exerted observable effects on *S. rimosus* during cocultivation despite being outperformed and greatly inhibited. This observation is in agreement with our previous studies on *S. rimosus*. Briefly, it was demonstrated that if *A. terreus* [20] or *S. noursei* [19] were inoculated simultaneously with *S. rimosus* to initiate submerged coculture, their development was greatly inhibited by *S. rimosus*. At the same time, the outperformed microorganisms, i.e., *A. terreus* and *S. noursei*, influenced the growth and biosynthetic performance of *S. rimosus* during cocultivation, e.g., by affecting its ability to produce secondary metabolites [19,20]. The molecular details of such interactions, however, remain to be elucidated.

The investigation of coculture systems requires the viability and detectable activity of all involved species. In the studies centered on the production of secondary metabolites, one challenging aspect of such efforts is the adjustment of the inoculation scheme and/or process conditions to avoid the situation in which one of the microbial species is practically eliminated, and its biosynthetic repertoire cannot be explored. In this context, the control of population dynamics in submerged cocultures is highly desired [43,44,45]. The common difficulty associated with designing a bioreactor coculture is achieving a system that supports the growth and secondary metabolite production of all cocultivated microorganisms. In the “*A. terreus* vs. *S. rimosus*” coculture study [20], one of the microorganisms was always outperformed by its partner, so the spectrum of secondary metabolites in each bioprocess was limited. In other words, the species that was given a 24-h time advantage, either *S. rimosus* or *A. terreus*, did not allow for the accompanying microbe to reveal its true potential. In a different study [46], the simultaneous inoculation of *A. terreus* with the nystatin-producing actinomycete *S. noursei* allowed for, quite by chance, achieving a coculture that displays the secondary metabolic production of both involved species. However, this was not observed in the present work on *P. rubens* and *S. rimosus*. Introducing the spores of these species into the bioreactor at t = 0 h resulted in a coculture that practically displayed no production of fungal secondary metabolites. What happened to be an effective strategy, in this case, was to grant a less “aggressive” species, i.e., *P. rubens*, 24 or 48 h before inoculating the more “aggressive” microorganisms into the bioreactor. Here, the term “aggressiveness” can be understood as the ability of a given species to inhibit the development of the accompanying microbe in the coculture. It is associated with the biomass-specific growth rate, as well as with the bioactivity and quantity of secreted metabolites and enzymes. In contrast to what happened in the “*A. terreus* vs. *S. rimosus*” study, *S. rimosus* was able to adapt to the pressures exerted by *P. rubens* and entered the phases of submerged development in the PRSR2 and PRSR3 cocultures. The first success of the delayed inoculation approach was that the PRSR2 and PRSR3 broths contained the metabolites of both cocultivated species. Moreover, the production of several molecules was stimulated in the coculture relative to the monoculture, e.g., rimocidin B, 2-methylthio-cis-zeatin, and chrysogine. It should also be mentioned that some metabolites were found in the monocultures but were absent from the PRSR2 and PRSR3 cocultures, e.g., lucensomycin, spinoxazine, and cyclopiamide D. From a more general perspective, the results of the study indicate that inoculation delay may, in principle, be an effective strategy to enhance the production of target metabolites and, at the same time, inhibit the formation of unwanted molecules in submerged cocultures. However, if the goal is to observe the biosynthetic spectra of both cocultivated species, it is a prerequisite to have a sufficient difference between the two microbes in terms of their relative “aggressiveness”. For example, *P. rubens* did not manage to outperform *S. rimosus* inoculated after 24 or 48 h, but *A. terreus* was found to be able to do so [20]. Hence, inoculation delay can be a very effective method of coculture initiation, provided the delayed species is able to survive in the coculture, as was observed in the current work. This requires experiments to determine whether a given microbial pair behaves like the “*A. terreus* vs. *S. rimosus*” system or rather resembles the “*P. rubens* vs. *S. rimosus*” case.

Although *S. rimosus* eventually gained momentum in terms of growth and metabolite production in the PRSR2 and PRSR3 cocultures, it did not reveal the complete catalog of its metabolites that is normally found in monocultures. Specifically, the biosynthesis of lucensomycin and spinoxazine remained blocked throughout the cocultivation. This behavior can be explained by considering the levels of nutrients available to *S. rimosus* during cocultivation. Microbial secondary metabolism is known to respond to even the slightest changes in environmental conditions, including the availability of substrates [47]. Since *P. rubens* had a time advantage over *S. rimosus* in the PRSR2 and PRSR3 coculture runs, the consumption of substrates by the fungal mycelia could exert effects on the production of secondary metabolites by *S. rimosus*, i.e., the shutdown of lucensomycin and spinoxazine biosynthesis. Another possibility is that the inhibition of the metabolic activity of *S. rimosus* could be due to the chemical and physical interactions between the cocultivated species, e.g., through bioactive metabolites or cell-to-cell contact that triggered an intracellular regulatory response [48]. It is worth mentioning that the production of rimocidin B was enhanced due to cocultivation, as opposed to the case of the biosynthetically related molecule rimocidin. This demonstrates the fact that the response of a given biosynthetic pathway to cocultivation is not necessarily uniform. Hence, the coculture can be used to deregulate the pathways and stimulate the formation of metabolites that are normally generated at low yields. An interesting observation was made with regard to the formation of 2-methylthio-cis-zeatin. Its production was strongly enhanced in the PRSR2 coculture. In addition, it was the only metabolite biosynthesized by *S. rimosus* in the early phase of PRSR2 cocultivation, when *P. rubens* was still a dominant species. The results indicate that 2-methylthio-cis-zeatin was efficiently produced when *S. rimosus* was developing from its spores but not in the late phases of cultivation. According to Čihák et al. [49], who studied the formation of secondary metabolites by *S. coelicolor* during the germination phase, such “early growth stage” products may play a role in coordinating developmental events and repressing the competitive microbiota in the environmental niche.

It is important to note that the moment when *S. rimosus* growth became visible in the PRSR2 and PRSR3 cocultures was reflected by the time profiles of metabolite levels, i.e., the secondary metabolites of *S. rimosus* were practically not recorded before this characteristic time point. In the PRSR2 coculture, this behavior was observed at approximately 144 h, when considerable amounts of oxytetracycline, rimocidin, rimocidin B, and turgichelin were detected in the broth. If the same set of metabolites is considered to define this characteristic moment in the PRSR3 experiment, the onset of production corresponded to a time of approximately 168 h. This was expected, as the inoculation time of PRSR2 and PRSR3 with *S. rimosus* differed by 24 h. One may argue that the production of rimosamide A in PRSR2 and PRSR3 was visible one day before the time points given above; however, this was an exceptional case. Notably, the moment when *S. rimosus* growth became visible corresponded with the time when the levels of benzylpenicilloic acid, adenophostin B, and preaustinoid D, i.e., the metabolites originating from *P. rubens*, started to decline. The final confirmation of the characteristic time points was provided by the DO profiles. At approximately 144 and 168 h, the DO profiles of the PRSR2 and PRSR3 cocultures, respectively, started to exhibit their unique character and moved away from the curves recorded in the monocultures, especially the *P. rubens* variant. Notably, assigning a characteristic time point to the moment when *S. rimosus* propagation became visible should be regarded as a simplification used to describe and understand the events taking place in the coculture. Furthermore, it should be seen as the culmination of an ongoing process rather than a sudden event. The presence of 2-methylthio-cis-zeatin early in the PRSR2 coculture indicates that *S. rimosus* germinated long before 144 h, i.e., soon after its inoculation at 24 h. Its viability was also manifested by its influence on the levels of *P. rubens* metabolites over the course of the PRSR2 and PRSR3 cocultures. On the other hand, the proliferation of *S. rimosus* at the early phase of coculture was not sufficient to support the detectable production of oxytetracycline, rimocidin, and the remaining secondary metabolites of the biosynthetic landscape of this actinomycete. 

For the PRSR2 coculture, the level of DO was close to zero from 48 to 120 h. Similar behavior was recorded for the PRSR3 coculture, where the DO was close to zero from 48 to 96 h. Then, the DO increased abruptly in PRSR2 and PRSR3 cocultures. This behavior was probably associated with the changing composition of microbial populations in the bioreactor. The period of very low DO levels corresponded to the time of intensive oxygen consumption by the biomass of *P. rubens*. When the development of *S. rimosus* biomass started to exert inhibitory effects on *P. rubens*, the DO levels in PRSR2 and PRSR3 cocultures started to increase as well. In other words, the consumption of oxygen by fungal biomass was weakened due to the increasing activity of *S. rimosus* in the coculture. At the same time, the development of *S. rimosus* in the late phase of PRSR2 and PRSR3 cocultures was not associated with intensive oxygen consumption since the DO levels gradually increased towards the end of the cocultivation. It should be pointed out that the conditions were not favorable for *S. rimosus* to proliferate, i.e., the fungal biomass and secondary metabolites were present in the bioreactor, and the levels of nutrients were not as high as in the fresh medium. It could be the reason why the development of *S. rimosus* in the late phase of PRSR2 and PRSR3 cocultures was accompanied by the increase in the DO levels. Finally, it should be noted that the current efforts were focused on monitoring the measurable effects of cocultivation, with a focus on secondary metabolite production, but the molecular details of microbial interactions and the mathematical consideration of population dynamics were beyond the scope of the study and need to be addressed in future experimental work.

The analysis of morphological parameters provided a quantitative description of the developmental events taking place in the cocultures. The comparison of projected area values was highly illustrative with regard to the fate of *P. rubens* pellets in the PRSR1, PRSR2, and PRSR3 runs. In the PRSR1 coculture, fungal pellets were noticed solely at 24 h of the process. After this time, the presence of *P. rubens* pellets was not recorded in the PRSR1 coculture. In the case of PRSR2, the emergence of *S. rimosus* biomass (hyphal fragments, clumps, and pellets) reflected the visible development of *S. rimosus* in the coculture. The pellets of *P. rubens* were detected throughout the PRSR2 coculture run; however, their projected area values were (with the exception of the data gathered at 24 h) clearly below those observed in the *P. rubens* monoculture. In contrast, the pellets of *P. rubens* that evolved in the PRSR3 coculture were comparable with the *P. rubens* monoculture values in terms of the projected area until 120 h of the run. Then, the coculture curve of the projected area values was clearly situated below the corresponding *P. rubens* monoculture curve. The time of *S. rimosus* inoculation delay affected the size of *P. rubens* pellets relative to the monoculture. In a more general context, this demonstrates that the change in secondary metabolite production due to the inoculation delay was accompanied by measurable morphological changes. To the best of our knowledge, this study was the second morphological analysis on filamentous cocultures in bioreactors. Previously, Ścigaczewska et al. [50] presented a similar characterization with regard to the “*A. terreus* vs. *S. rimosus*” cocultivation. Both studies demonstrate that the results of the quantitative morphological analysis are in agreement with the events related to the production of secondary metabolites and can be used to monitor and comparatively analyze the course of cocultivations in stirred tank bioreactors.

Performing microbial cocultivations aimed at secondary metabolite production is not a trivial task. This approach is associated with many bioprocess-related challenges and difficulties [45,51,52,53,54,55,56,57]. Most importantly, the differences in terms of growth rate often lead to a scenario in which a faster-growing species outcompetes its microbial partner, and, as a result, the metabolites of only one microorganism are produced in the system [51]. Inoculating the slower-growing species before a faster-growing species, as was done in the present study, is a way to prevent the elimination of the slower-growing microorganism in the coculture. The microbial population dynamics during cocultivation can also be influenced by the changes in medium osmolality, agitation conditions, or inoculum ratios [45]. Another important aspect of cocultivation is related to time. It was shown in the previously published studies [51,52,53,54] and in the present work as well that the levels of secondary metabolites depend on the time of growth of the cocultivated species. Analyzing time-series data is thus crucial to monitor the productivity of cocultures in terms of secondary metabolite levels. In the current work, it was exemplified by the fact that in the PRSR2 and PRSR3 experiments, the products of *S. rimosus* were typically found in the late phase of cocultivation. In a different study on fungal secondary metabolism, Bertrand et al. [51] reported that even though the levels of most investigated products increased over the course of the coculture, some of the compounds were detected solely at certain time points in time. In another work, Azzollini et al. [54] analyzed the dynamics of volatile and non-volatile secondary metabolite induction in fungal cocultures based on time-series data and described the characteristic induction patterns exhibited in fungal-fungal cocultivation systems. In metabolomic studies, the dynamics of metabolite production in the cocultures can be investigated with the aid of chemometric tools and computational approaches, such as ANOVA-PCA (Analysis of Variance—Principal Component Analysis) combined with POCHEMON (Projected Ortogonalized CHemical Encounter MONitoring) [53].

To conclude, the simultaneous inoculation of growth medium with the spores of *S. rimosus* and *P. rubens* results in a coculture dominated by the former species, i.e., the production of fungal secondary metabolites is practically nonexistent. In contrast, delaying the *S. rimosus* inoculation by 24 or 48 h relative to the inoculation of *P. rubens* leads to the active biosynthesis of secondary metabolites of both cocultivated species. This approach greatly affects the biosynthetic outcomes of the coculture, as illustrated by the increased levels of the metabolites tentatively identified as rimocidin B, 2-methylthio-cis-zeatin, chrysogine, benzylpenicilloic acid and preaustinoid D relative to the values recorded for the monocultures. The delayed inoculation of *S. rimosus* into the *P. rubens* culture did not prevent *S. rimosus* from developing and displaying its biosynthetic repertoire. The viability of *S. rimosus*, in this case, is reflected by the metabolic repertoire, morphological characteristics, and bioprocess kinetic data collected during the cocultivation. The results demonstrate that the delayed inoculation method is a highly promising approach for coculture initiation; however, its effectiveness is greatly dependent on the level of mutual “aggressiveness” displayed by the cocultured microorganisms.

## 4. Materials and Methods

### 4.1. Strains

*Penicillium rubens* ATCC 28089, and *Streptomyces rimosus* ATCC 10970 strains were purchased from the American Type Culture Collection (ATCC) and used throughout the study. *P. rubens* was stored on slants made from potato broth (300 g of potatoes boiled in 500 mL of water), glucose (20 g L^−1^), and agar (20 g L^−1^). *S. rimosus* was stored on agar slants according to the ATCC instructions.

### 4.2. Bioreactor Cultivation Runs

Three bioreactor experiments, referred to as PRSR1, PRSR2, and PRSR3, were carried out in the study. In each of them, three stirred tank BIOSTAT^®^ B bioreactors (Sartorius, Germany) were used simultaneously. The inocula for the three bioreactors were as follows: 150 mL of *P. rubens* spore suspension (*P. rubens* monoculture bioreactor), 150 mL of *S. rimosus* spore suspension (*S. rimosus* monoculture bioreactor), and the combined spore suspensions (150 + 150 = 300 mL) of *P. rubens* and *S. rimosus* (coculture bioreactor). The working volume of each bioreactor was 5.5 L. The dissolved oxygen level was maintained at 20% and controlled in cascade by the airflow rate and stirring speed values. The minimum and maximum airflow rates were 1.5 L min^−1^ and 5.5 L min^−1^, respectively, while the minimum and maximum stirring speeds were 220 and 330 min^−1^, respectively. The experiments were performed in three replicates.

### 4.3. Coculture Initiation

*P. rubens* and *S. rimosus* were introduced into the bioreactors as spore suspensions that constituted the inocula. The concentration of spores in each inoculum was carefully controlled with the use of a Thoma chamber to reach the final concentration of spores in the bioreactor within the range from 9 × 10^8^ to 2 × 10^9^ spores per liter. The coculture initiation time depended on the experiment. In PRSR1, *P. rubens* and *S. rimosus* spores were introduced simultaneously. In PRSR2, the inoculation time of *S. rimosus* was delayed by 24 h relative to the inoculation of *P. rubens*. In PRSR3, the inoculation time of *S. rimosus* was delayed by 48 h relative to the inoculation of *P. rubens*. In each of the experiments, the inoculation of *S. rimosus* to initiate both its monoculture and coculture was carried out at the same moment.

### 4.4. Medium Composition

The initial composition of the medium in the three PRSR experiments was the same for each bioreactor (monocultures and cocultures). Glucose (10 g L^−1^) and lactose (40 g L^−1^) served as carbon sources. Yeast extract (10 g L^−1^) was used as a nitrogen source. Phenylacetic acid at 0.25 g L^−1^ was added to the medium as the precursor for penicillin G biosynthesis. The other ingredients in the medium were KH_2_PO_4_ 1.51 g L^−1^, NaCl 0.4 g L^−1^, MgSO_4_·7H_2_O 0.5 g L^−1^, ZnSO_4_·7H_2_O 1 g L^−1^, Fe(NO_3_)_3_·9H_2_O 2 g L^−1^, and biotin 0.04 mg L^−1^. The trace element solution was added at a concentration of 1 mL L^−1^ and contained 50 mg L^−1^ MnSO_4_, 100 mg L^−1^ Na_2_B_4_O_7_·10H_2_O, 250 mg L^−1^ CuSO_4_·5H_2_O, and 50 mg L^−1^ Na_2_MoO_4_·H_2_O. The initial C/N ratio of the cultivation medium was equal to 21.8. The medium was sterilized at 121 °C.

### 4.5. Analytical Methods

Samples of the cultivation broth were drawn every 24 h during the experiment. The samples were filtered with Munktell filter discs (grade 389.84 g m^−2^, diameter 150 mm) and stored at −20 °C. The filter discs were dried at 110 °C and weighed on a laboratory balance before and after filtration. The weights were recorded to calculate the biomass concentration on a dry mass basis. Filtrate analysis was conducted by using ultrahigh-performance liquid chromatography (AQUITY-UPLC^®^) with the use of a BEH Shield RP18 column in reversed-phase (2.1 mm × 100 mm × 1.7 μm) paired with high-resolution mass spectrometry (ACQUITY–SYNAPT G2, Waters, Milford, MA, USA). The eluent flow rate was 0.2 mL min^−1^, and the column temperature was kept at 40 °C. The acetonitrile:water gradient (both acidified with formic acid 0.1% *v*/*v*) was as follows: 0.0–2.5 min, 0:100 (*v*/*v*); 2.5–3.5 min, 20:80 (*v*/*v*); 3.5–4.5 min, 30:70 (*v*/*v*); 4.5–6.8 min, 40:60 (*v*/*v*); 6.8–14.0 min, 60:40 (*v*/*v*).

The direct injection of the filtered supernatant was performed. Before injection, the samples were filtered with a 0.2 μm syringe filter. The monoisotopic masses of ions were investigated with mass spectrometry. Two ionization modes were used: positive (ESI^+^) and negative (ESI^−^). The parameters of analysis were as follows: temperature of source of ions: 120 °C, desolvation temperature for ESI^+^: 200 °C, desolvation temperature for ESI^−^: 400 °C, desolvation gas flow rate (nitrogen) for ESI^+^: 500 L h^−1^, desolvation gas flow rate (nitrogen) for ESI^−^: 1000 L h^−1^, voltage on the capillary: 3 kV, sampling cone voltage: 40 V, extraction cone voltage: 4 V.

Identification was based on the monoisotopic value of *m/z*. The absolute error Δ(*m/z*) was determined by subtracting the theoretical *m/z* value from the experimental *m/z* value. It was assumed that the absolute error had to be lower than |±0.009|. The identification of metabolites was performed at four levels of confidence. The first level, applied in the cases of oxytetracycline and penicillin G, was the confirmation of identity with the use of purchased standards. The second level, applied in the cases of rimocidin, CE-108, rimocidin B, 2-acetyl-2-decarboxamido-oxytetracycline, rimosamide A, chrysogine and benzylpenicilloic acid, was based on the agreement of experimental *m/z* values with the ones exhibited by the secondary metabolites previously found in the same species (i.e., *S. rimosus* or *P. rubens*). The Natural Product Atlas [58] served as a reference database during the identification process. The third level encompassed the identifications based on the agreement of *m/z* values with the ones exhibited by the secondary metabolites previously found in the same genus (i.e., *Streptomyces* in the case of *S. rimosus* metabolites or *Penicillium* in the case of *P. rubens* metabolites). The fourth level, displaying the lowest level of confidence in the study, was applied only in the case of 2-methylthio-cis-zeatin, which was previously found in a microorganism belonging to the same class as *Streptomyces*, namely in *Actinomycetia*. In addition, the standard identification levels were reported according to the recommendations of the Metabolomics Standards Initiative [59]: Level 1 (“identified compounds”) was assigned for penicillin G and oxytetracycline (based on retention times, accurate *m/z* values and fragmentation patterns) and level 2 (“putatively annotated compounds”) was assigned for the remaining secondary metabolites.

Metabolites were investigated semiquantitatively by considering their peak areas of [M − H]^−^ and [M + H]^+^ ions. The results of the semiquantitative analysis, i.e., peak area values, were reported in “auxiliary units” (AU) and represented the absolute amounts of metabolites in the broth. The analysis was conducted with the use of TargetLynx (Waters, Version VMassLynx SCN781, Milford, MA, USA) software. For penicillin G and oxytetracycline, quantitative analysis was performed, and the concentration values were reported in “mg L^−1^” units. Standards of penicillin G (as potassium salt) and oxytetracycline (as hydrochloride) were purchased from Sigma-Aldrich (Burlington, MA, USA). 

The glucose and lactose concentrations in the filtrates were investigated simultaneously with the use of ultrahigh-performance liquid chromatography (AQUITY-UPLC^®^) by applying a BEH Amide column in the normal phase (2.1 mm × 150 mm × 1.7 μm) paired with an Evaporative Light Scattering Detector (Waters, Waters, Milford, MA, USA). Carbohydrates were eluted with 75% acetonitrile dissolved in deionized water enriched with 0.2% triethylamine. The parameters of the analysis were as follows: eluent flow rate of 0.29 mL min^−1^, column temperature of 35 °C. The accuracy of carbohydrate analysis was ±0.85 g L^−1^. The sensitivity of the semiquantitative analysis was 10 auxiliary units (AU). This level allowed us to confirm the presence of a given secondary metabolite in the cultivation broth. PTC Mathcad 15 software was employed to calculate the volumetric uptake rates of glucose and lactose, r_GLU_, and r_LAC_, respectively, according to the following procedure. The concentration data were approximated with the use of the cubic b-spline function, and the smooth curves were then differentiated in time to determine the temporal changes in r_GLU_ and r_LAC_.

### 4.6. Morphological Analysis

Microscopic observations and morphological analyses were carried out using an OLYMPUS BX53 microscope (Olympus Corporation, Tokyo, Japan) coupled with OLYMPUS cellSens Dimension Desktop 1.16 image analysis software (Olympus Corporation, Version 1.16, Tokyo, Japan). The values of the projected area (A), elongation (E), and morphology number (Mo) were obtained as previously described [41]. The mean values of morphological parameters and the corresponding confidence bands were calculated on the basis of at least 30 hyphal objects. Student’s *t*-distribution (significance level α = 0.05) was employed to determine the confidence bands.

### 4.7. Statistical Analysis

The data confidence function in OriginPro 2017 software (OriginLab, Version b9.4.1.354 SR1, Northampton, MA, USA) was employed to evaluate the replicability of the experimental results. This function provides the confidence interval for the mean value. A significance level of *α* = 0.05 was applied in the statistical calculations. The analysis of replicability was carried out by considering the levels of secondary metabolites produced by *S. rimosus* (Figure S1), the levels of *P. rubens* secondary metabolites (Figure S2), and the sugar concentration values (Figure S3) recorded in the bioreactor monocultures.

## Figures and Tables

**Table 1:** Secondary metabolites of *S. rimosus* and *P. rubens* identified or putatively annotated in the study.

Ionization	(*m/z*)_experimental_	Retention Time (min)	Suggested Secondary Metabolite	Formula of Suggested Metabolite (Ionized)	(*m/z*)_theoretical_	Absolute Error Δ(*m/z*)	Microbial Source	Level of Metabolite Identification
ESI^−^	459.1427	4.33	Oxytetracycline [21]	C_22_H_23_N_2_O_9_	459.1404	+0.0023	*S. rimosus*	identified
ESI^−^	766.3990	5.91	Rimocidin [22]	C_39_H_60_NO_14_	766.4014	−0.0024	*S. rimosus*	putatively annotated
ESI^−^	738.3635	5.46	CE-108 [23]	C_37_H_56_NO_14_	738.3701	−0.0066	*S. rimosus*	putatively annotated
ESI^−^	765.4189	5.78	Rimocidin B [24]	C_39_H_61_N_2_O_13_	765.4174	+0.0015	*S. rimosus*	putatively annotated
ESI^−^	752.3879	5.68	Rimocidin (27-ethyl) [20]	C_38_H_58_NO_14_	752.3857	+0.0022	*S. rimosus*	putatively annotated
ESI^−^	527.2993	5.70	Milbemycin A_3_ [25]	C_31_H_43_O_7_	527.3009	−0.0016	*S. rimosus*	putatively annotated
ESI^−^	591.2823	6.15	Milbemycin A_3_ + [4O] [20]	C_31_H_43_O_11_	591.2805	+0.0018	*S. rimosus*	putatively annotated
ESI^−^	593.3038	6.67	Milbemycin β_11_ + [4O] [20]	C_31_H_45_O_11_	593.2962	+0.0076	*S. rimosus*	putatively annotated
ESI^−^	619.3103	6.91	Turgichelin [26]	C_24_H_43_N_8_O_11_	619.3051	−0.0052	*S. rimosus*	putatively annotated
ESI^−^	264.0859	4.95	2-Methylthio-cis-zeatin [27]	C_11_H_14_N_5_OS	264.0919	−0.0060	*S. rimosus*	putatively annotated
ESI^−^	603.3022	5.29	Rimosamide A [28]	C_30_H_43_N_4_O_9_	603.3030	−0.0008	*S. rimosus*	putatively annotated
ESI^+^	708.3666	5.23	Lucensomycin [29]	C_36_H_54_NO_13_	708.3595	+0.0071	*S. rimosus*	putatively annotated
ESI^−^	263.1064	4.68	Spinoxazine A [30]	C_13_H_15_N_2_O_4_	263.1032	+0.0032	*S. rimosus*	putatively annotated
ESI^−^	384.1161	4.95	Unnamed angucycline metabolite previously isolated from *Streptomyces* sp. QL37 [31]	C_20_H_18_O_7_N	384.1083	+0.0078	*S. rimosus*	putatively annotated
ESI^−^	458.1409	4.68	2-Acetyl-2-decarboxamido-oxytetracycline (ADOTC) [32]	C_23_H_24_NO_9_	458.1451	−0.0042	*S. rimosus*	putatively annotated
ESI^−^	724.3525	5.24	7-Demethyl-glucopiericidin A [33]	C_36_H_54_NO_14_	724.3544	-0.0019	*S. rimosus*	putatively annotated
ESI^−^	333.0928	6.47	Penicillin G [34]	C_16_H_17_N_2_O_4_S	333.0909	+0.0019	*P. rubens*	identified
ESI^−^	351.0989	5.72	Benzylpenicilloic acid [35]	C_16_H_19_N_2_O_5_S	351.1015	-0.0026	*P. rubens*	putatively annotated
ESI^−^	189.0756	4.72	Chrysogine [36]	C_10_H_9_N_2_O_2_	189.0665	+0.0091	*P. rubens*	putatively annotated
ESI^−^	710.0464	4.86	Adenophostin B [37]	C_18_H_27_N_5_O_19_P_3_	710.0513	−0.0049	*P. rubens*	putatively annotated
ESI^−^	335.1042	6.48	Cyclopiamide D [38]	C_19_H_15_N_2_O_4_	335.1031	+0.0011	*P. rubens*	putatively annotated
ESI^−^	491.2644	8.30	Preaustinoid D [39]	C_27_H_39_O_8_	491.2645	−0.0001	*P. rubens*	putatively annotated

## Data Availability

The data used to support the findings of this study are available from the corresponding author upon request.

## Supplementary Materials

The following supporting information can be downloaded at: https://www.mdpi.com/article/10.3390/molecules28166044/s1, Figures S1–S3: replicability analysis; Figures S4–S30: alignments of total ion chromatograms; Figures S31–S37: production profiles of secondary metabolites; Figures S38 and S39: time courses of morphological parameter values; Figure S40: selected microscopic images.

## Sample Availability

Samples of the compounds and strains are available from the authors.
