# Supplementary material for: Effects of the Coculture Initiation Method on the Production of Secondary Metabolites in Bioreactor Cocultures of Penicillium rubens and Streptomyces rimosus
Source: Molecules. 2023 Aug 13;28(16):6044. doi: 10.3390/molecules28166044 (PMC10458595; doi:10.3390/molecules28166044)
Supplement: Supplementary file 1 [file molecules-28-06044-s001.zip › molecules-2466083-supplementary.pdf]

molecules

MDPI

Article

Effects of the Coculture Initiation Method on the Production of Secondary Metabolites in Bioreactor Cocultures of *Penicillium rubens* and *Streptomyces rimosus*

Tomasz Boruta *, Anna Ścigaczewska, Agnieszka Ruda and Marcin Bizukojć

Department of Bioprocess Engineering, Faculty of Process and Environmental Engineering, Lodz University of Technology, ul. Wolczanska 213, 93-005 Lodz, Poland; anna.kowalska.1@p.lodz.pl (A.Ś.); 222829@edu.p.lodz.pl (A.R.); marcin.bizukojc@p.lodz.pl (M.B.)

* Correspondence: tomasz.boruta@p.lodz.pl, Tel.: +48-42-631-39-77; Fax: +48-42-636-56-63

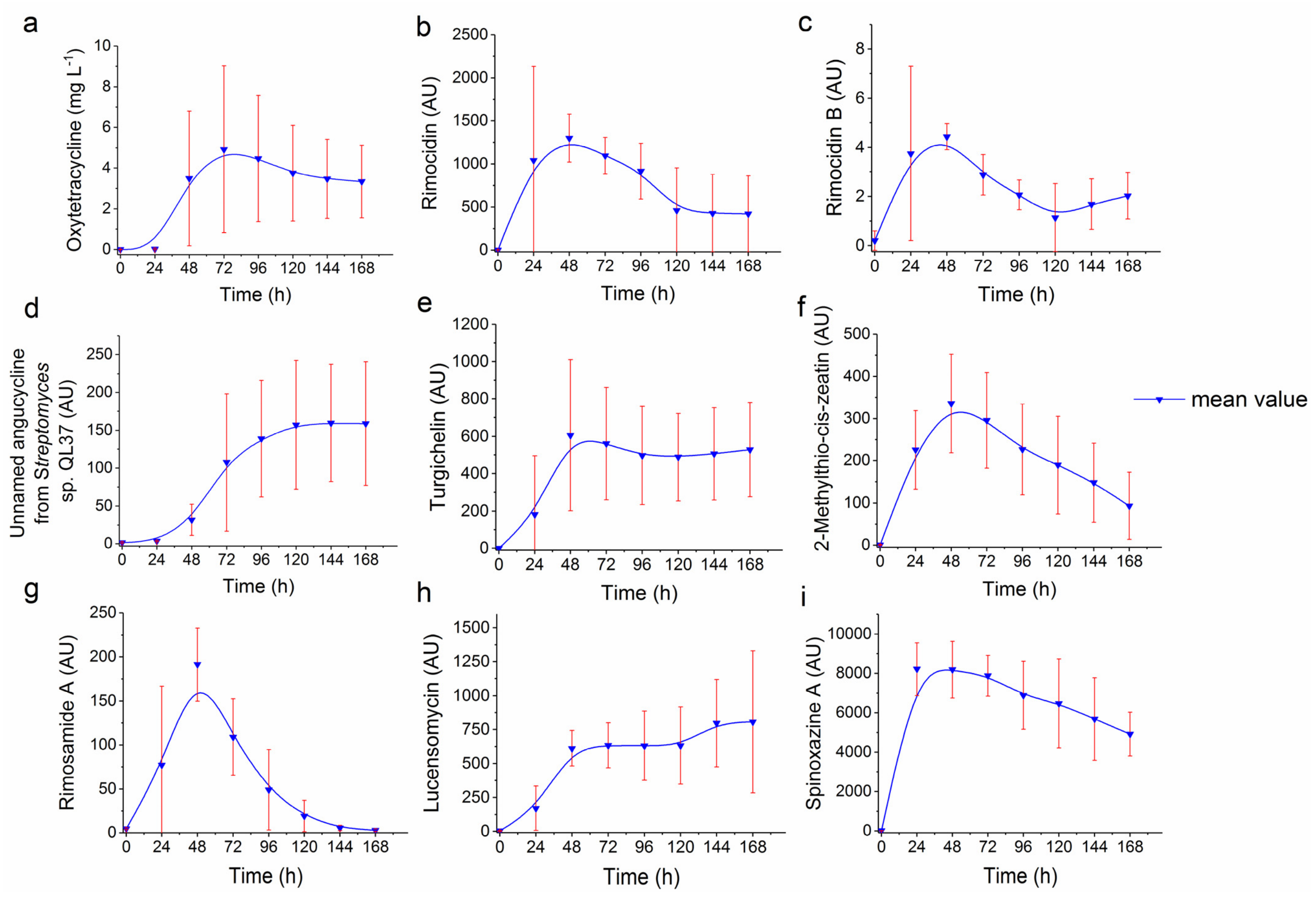

Figure S1. Replicability analysis of the levels of secondary metabolites produced by *S. rimosus*, namely, oxytetracycline (a) and the metabolites tentatively identified as rimocidin (b), rimocidin B (c), unnamed angucycline from *Streptomyces* sp. QL37 (d), turgichelin (e), 2-methylthio-cis-zeatin (f), rimosamide A (g), lu-

censomycin (h), and spinoxazine A (i). The confidence intervals for the mean values are depicted. A significance level of α=0.05 was used for the calculations. The replicability analysis was based on the data collected during the PR◎R1, PR◎R2 and PR◎R3 monocultures of *S. rimosus*. AU-auxiliary units.

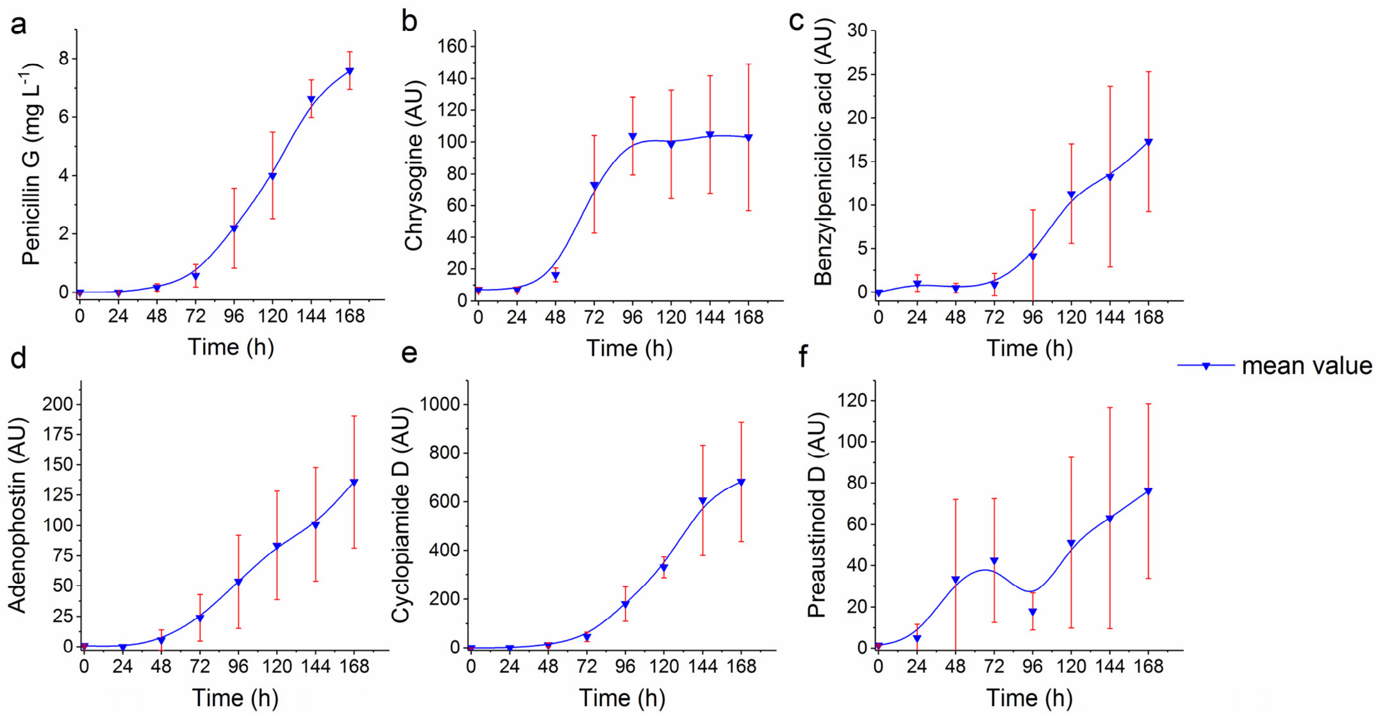

Figure S2. Replicability analysis of the levels of secondary metabolites produced by *P. rubens*, namely, penicillin G (a) and the metabolites tentatively identified as chrysogine (b), benzylpenicilloic acid (c), adenophostin B (d), cyclopiamide D (e), and preaustinoid D (f). The confidence intervals for the mean values are depicted. A significance level of α=0.05 was used for the calculations. The replicability analysis was based on the data collected during the PR◎R1, PR◎R2 and PR◎R3 monocultures of *P. rubens*. AU-auxiliary units.

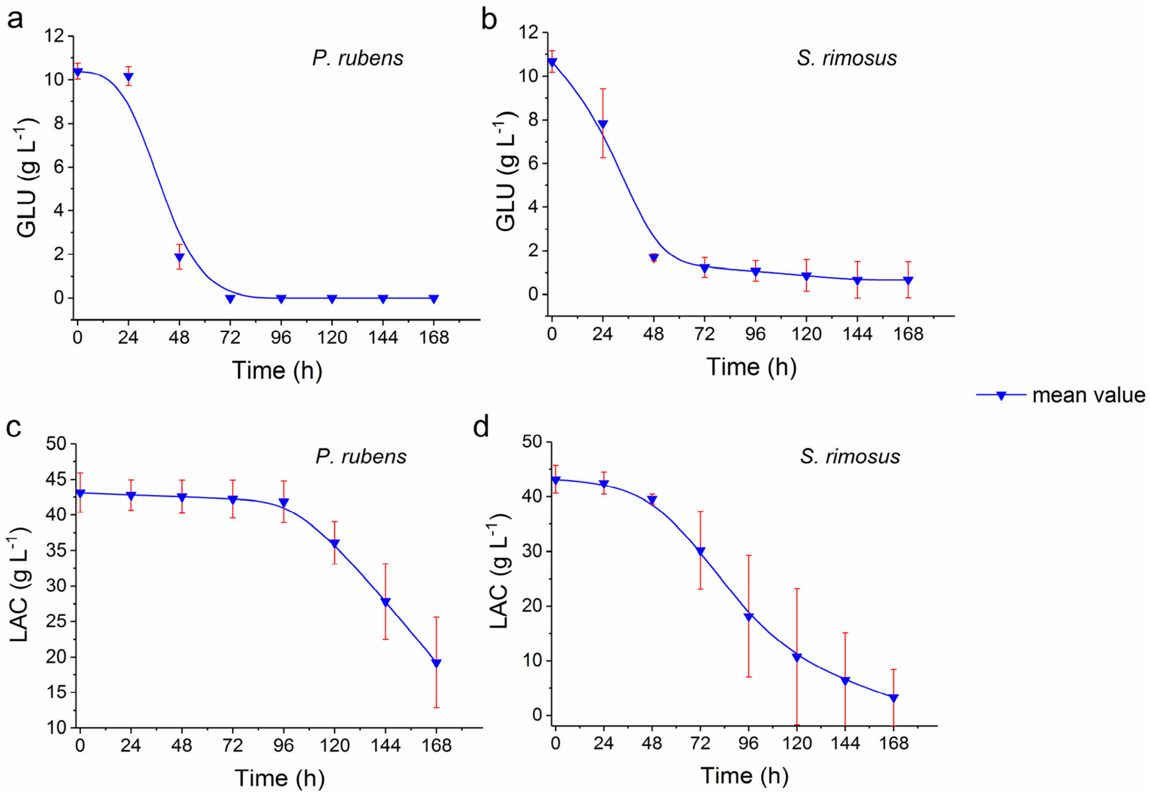

Figure S3. Replicability analysis of the levels of glucose (a, b) and lactose (c, d) in the monocultures of *P. rubens* (a, c) and *S. rimosus* (b, d). The confidence intervals for the mean values are depicted. A significance level of α=0.05 was used for the calculations. The replicability analysis was based on the data collected during the PR◎R1, PR◎R2 and PR◎R3 monocultures of *P. rubens* and *S. rimosus*. AU-auxiliary units.

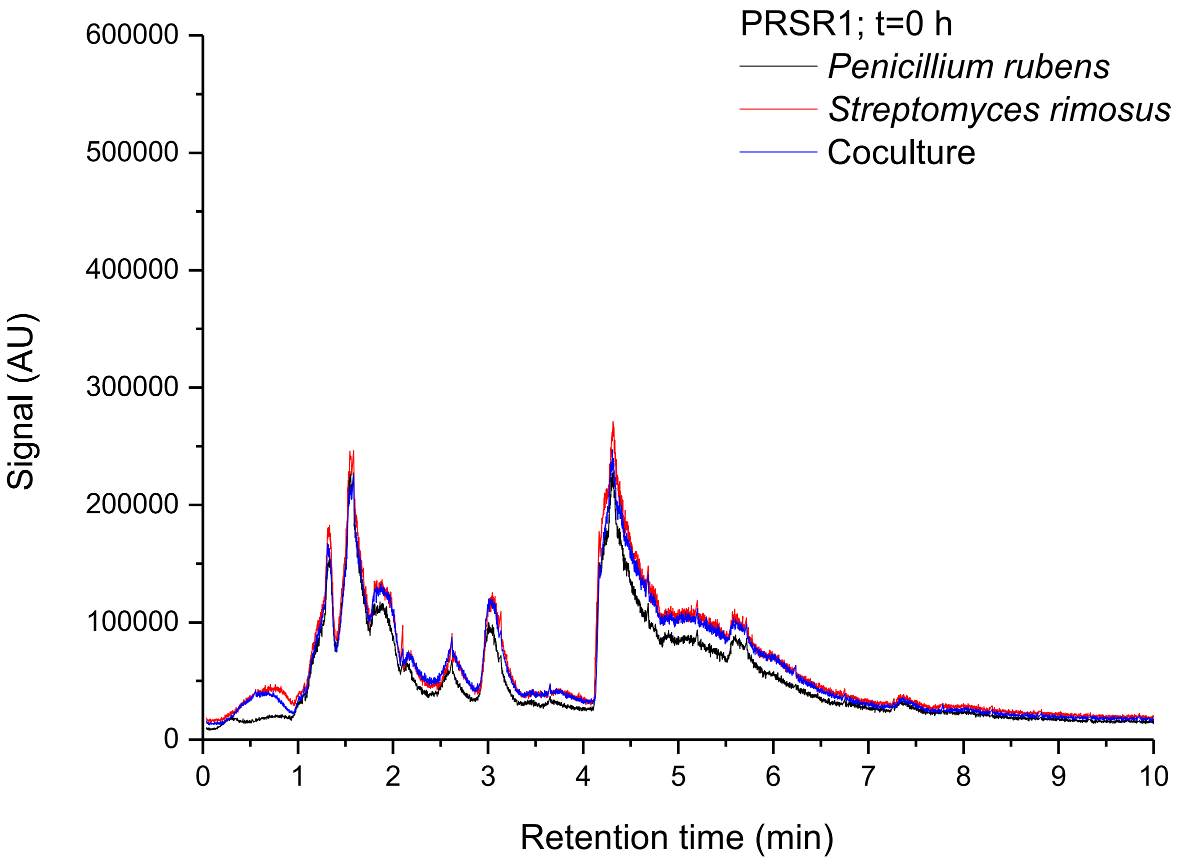

Figure S4 Alignments of total ion chromatograms (TICs) recorded in the PR⊚R1 experiment at t=0 h for *P. rubens* monoculture (black line), *S. rimosus* monoculture (red line), and the "*P. rubens* vs. *S. rimosus*" coculture (blue line). AU-auxiliary units.

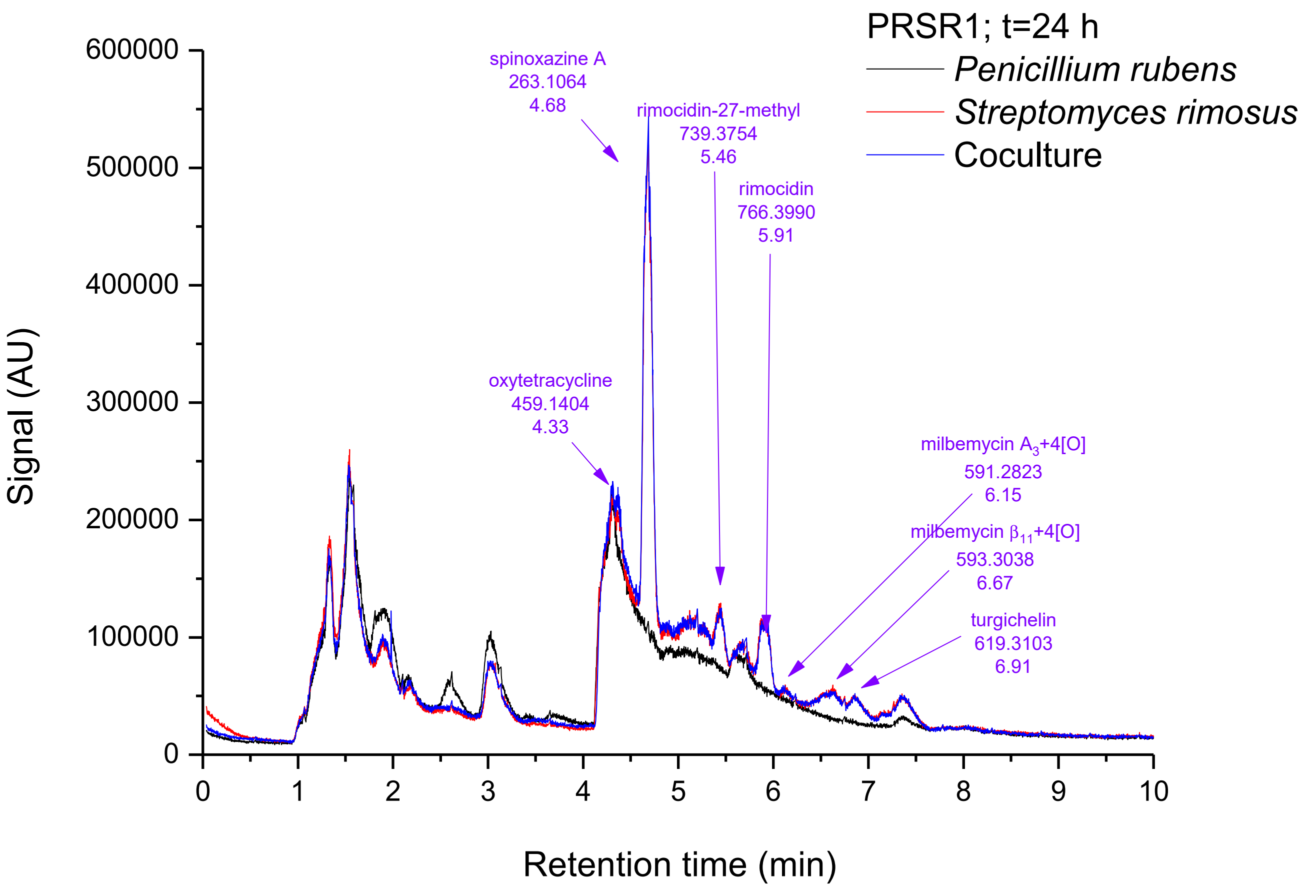

Figure S5 Alignments of total ion chromatograms (TICs) recorded in the PR⊚R1 experiment at t=24 h for *P. rubens* monoculture (black line), *S. rimosus* monoculture (red line), and the "*P. rubens* vs. *S. rimosus*" coculture (blue line). AU- auxiliary units. The names of metabolites that were found both in the monoculture of *S. rimosus* and the coculture are shown in violet. The metabolites were tentatively identified on the basis of M⊚ data, however, their identities were not confirmed due to a lack of analytical standards. The exception was the confirmed identity of oxytetracycline.

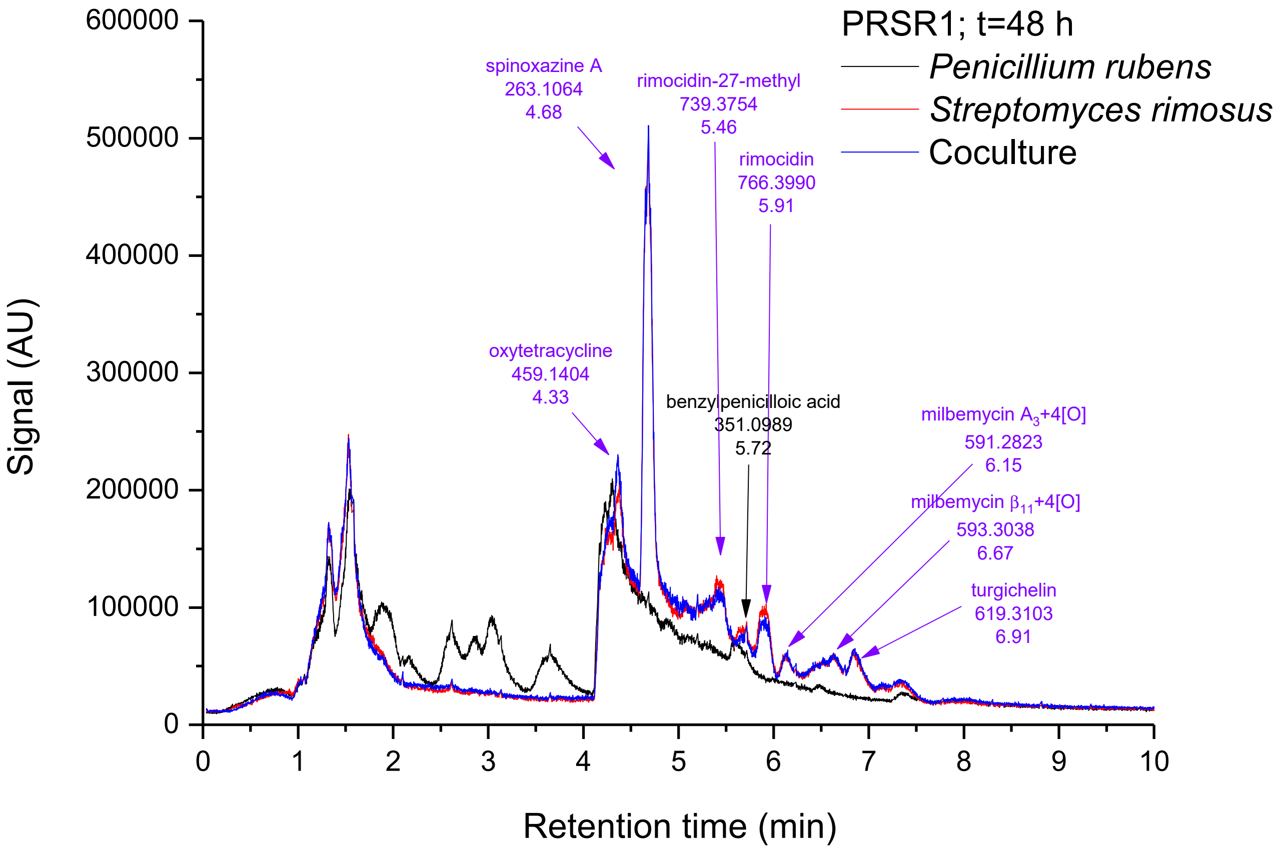

Figure S6 Alignments of total ion chromatograms (TICs) recorded in the PR◎R1 experiment at t=48 h for *P. rubens* monoculture (black line), *S. rimosus* monoculture (red line), and the "*P. rubens* vs. *S. rimosus*" coculture (blue line). AU-auxiliary units. The names of metabolites that were found both in the monoculture of *S. rimosus* and the coculture are shown in violet. The name of the metabolite that was found solely in the monoculture of *P. rubens* is shown in black. The metabolites were tentatively identified on the basis of M◎ data, however, their identities were not confirmed due to a lack of analytical standards. The exception was the confirmed identity of oxytetracycline.

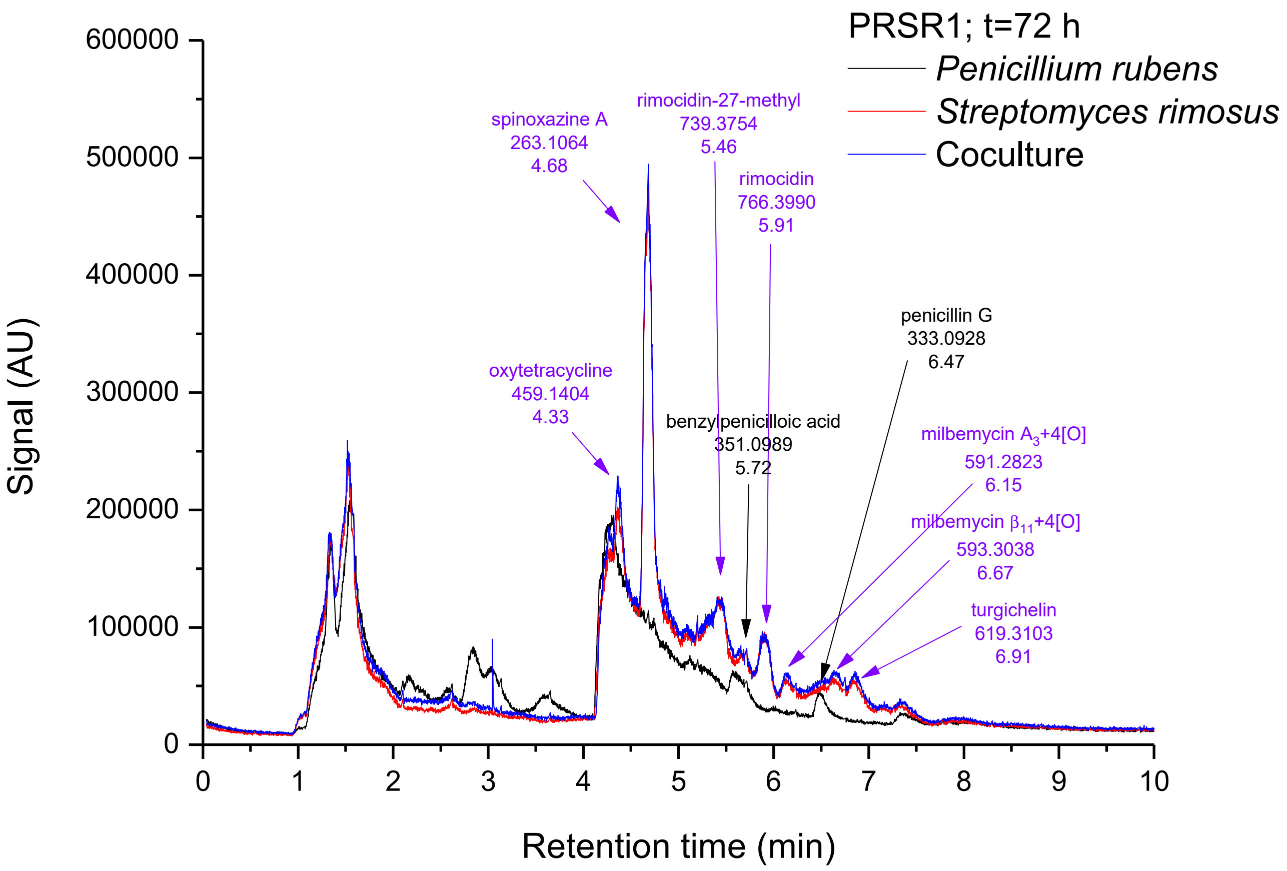

Figure S7 Alignments of total ion chromatograms (TICs) recorded in the PR⊚R1 experiment at t=72 h for *P. rubens* monoculture (black line), *S. rimosus* monoculture (red line), and the "*P. rubens* vs. *S. rimosus*" coculture (blue line). AU- auxiliary units. The names of metabolites that were found both in the monoculture of *S. rimosus* and the coculture are shown in violet. The names of the metabolites that were found solely in the monoculture of *P. rubens* are shown in black. The metabolites were tentatively identified on the basis of M⊚ data, however, their identities were not confirmed due to a lack of analytical standards. The exceptions were the confirmed identities of oxytetracycline and penicillin G.

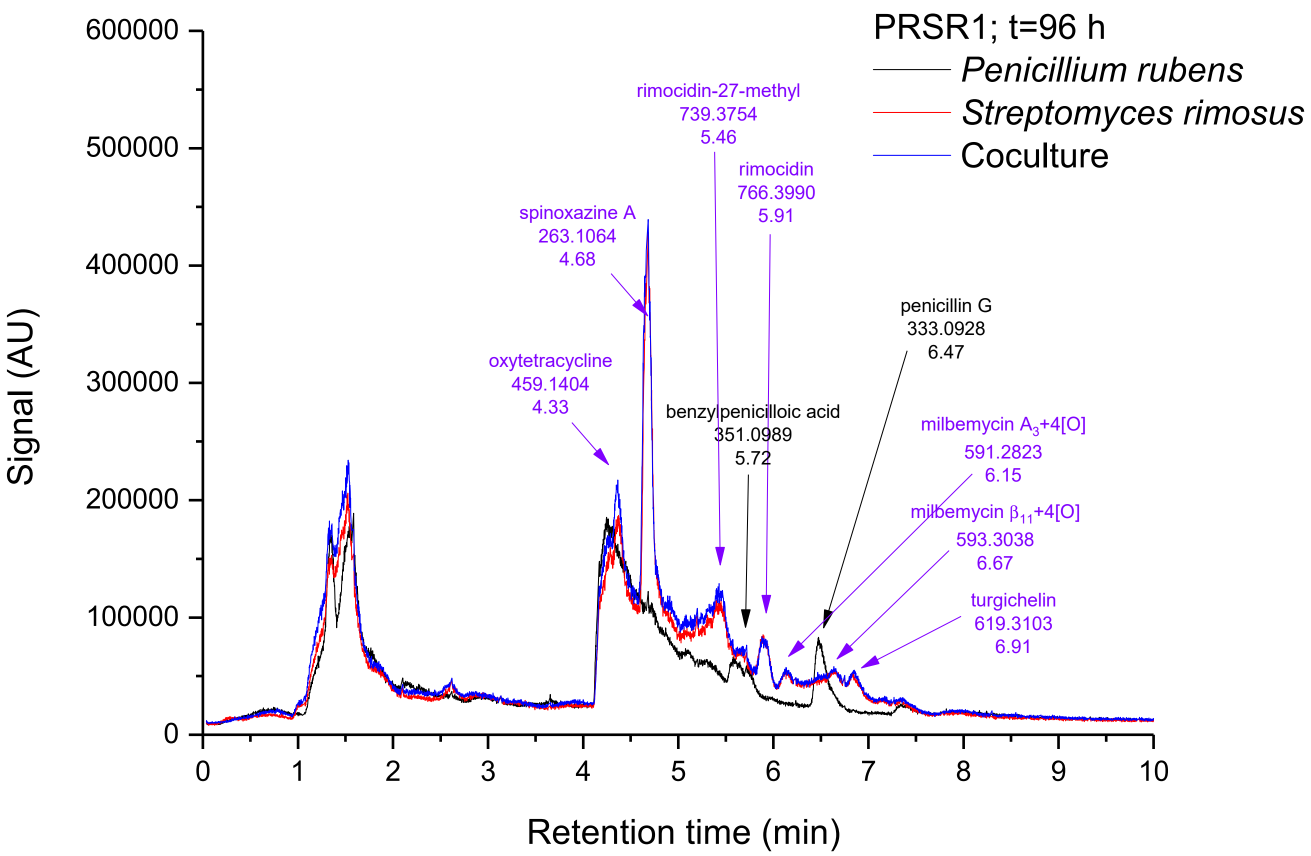

Figure S8 Alignments of total ion chromatograms (TICs) recorded in the PR⊚R1 experiment at t=96 h for *P. rubens* monoculture (black line), *S. rimosus* monoculture (red line), and the "*P. rubens* vs. *S. rimosus*" coculture (blue line). AU-auxiliary units. The names of metabolites that were found both in the monoculture of *S. rimosus* and the coculture are shown in violet. The names of the metabolites that were found solely in the monoculture of *P. rubens* are shown in black. The metabolites were tentatively identified on the basis of M⊚ data, however, their identities were not confirmed due to a lack of analytical standards. The exceptions were the confirmed identities of oxytetracycline and penicillin G.

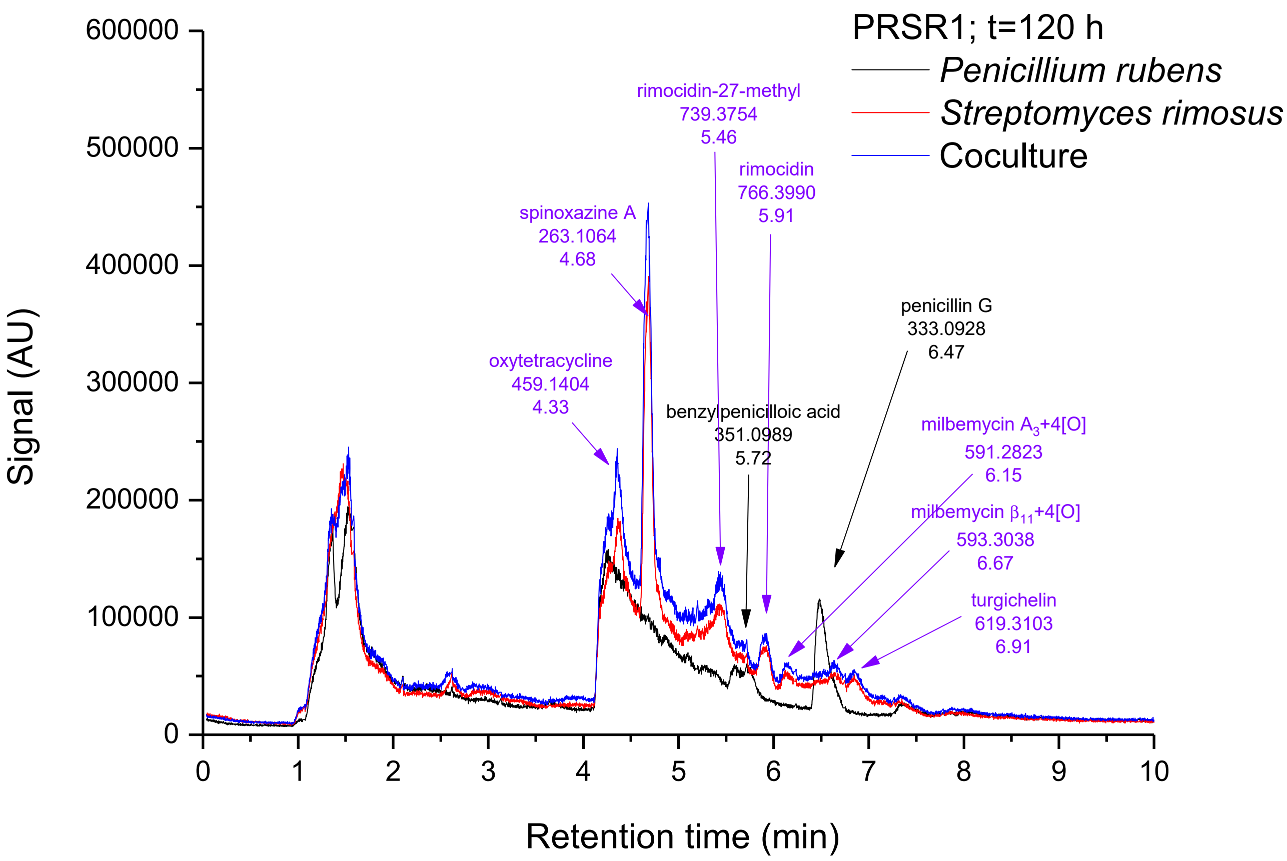

Figure S9 Alignments of total ion chromatograms (TICs) recorded in the PR⊚R1 experiment at t=120 h for *P. rubens* monoculture (black line), *S. rimosus* monoculture (red line), and the "*P. rubens* vs. *S. rimosus*" coculture (blue line). AU- auxiliary units. The names of metabolites that were found both in the monoculture of *S. rimosus* and the coculture are shown in violet. The names of the metabolites that were found solely in the monoculture of *P. rubens* are shown in black. The metabolites were tentatively identified on the basis of M⊚ data, however, their identities were not confirmed due to a lack of analytical standards. The exceptions were the confirmed identities of oxytetracycline and penicillin G.

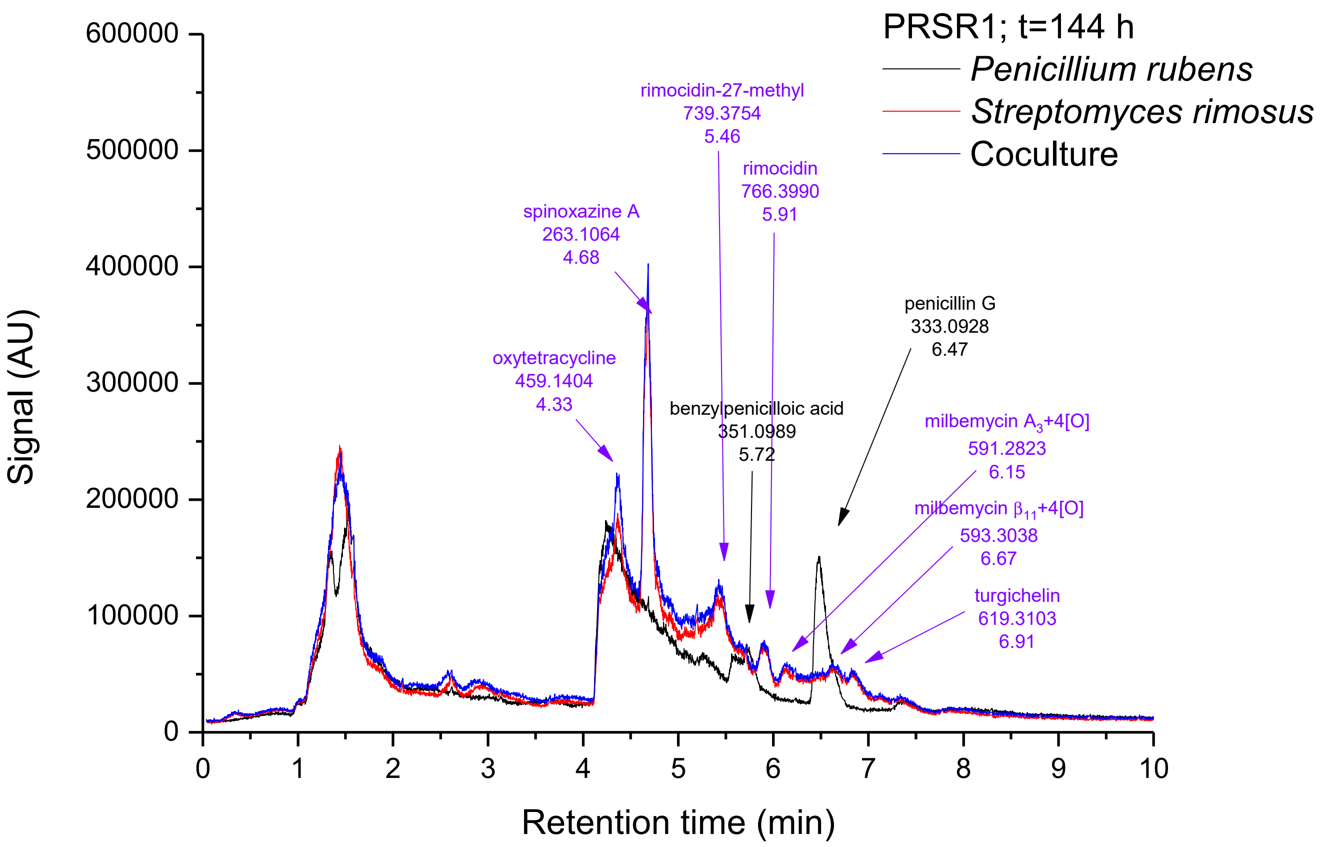

Figure S10 Alignments of total ion chromatograms (TICs) recorded in the PR⊚R1 experiment at t=144 h for *P. rubens* monoculture (black line), *S. rimosus* monoculture (red line), and the "*P. rubens* vs. *S. rimosus*" coculture (blue line). AU-auxiliary units. The names of metabolites that were found both in the monoculture of *S. rimosus* and the coculture are shown in violet. The names of the metabolites that were found solely in the monoculture of *P. rubens* are shown in black. The metabolites were tentatively identified on the basis of M⊚ data, however, their identities were not confirmed due to a lack of analytical standards. The exceptions were the confirmed identities of oxytetracycline and penicillin G.

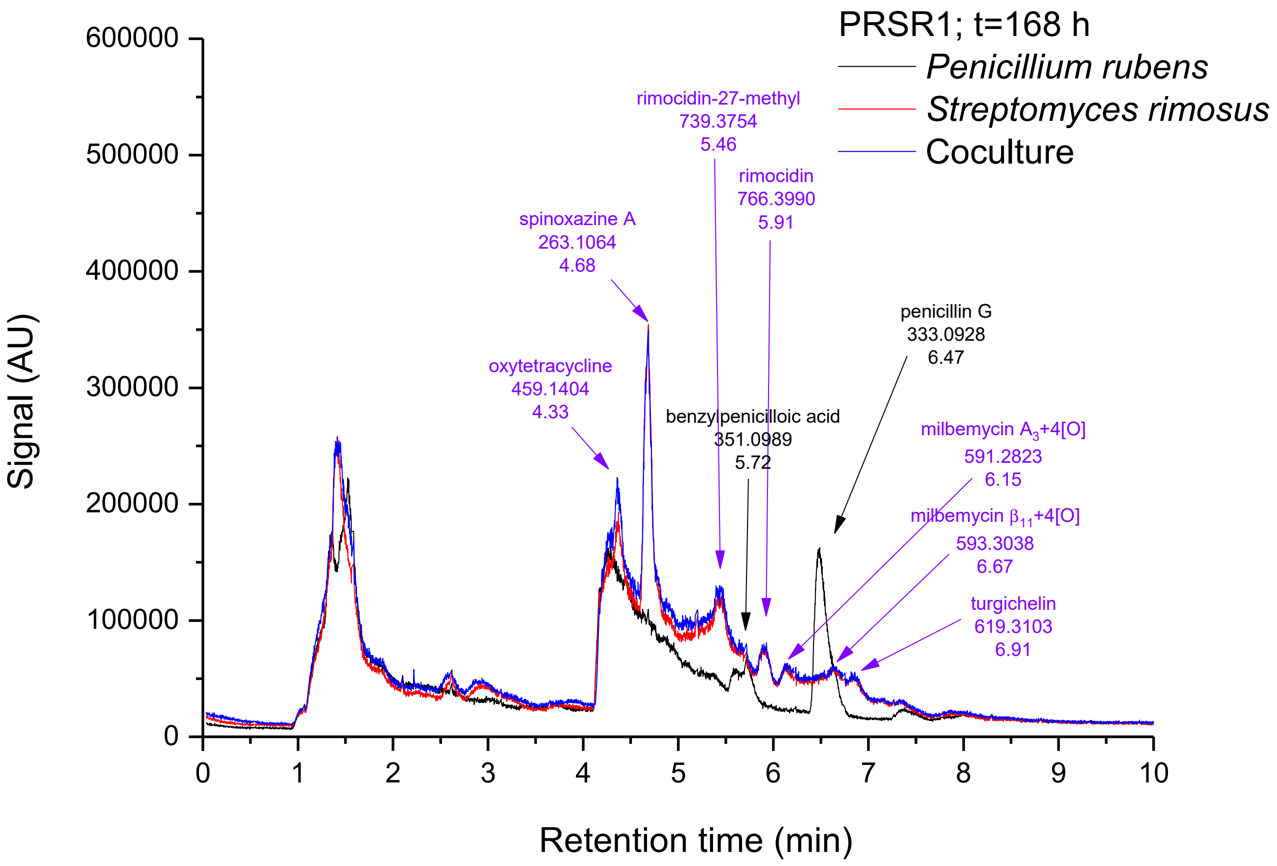

Figure S11 Alignments of total ion chromatograms (TICs) recorded in the PR⊚R1 experiment at t=168 h for *P. rubens* monoculture (black line), *S. rimosus* monoculture (red line), and the "*P. rubens* vs. *S. rimosus*" coculture (blue line). AU-auxiliary units. The names of metabolites that were found both in the monoculture of *S. rimosus* and the coculture are shown in violet. The names of the metabolites that were found solely in the monoculture of *P. rubens* are shown in black. The metabolites were tentatively identified on the basis of M⊚ data, however, their identities were not confirmed due to a lack of analytical standards. The exceptions were the confirmed identities of oxytetracycline and penicillin G.

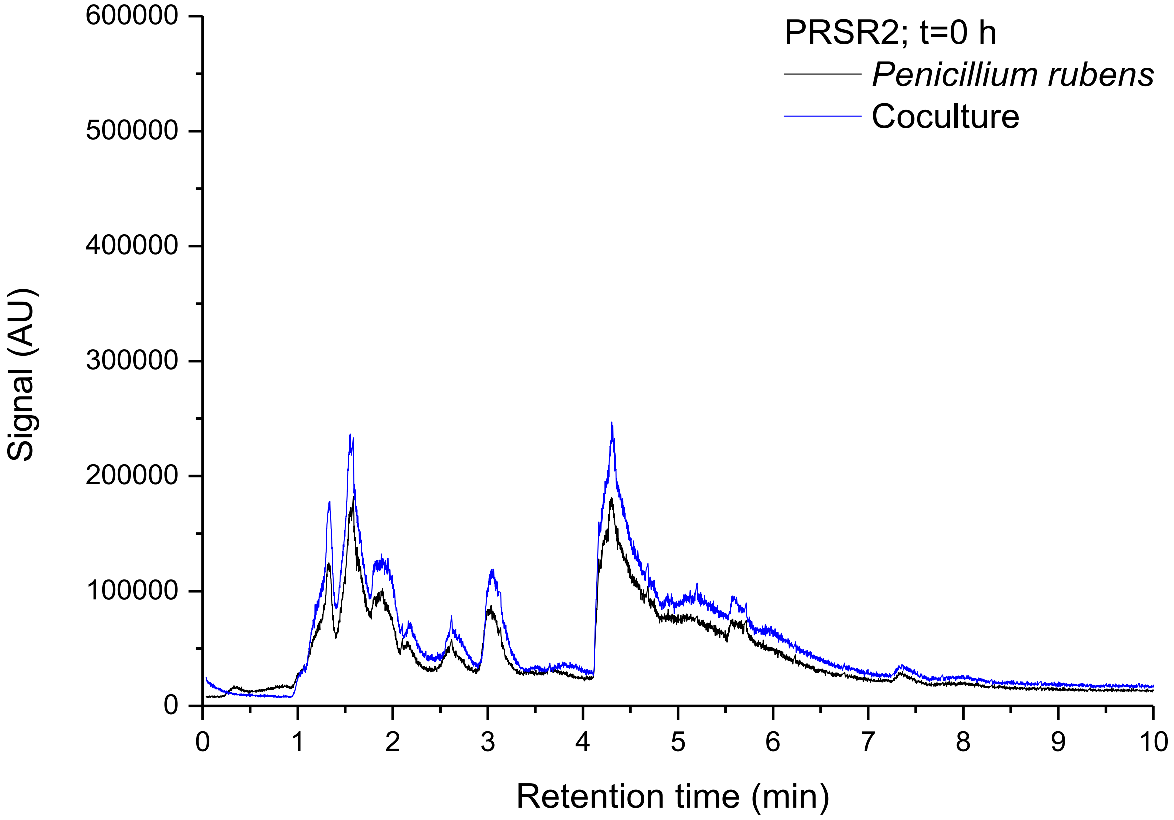

Figure S12 Alignments of total ion chromatograms (TICs) recorded in the PR◎R2 experiment at t=0 h for *P. rubens* monoculture (black line) and the "*P. rubens* vs. *S. rimosus*" coculture (blue line). AU-auxiliary units.

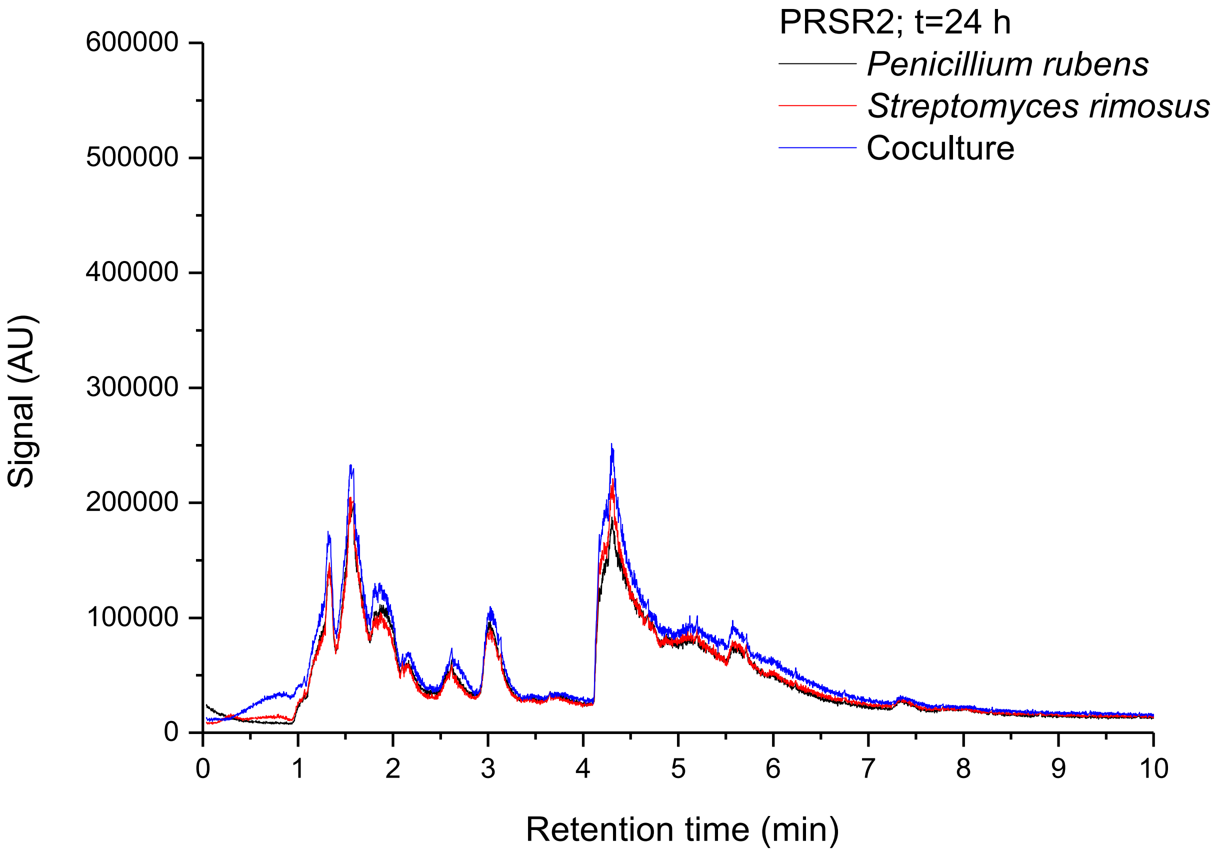

Figure S13 Alignments of total ion chromatograms (TICs) recorded in the PR⊚R2 experiment at t=24 h for *P. rubens* monoculture (black line), *S. rimosus* monoculture (red line), and the "*P. rubens* vs. *S. rimosus*" coculture (blue line). AU-auxiliary units.

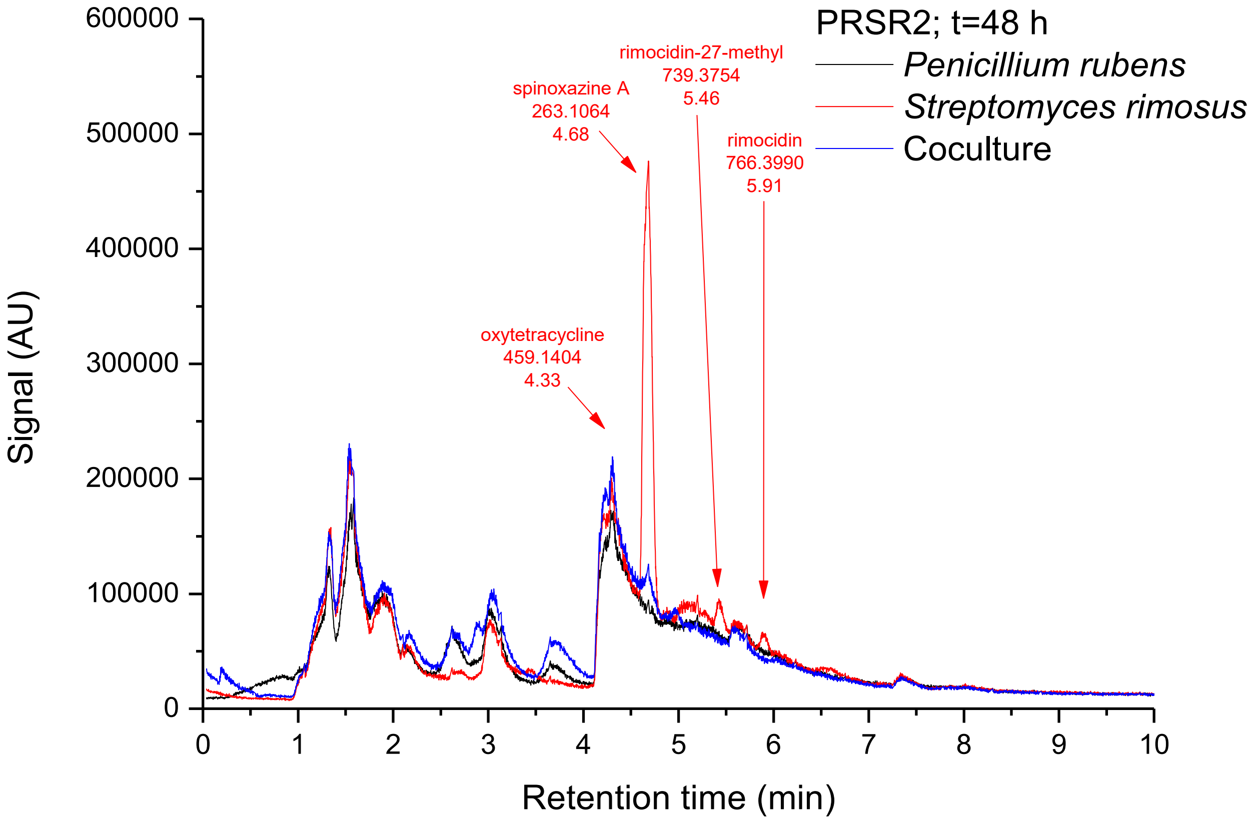

Figure S14 Alignments of total ion chromatograms (TICs) recorded in the PR⊚R2 experiment at t=48 h for *P. rubens* monoculture (black line), *S. rimosus* monoculture (red line), and the "*P. rubens* vs. *S. rimosus*" coculture (blue line). AU-auxiliary units. The names of the metabolites that were found solely in the monoculture of *S. rimosus* are shown in red. The metabolites were tentatively identified on the basis of M⊚ data, however, their identities were not confirmed due to a lack of analytical standards. The exception was the confirmed identity of oxytetracycline.

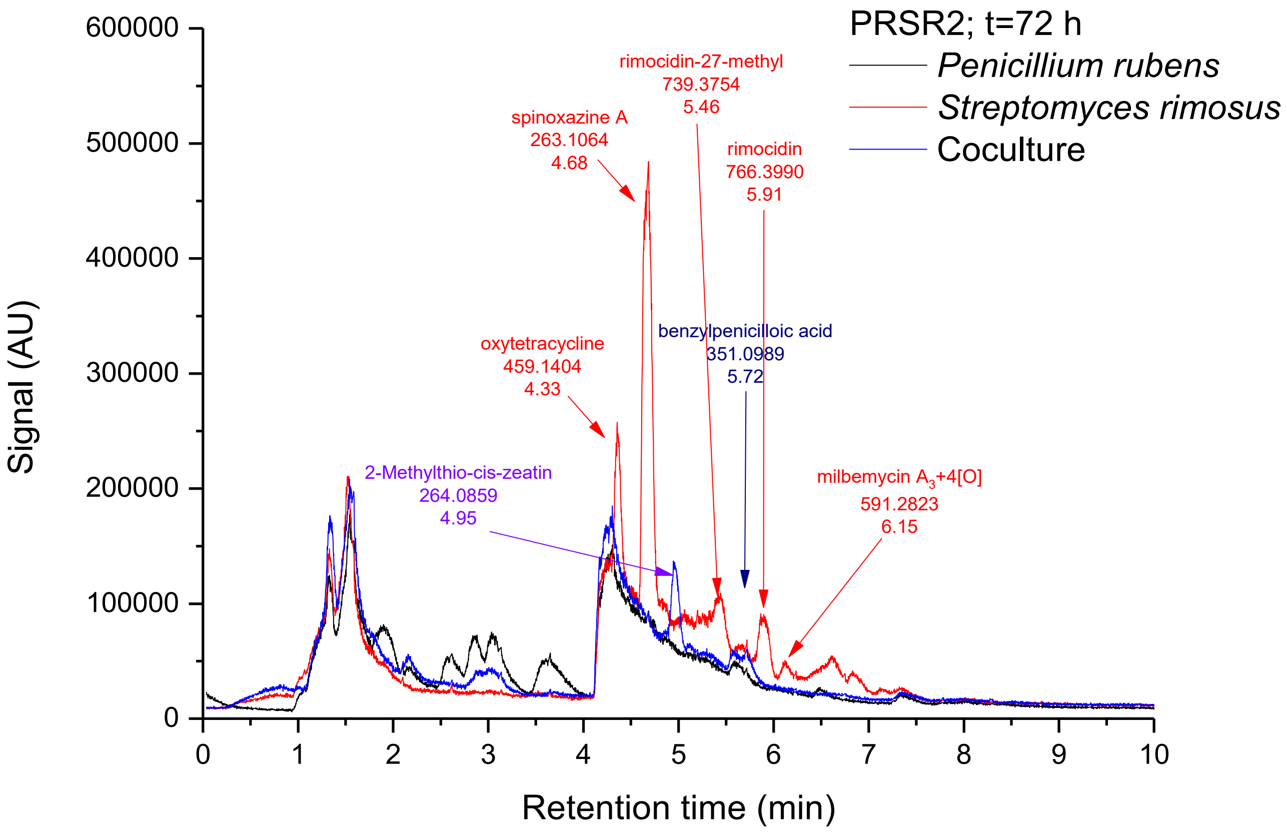

Figure S15 Alignments of total ion chromatograms (TICs) recorded in the PR⊚R2 experiment at t=72 h for *P. rubens* monoculture (black line), *S. rimosus* monoculture (red line), and the "*P. rubens* vs. *S. rimosus*" coculture (blue line). AU- auxiliary units. The names of the metabolites that were found solely in the monoculture of *S. rimosus* are shown in red. The name of the metabolite that was found both in the monoculture of *S. rimosus* and the coculture is shown in violet. The name of the metabolite that was found both in the monoculture of *P. rubens* and the coculture is shown in navy blue. The metabolites were tentatively identified on the basis of M⊚ data, however, their identities were not confirmed due to a lack of analytical standards. The exception was the confirmed identity of oxytetracycline.

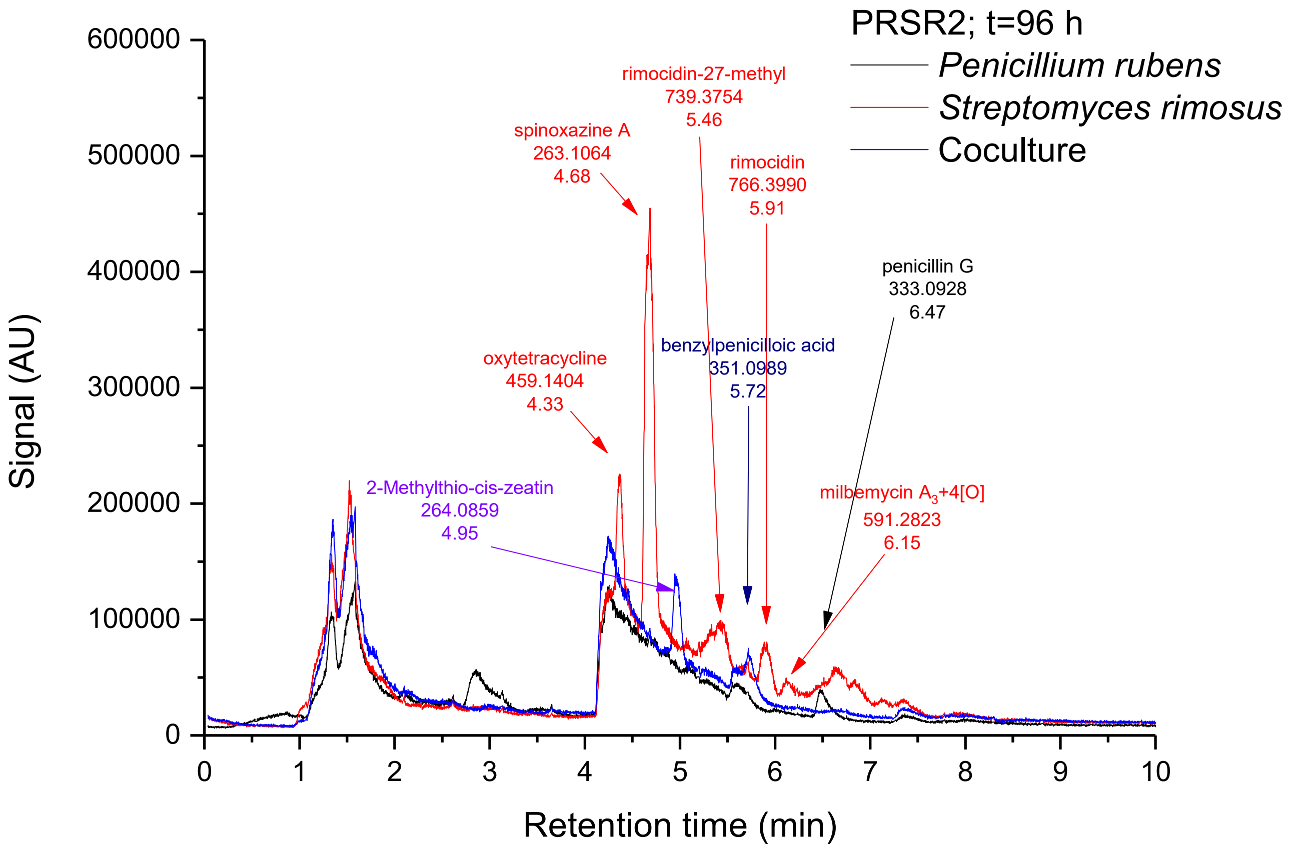

Figure S16 Alignments of total ion chromatograms (TICs) recorded in the PR◎R2 experiment at t=96 h for *P. rubens* monoculture (black line), *S. rimosus* monoculture (red line), and the "*P. rubens* vs. *S. rimosus*" coculture (blue line). AU-auxiliary units. The names of the metabolites that were found solely in the monoculture of *S. rimosus* are shown in red. The name of the metabolite that was found both in the monoculture of *S. rimosus* and the coculture is shown in violet. The name of the metabolite that was found both in the monoculture of *P. rubens* and the coculture is shown in navy blue. The name of the metabolite that was found solely in the monoculture of *P. rubens* is shown in black. The metabolites were tentatively identified on the basis of M◎ data, however, their identities were not confirmed due to a lack of analytical standards. The exceptions were the confirmed identities of oxytetracycline and penicillin G.

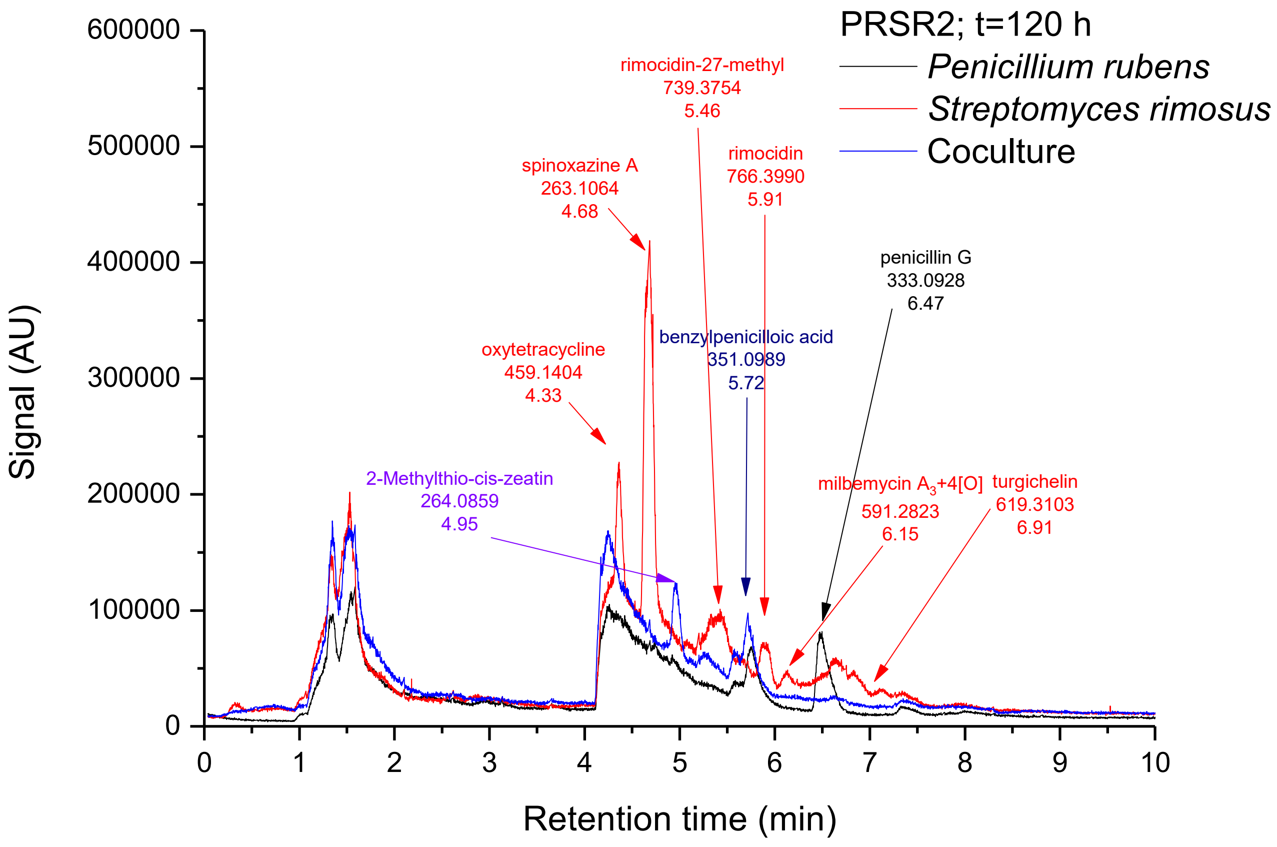

Figure S17 Alignments of total ion chromatograms (TICs) recorded in the PR⊚R2 experiment at t=120 h for *P. rubens* monoculture (black line), *S. rimosus* monoculture (red line), and the "*P. rubens* vs. *S. rimosus*" coculture (blue line). AU-auxiliary units. The names of the metabolites that were found solely in the monoculture of *S. rimosus* are shown in red. The name of the metabolite that was found both in the monoculture of *S. rimosus* and the coculture is shown in violet. The name of the metabolite that was found both in the monoculture of *P. rubens* and the coculture is shown in navy blue. The name of the metabolite that was found solely in the monoculture of *P. rubens* is shown in black. The metabolites were tentatively identified on the basis of M⊚ data, however, their identities were not confirmed due to a lack of analytical standards. The exceptions were the confirmed identities of oxytetracycline and penicillin G.

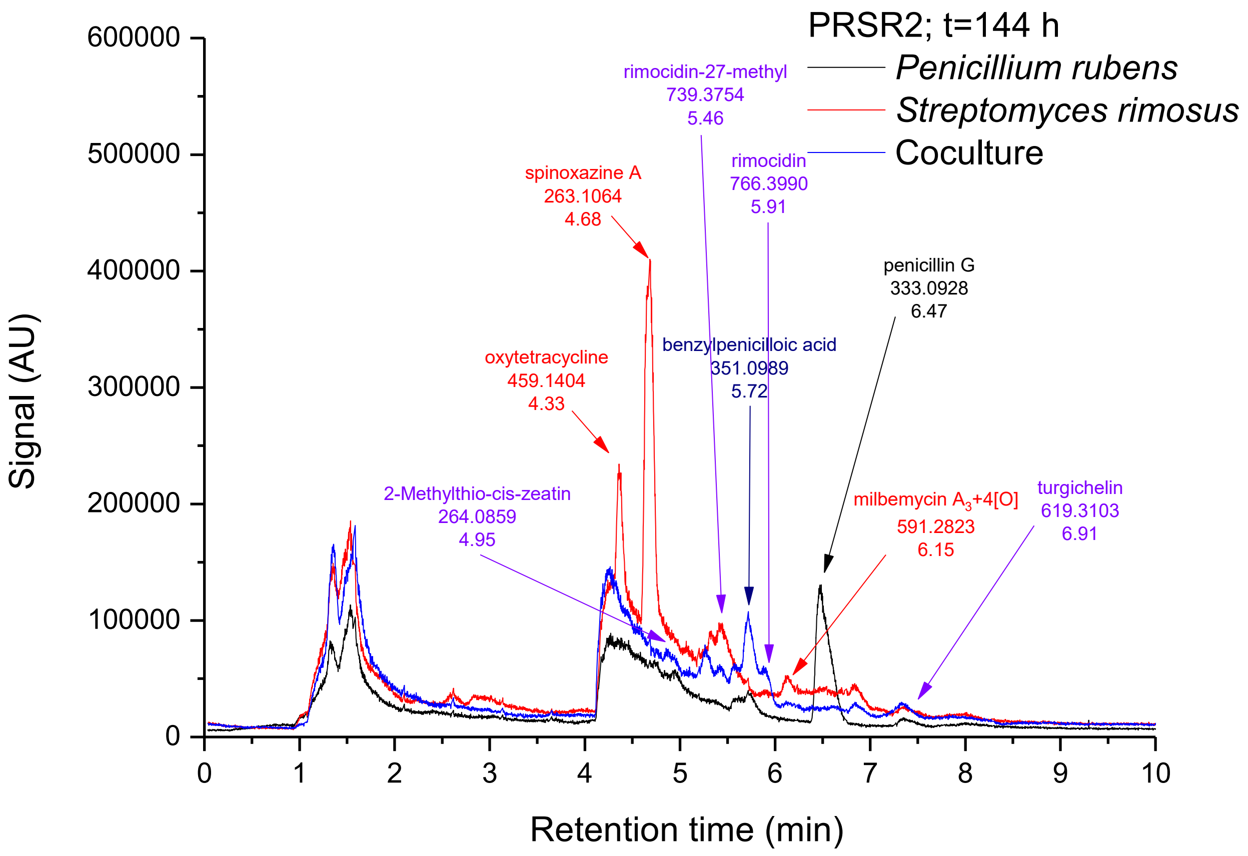

Figure S18 Alignments of total ion chromatograms (TICs) recorded in the PR⊚R2 experiment at t=144 h for *P. rubens* monoculture (black line), *S. rimosus* monoculture (red line), and the "*P. rubens* vs. *S. rimosus*" coculture (blue line). AU-auxiliary units. The names of the metabolites that were found solely in the monoculture of *S. rimosus* are shown in red. The names of the metabolites that were found both in the monoculture of *S. rimosus* and the coculture are shown in violet. The name of the metabolite that was found both in the monoculture of *P. rubens* and the coculture is shown in navy blue. The name of the metabolite that was found solely in the monoculture of *P. rubens* is shown in black. The metabolites were tentatively identified on the basis of M⊚ data, however, their identities were not confirmed due to a lack of analytical standards. The exceptions were the confirmed identities of oxytetracycline and penicillin G.

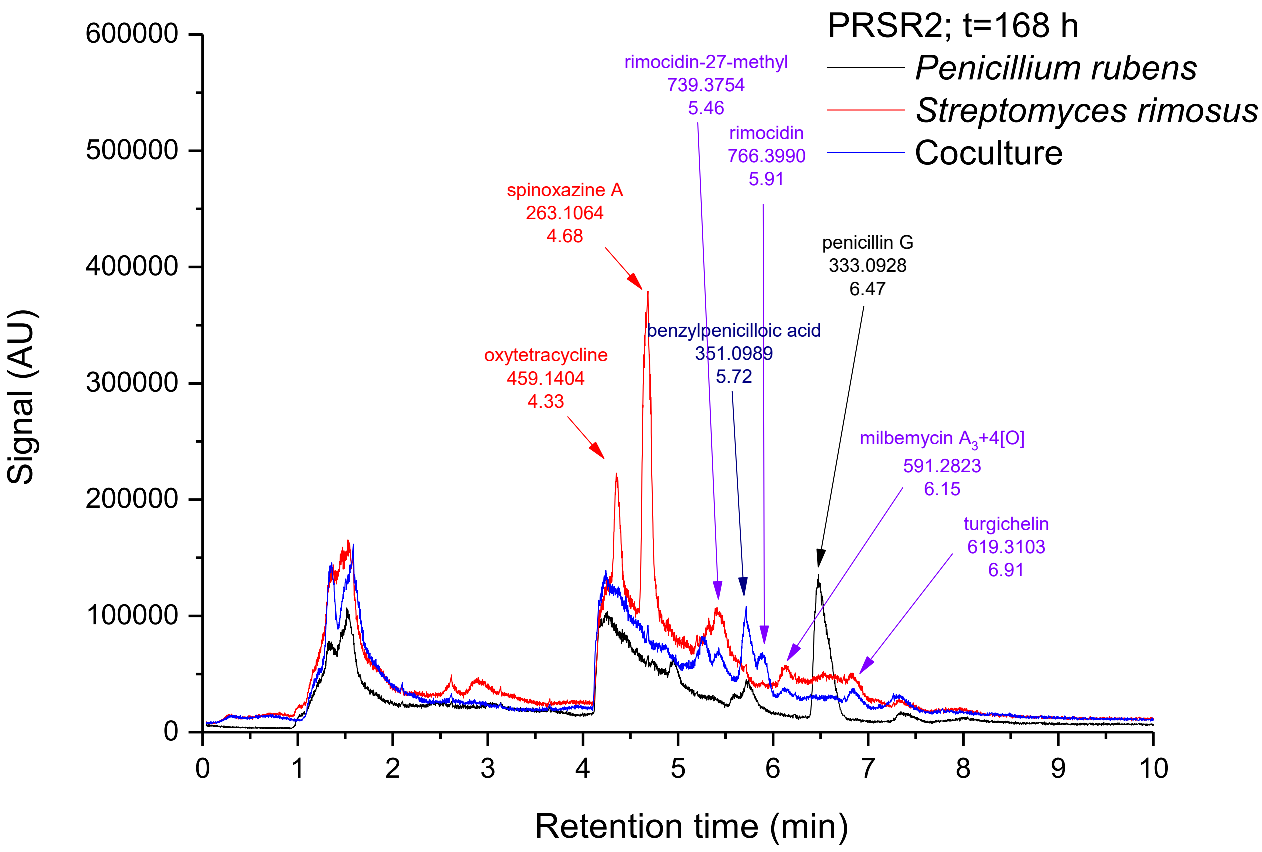

Figure S19 Alignments of total ion chromatograms (TICs) recorded in the PR◎R2 experiment at t=168 h for *P. rubens* monoculture (black line), *S. rimosus* monoculture (red line), and the "*P. rubens* vs. *S. rimosus*" coculture (blue line). AU-auxiliary units. The names of the metabolites that were found solely in the monoculture of *S. rimosus* are shown in red. The names of the metabolites that were found both in the monoculture of *S. rimosus* and the coculture are shown in violet. The name of the metabolite that was found both in the monoculture of *P. rubens* and the coculture is shown in navy blue. The name of the metabolite that was found solely in the monoculture of *P. rubens* is shown in black. The metabolites were tentatively identified on the basis of M◎ data, however, their identities were not confirmed due to a lack of analytical standards. The exceptions were the confirmed identities of oxytetracycline and penicillin G.

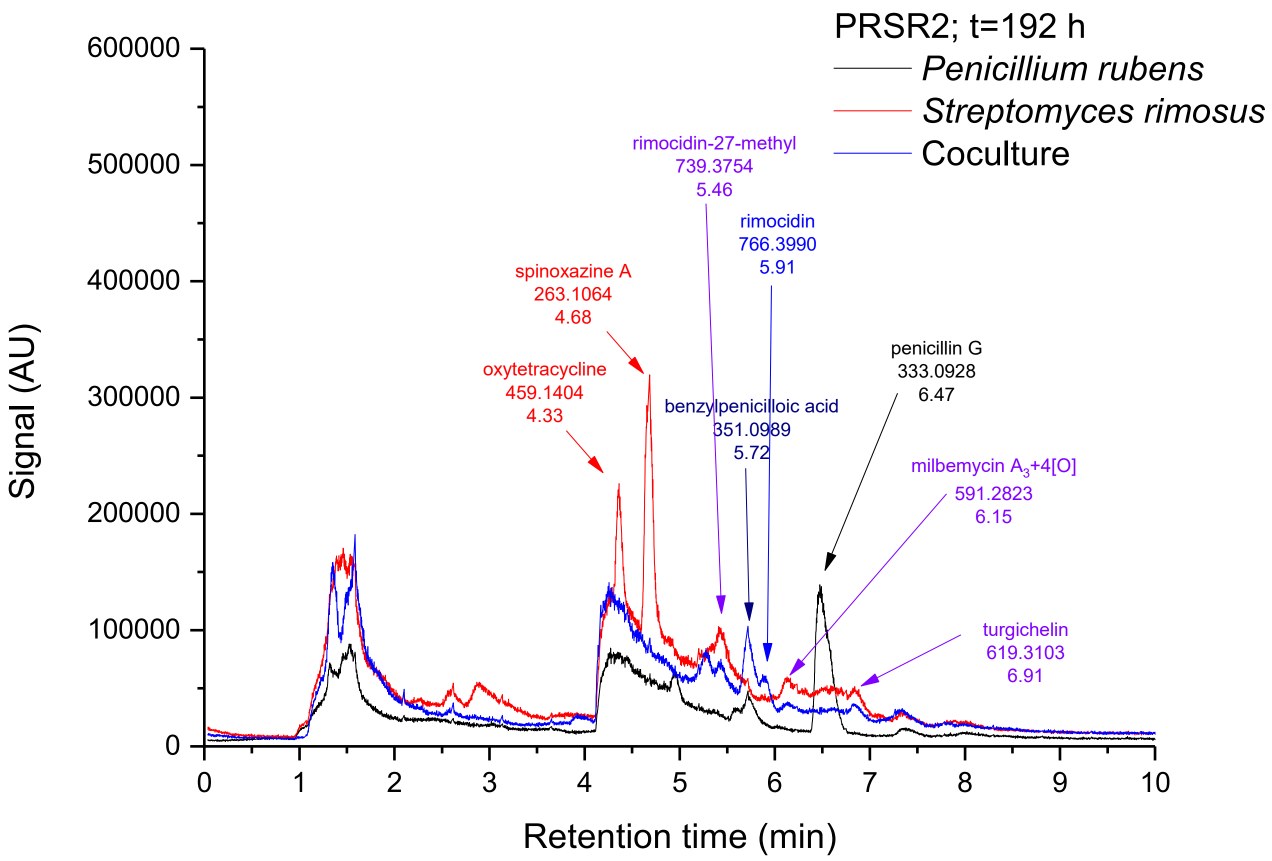

Figure S20 Alignments of total ion chromatograms (TICs) recorded in the PR⊚R2 experiment at t=192 h for *P. rubens* monoculture (black line), *S. rimosus* monoculture (red line), and the "*P. rubens* vs. *S. rimosus*" coculture (blue line). AU-auxiliary units. The names of the metabolites that were found solely in the monoculture of *S. rimosus* are shown in red. The names of the metabolites that were found both in the monoculture of *S. rimosus* and the coculture are shown in violet. The name of the metabolite that was found both in the monoculture of *P. rubens* and the coculture is shown in navy blue. The name of the metabolite that was found solely in the monoculture of *P. rubens* is shown in black. The name of the metabolite that was found solely in the coculture is shown in blue. The metabolites were tentatively identified on the basis of M⊚ data, however, their identities were not confirmed due to a lack of analytical standards. The exceptions were the confirmed identities of oxytetracycline and penicillin G.

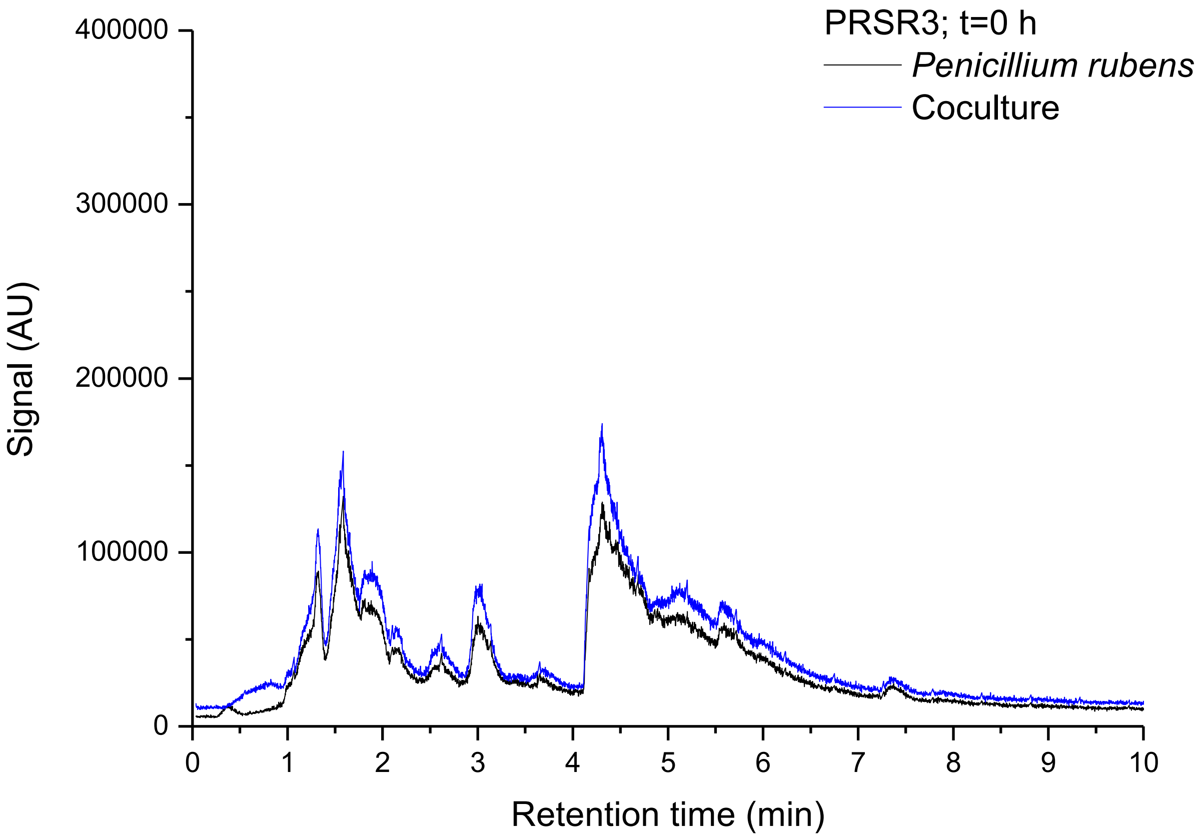

Figure S21 Alignments of total ion chromatograms (TICs) recorded in the PR◎R3 experiment at t=0 h for *P. rubens* monoculture (black line) and the "*P. rubens* vs. *S. rimosus*" coculture (blue line). AU-auxiliary units.

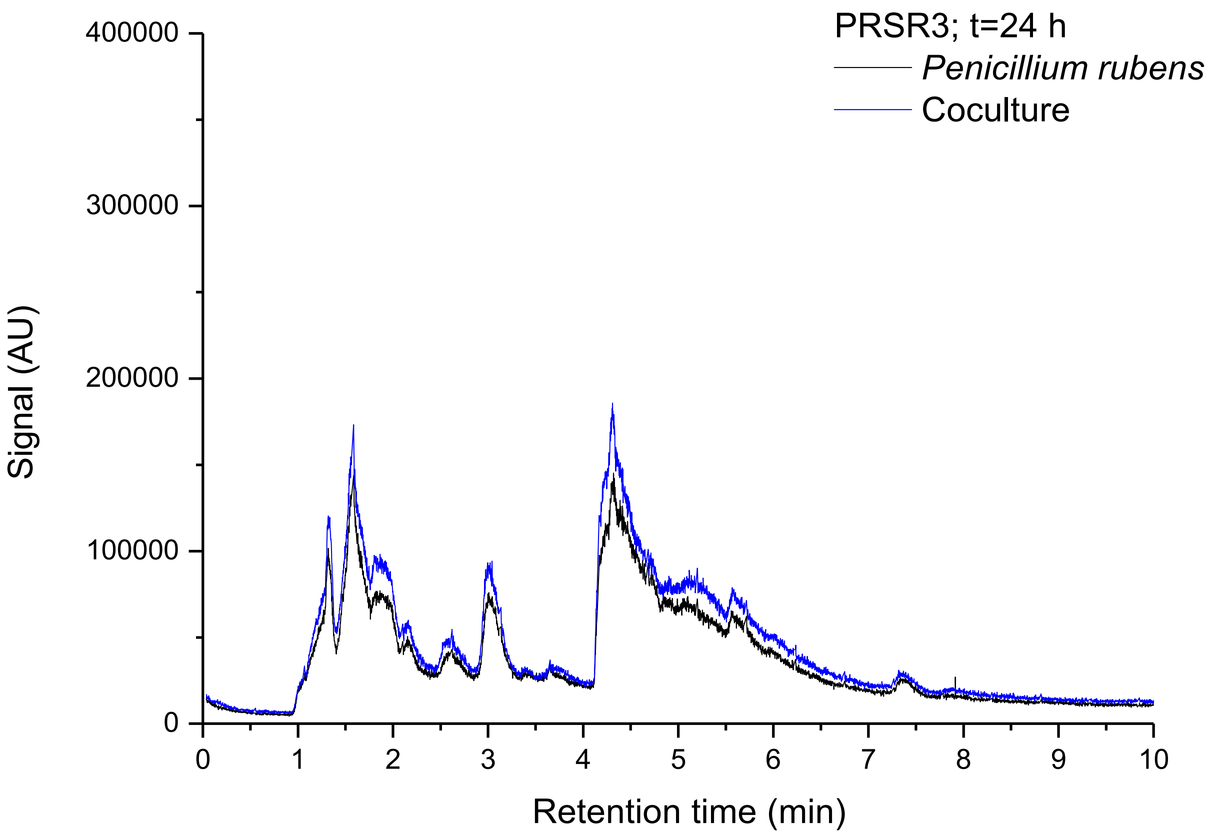

Figure S22 Alignments of total ion chromatograms (TICs) recorded in the PR⊚R3 experiment at t=24 h for *P. rubens* monoculture (black line) and the "*P. rubens* vs. *S. rimosus*" coculture (blue line). AU-auxiliary units.

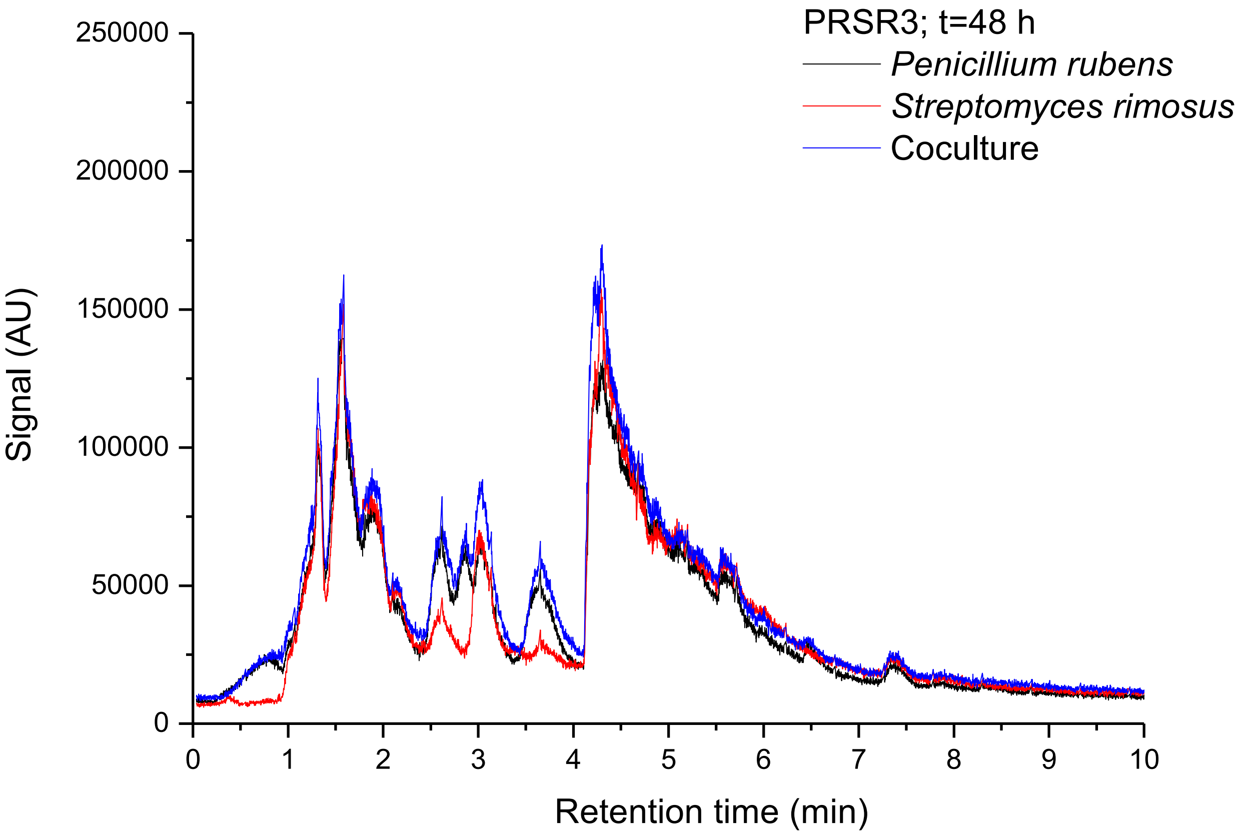

Figure S23 Alignments of total ion chromatograms (TICs) recorded in the PR⊚R3 experiment at t=48 h for *P. rubens* monoculture (black line), *S. rimosus* monoculture (red line), and the "*P. rubens* vs. *S. rimosus*" coculture (blue line). AU- auxiliary units.

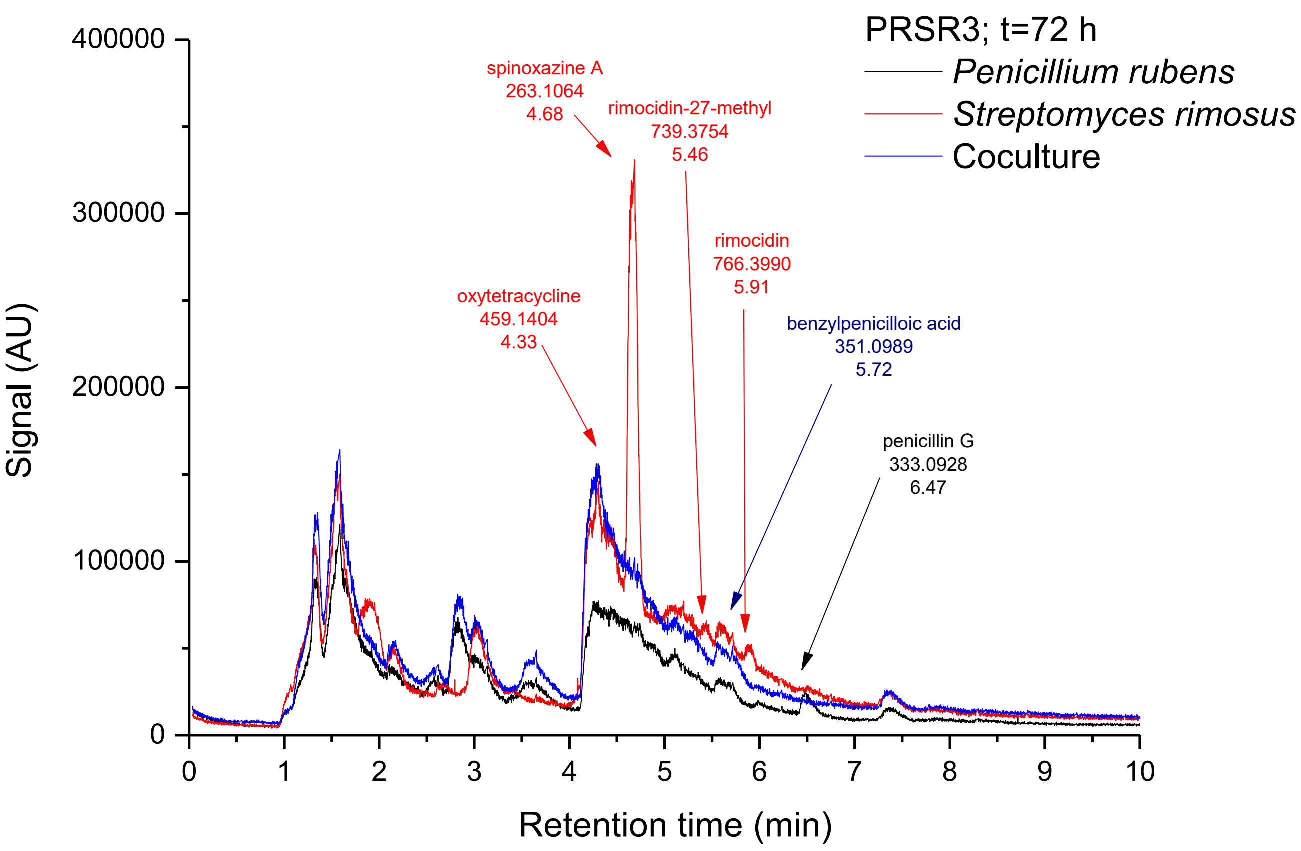

Figure S24 Alignments of total ion chromatograms (TICs) recorded in the PR⊚R3 experiment at t=72 h for *P. rubens* monoculture (black line), *S. rimosus* monoculture (red line), and the "*P. rubens* vs. *S. rimosus*" coculture (blue line). AU-auxiliary units. The names of the metabolites that were found solely in the monoculture of *S. rimosus* are shown in red. The name of the metabolite that was found both in the monoculture of *P. rubens* and the coculture is shown in navy blue. The name of the metabolite that was found solely in the monoculture of *P. rubens* is shown in black. The metabolites were tentatively identified on the basis of M⊚ data, however, their identities were not confirmed due to a lack of analytical standards. The exceptions were the confirmed identities of oxytetracycline and penicillin G.

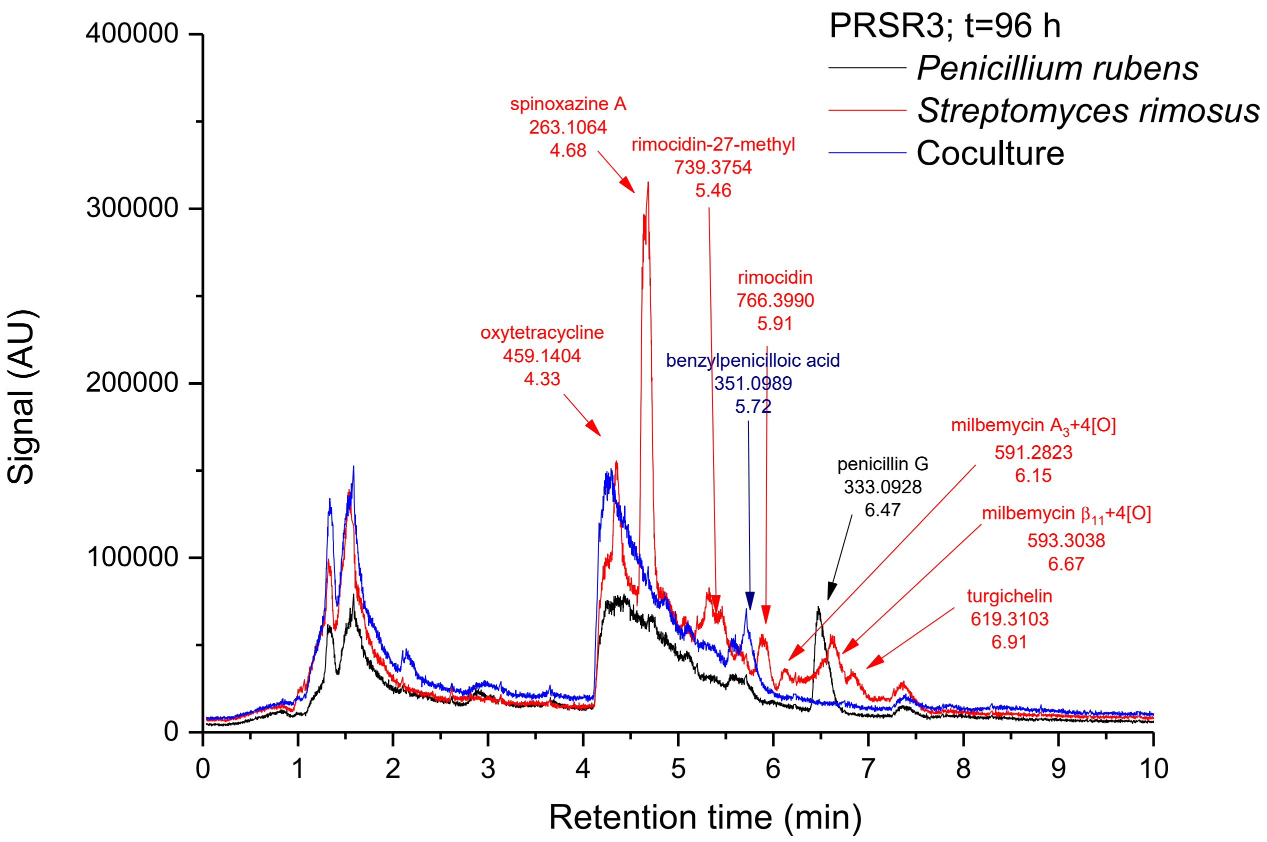

Figure S25 Alignments of total ion chromatograms (TICs) recorded in the PR◎R3 experiment at t=96 h for *P. rubens* monoculture (black line), *S. rimosus* monoculture (red line), and the "*P. rubens* vs. *S. rimosus*" coculture (blue line). AU-auxiliary units. The names of the metabolites that were found solely in the monoculture of *S. rimosus* are shown in red. The name of the metabolite that was found both in the monoculture of *P. rubens* and the coculture is shown in navy blue. The name of the metabolite that was found solely in the monoculture of *P. rubens* is shown in black. The metabolites were tentatively identified on the basis of M◎ data, however, their identities were not confirmed due to a lack of analytical standards. The exceptions were the confirmed identities of oxytetracycline and penicillin G.

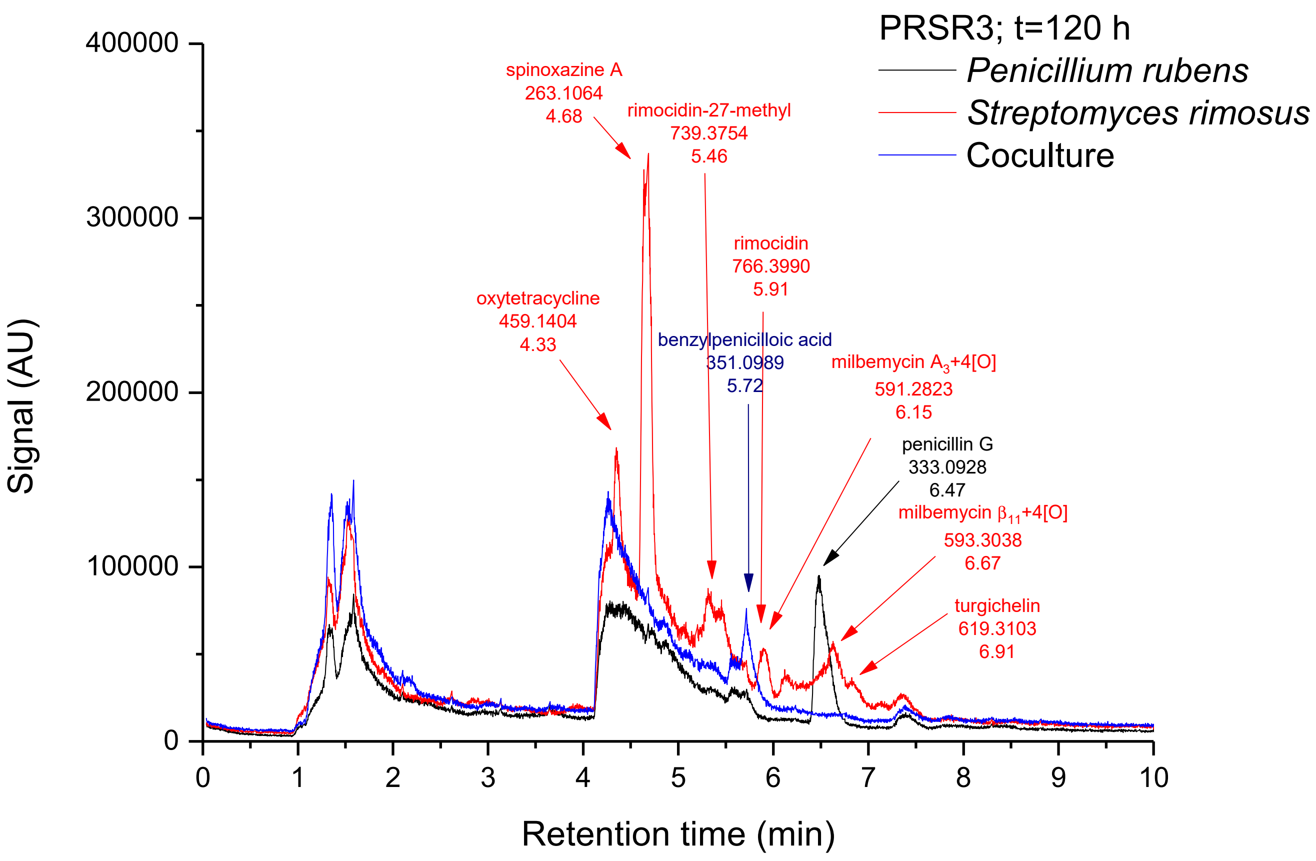

Figure S26 Alignments of total ion chromatograms (TICs) recorded in the PR◎R3 experiment at t=120 h for *P. rubens* monoculture (black line), *S. rimosus* monoculture (red line), and the "*P. rubens* vs. *S. rimosus*" coculture (blue line). AU-auxiliary units. The names of the metabolites that were found solely in the monoculture of *S. rimosus* are shown in red. The name of the metabolite that was found both in the monoculture of *P. rubens* and the coculture is shown in navy blue. The name of the metabolite that was found solely in the monoculture of *P. rubens* is shown in black. The metabolites were tentatively identified on the basis of M◎ data, however, their identities were not confirmed due to a lack of analytical standards. The exceptions were the confirmed identities of oxytetracycline and penicillin G.

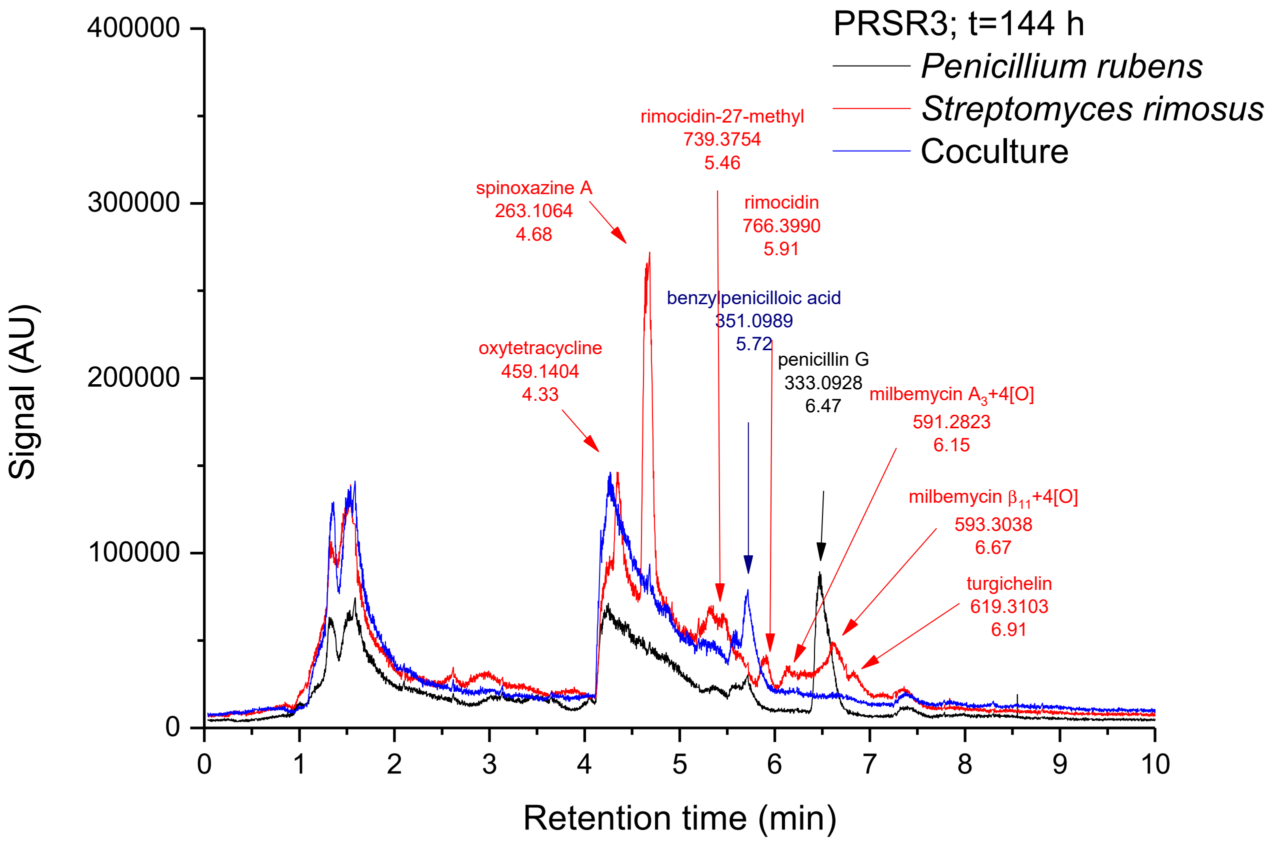

Figure S27 Alignments of total ion chromatograms (TICs) recorded in the PR⊚R3 experiment at t=144 h for *P. rubens* monoculture (black line), *S. rimosus* monoculture (red line), and the "*P. rubens* vs. *S. rimosus*" coculture (blue line). AU-auxiliary units. The names of the metabolites that were found solely in the monoculture of *S. rimosus* are shown in red. The name of the metabolite that was found both in the monoculture of *P. rubens* and the coculture is shown in navy blue. The name of the metabolite that was found solely in the monoculture of *P. rubens* is shown in black. The metabolites were tentatively identified on the basis of M⊚ data, however, their identities were not confirmed due to a lack of analytical standards. The exceptions were the confirmed identities of oxytetracycline and penicillin G.

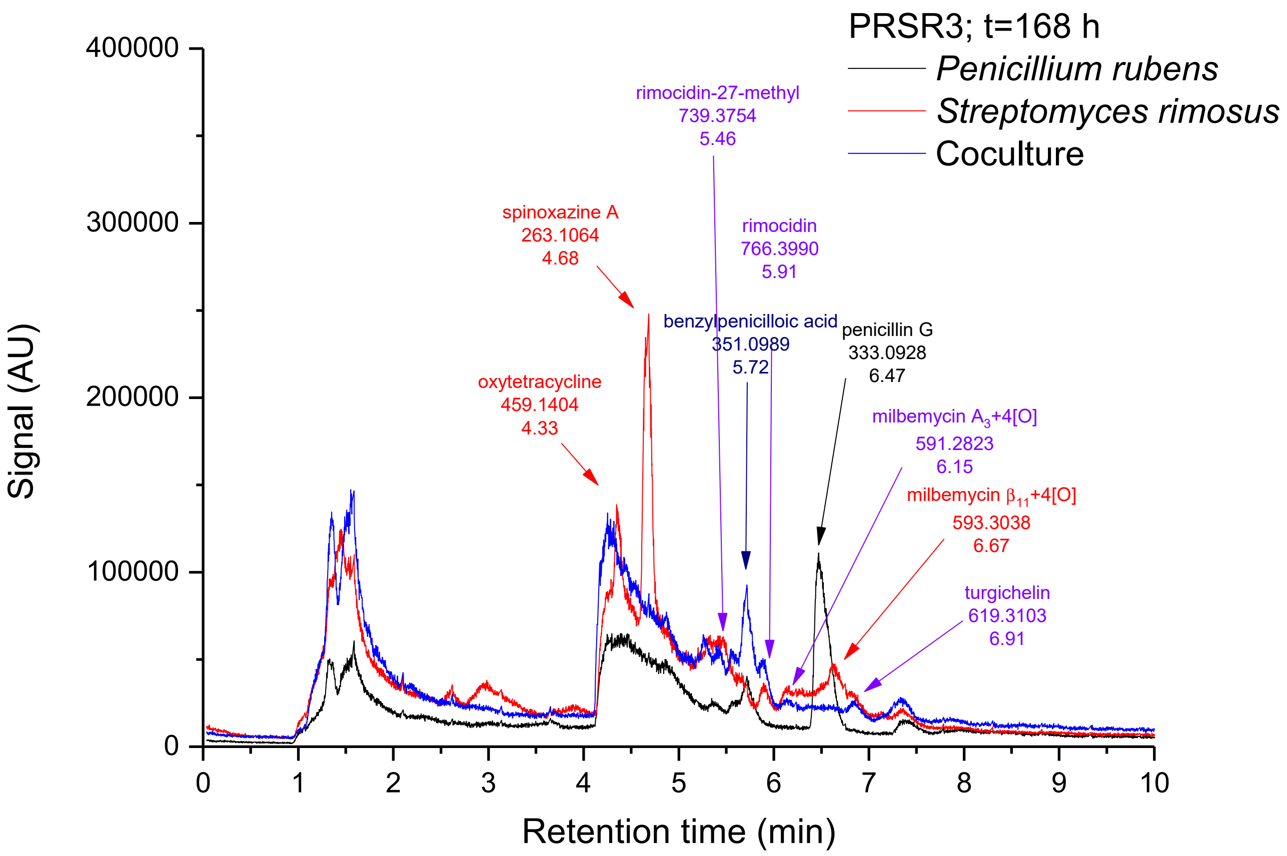

Figure S28 Alignments of total ion chromatograms (TICs) recorded in the PR◎R3 experiment at t=168 h for *P. rubens* monoculture (black line), *S. rimosus* monoculture (red line), and the "*P. rubens* vs. *S. rimosus*" coculture (blue line). AU-auxiliary units. The names of the metabolites that were found solely in the monoculture of *S. rimosus* are shown in red. The names of the metabolites that were found both in the monoculture of *S. rimosus* and the coculture are shown in violet. The name of the metabolite that was found both in the monoculture of *P. rubens* and the coculture is shown in navy blue. The name of the metabolite that was found solely in the monoculture of *P. rubens* is shown in black. The metabolites were tentatively identified on the basis of M◎ data, however, their identities were not confirmed due to a lack of analytical standards. The exceptions were the confirmed identities of oxytetracycline and penicillin G.

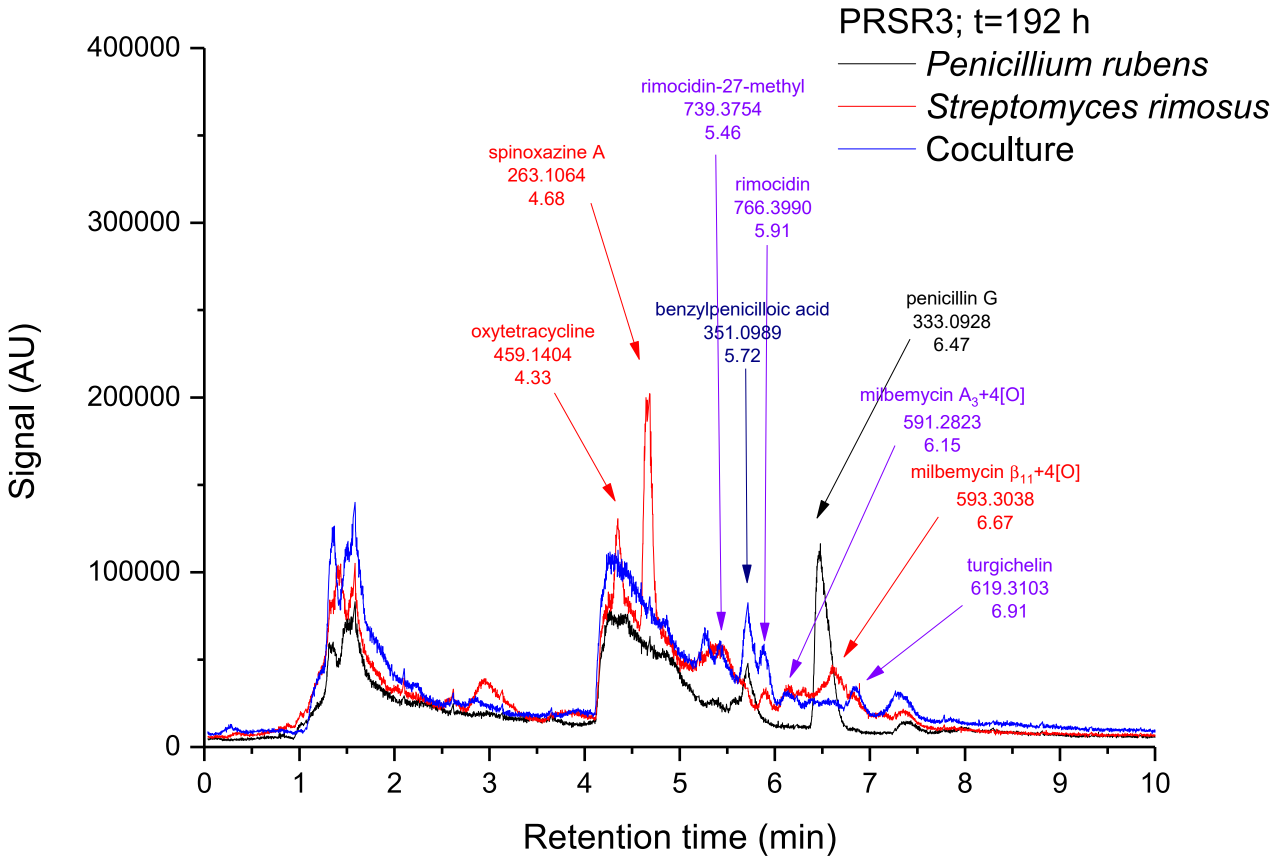

Figure S29 Alignments of total ion chromatograms (TICs) recorded in the PR◎R3 experiment at t=192 h for *P. rubens* monoculture (black line), *S. rimosus* monoculture (red line), and the "*P. rubens* vs. *S. rimosus*" coculture (blue line). AU-auxiliary units. The names of the metabolites that were found solely in the monoculture of *S. rimosus* are shown in red. The names of the metabolites that were found both in the monoculture of *S. rimosus* and the coculture are shown in violet. The name of the metabolite that was found both in the monoculture of *P. rubens* and the coculture is shown in navy blue. The name of the metabolite that was found solely in the monoculture of *P. rubens* is shown in black. The metabolites were tentatively identified on the basis of M◎ data, however, their identities were not confirmed due to a lack of analytical standards. The exceptions were the confirmed identities of oxytetracycline and penicillin G.

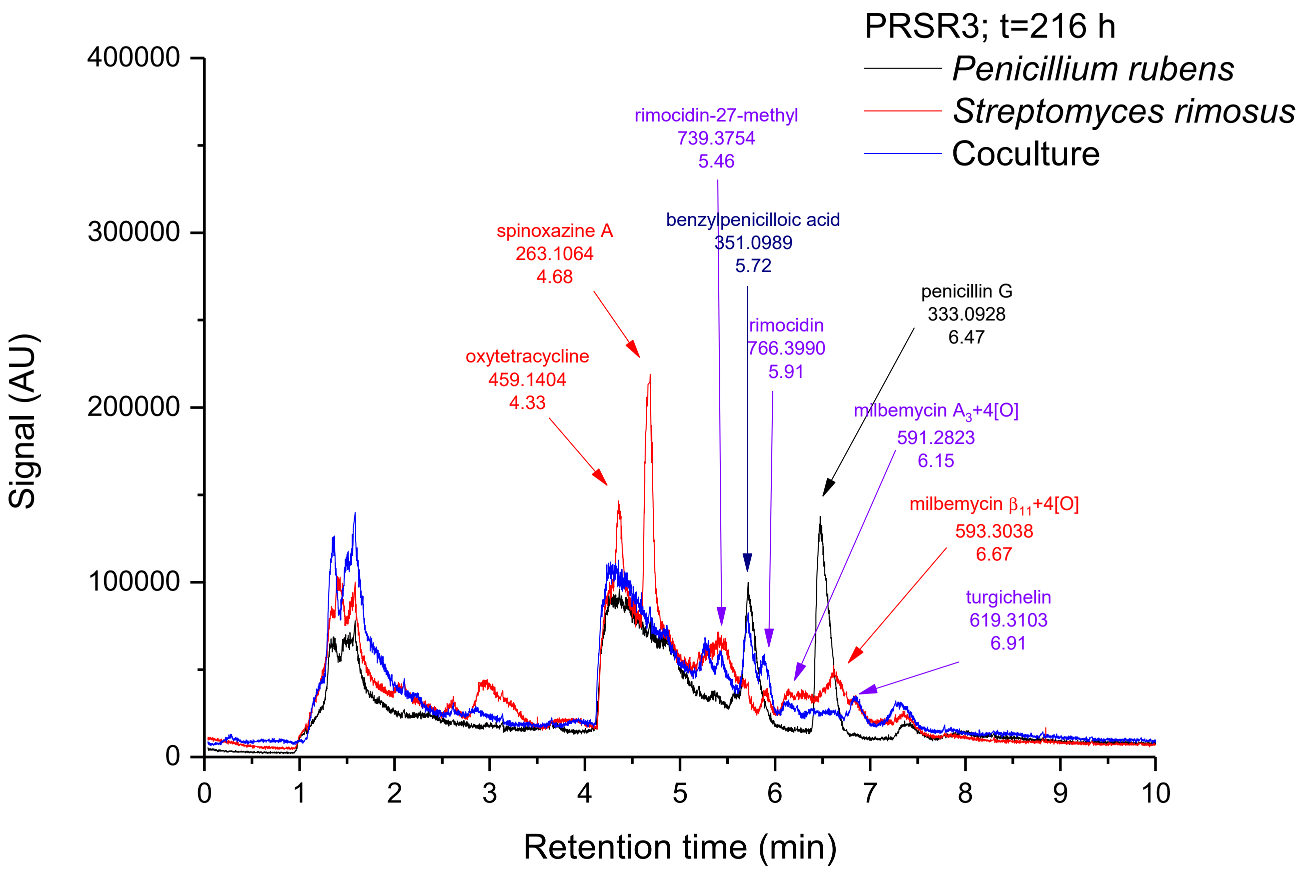

Figure S30 Alignments of total ion chromatograms (TICs) recorded in the PR◎R3 experiment at t=216 h for *P. rubens* monoculture (black line), *S. rimosus* monoculture (red line), and the "*P. rubens* vs. *S. rimosus*" coculture (blue line). AU- auxiliary units. The names of the metabolites that were found solely in the monoculture of *S. rimosus* are shown in red. The names of the metabolites that were found both in the monoculture of *S. rimosus* and the coculture are shown in violet. The name of the metabolite that was found both in the monoculture of *P. rubens* and the coculture is shown in navy blue. The name of the metabolite that was found solely in the monoculture of *P. rubens* is shown in black. The metabolites were tentatively identified on the basis of M◎ data, however, their identities were not confirmed due to a lack of analytical standards. The exceptions were the confirmed identities of oxytetracycline and penicillin G.

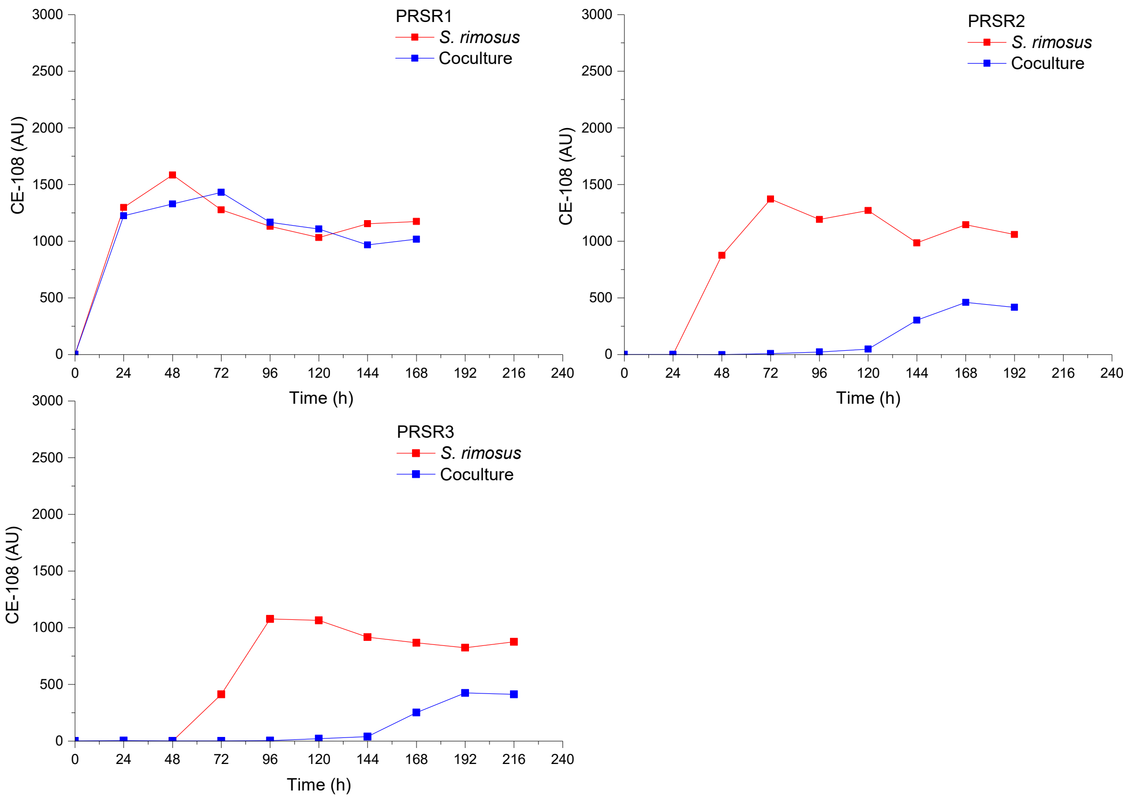

Figure S31 PR◎R1, PR◎R2 and PR◎R3 profiles of CE-108 levels in the "*S. rimosus* vs. *P. rubens*" cocultures and the corresponding monocultures of *S. rimosus*. AU-auxiliary units. The metabolite was tentatively identified on the basis of M◎ data, however, its identity was not confirmed due to a lack of analytical standard.

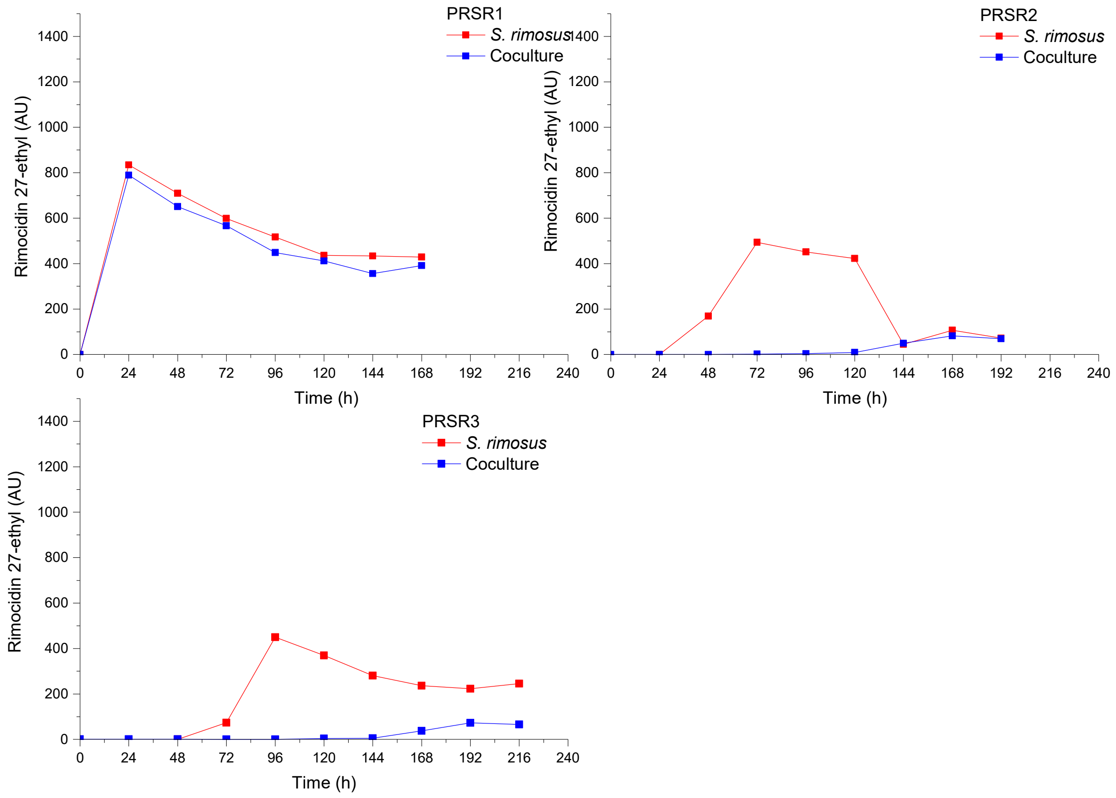

Figure S32 PR◎R1, PR◎R2 and PR◎R3 profiles of rimocidin (27-ethyl) levels in the "*S. rimosus* vs. *P. rubens*" cocultures and the corresponding monocultures of *S. rimosus*. AU-auxiliary units. The metabolite was tentatively identified on the basis of M◎ data, however, its identity was not confirmed due to a lack of analytical standard.

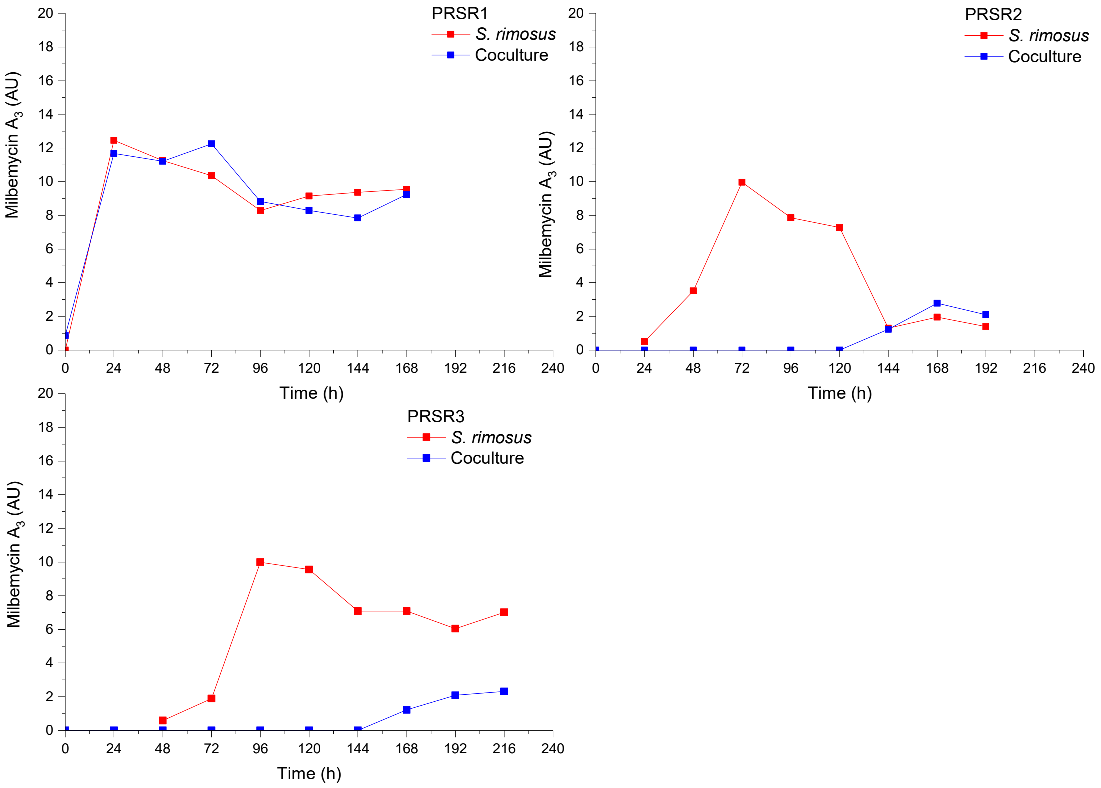

Figure S33 PR◎R1, PR◎R2 and PR◎R3 profiles of milbemycin A_3 levels in the "*S. rimosus* vs. *P. rubens*" cocultures and the corresponding monocultures of *S. rimosus*. AU-auxiliary units. The metabolite was tentatively identified on the basis of M◎ data, however, its identity was not confirmed due to a lack of analytical standard.

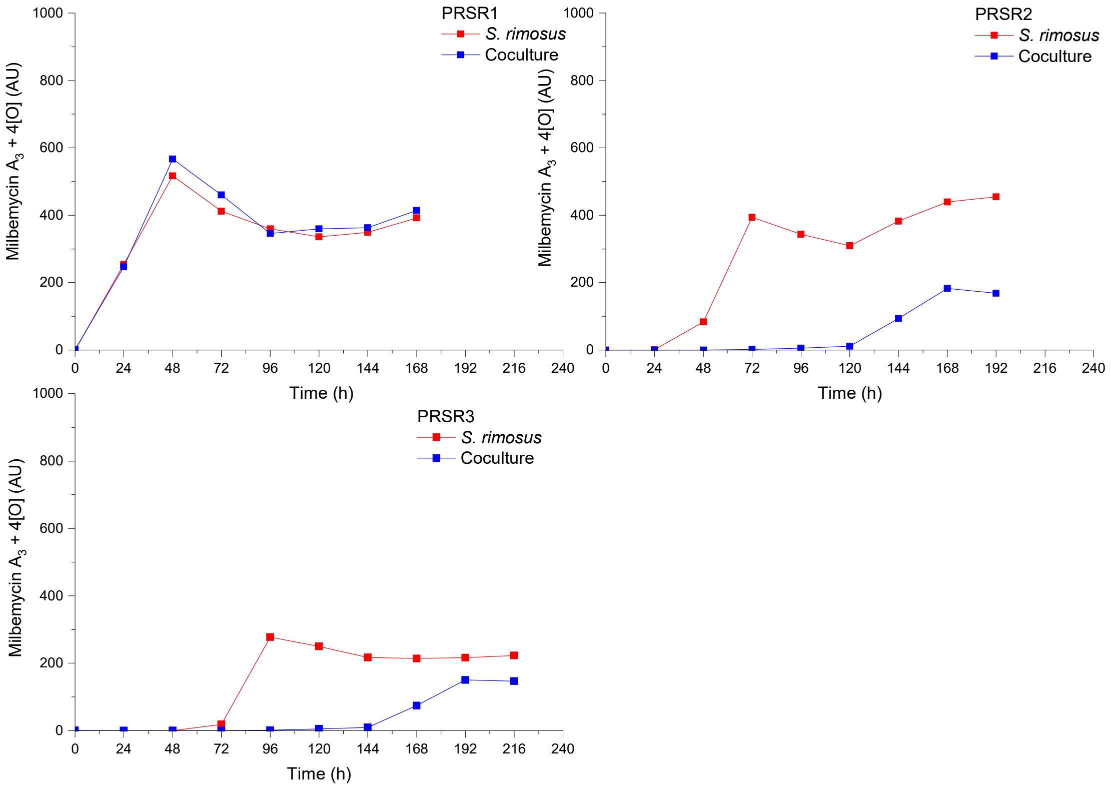

Figure S34 PR◎R1, PR◎R2 and PR◎R3 profiles of milbemycin A3+[4O] levels in the "*S. rimosus* vs. *P. rubens*" cocultures and the corresponding monocultures of *S. rimosus*. AU-auxiliary units. The metabolite was tentatively identified on the basis of M◎ data, however, its identity was not confirmed due to a lack of analytical standard.

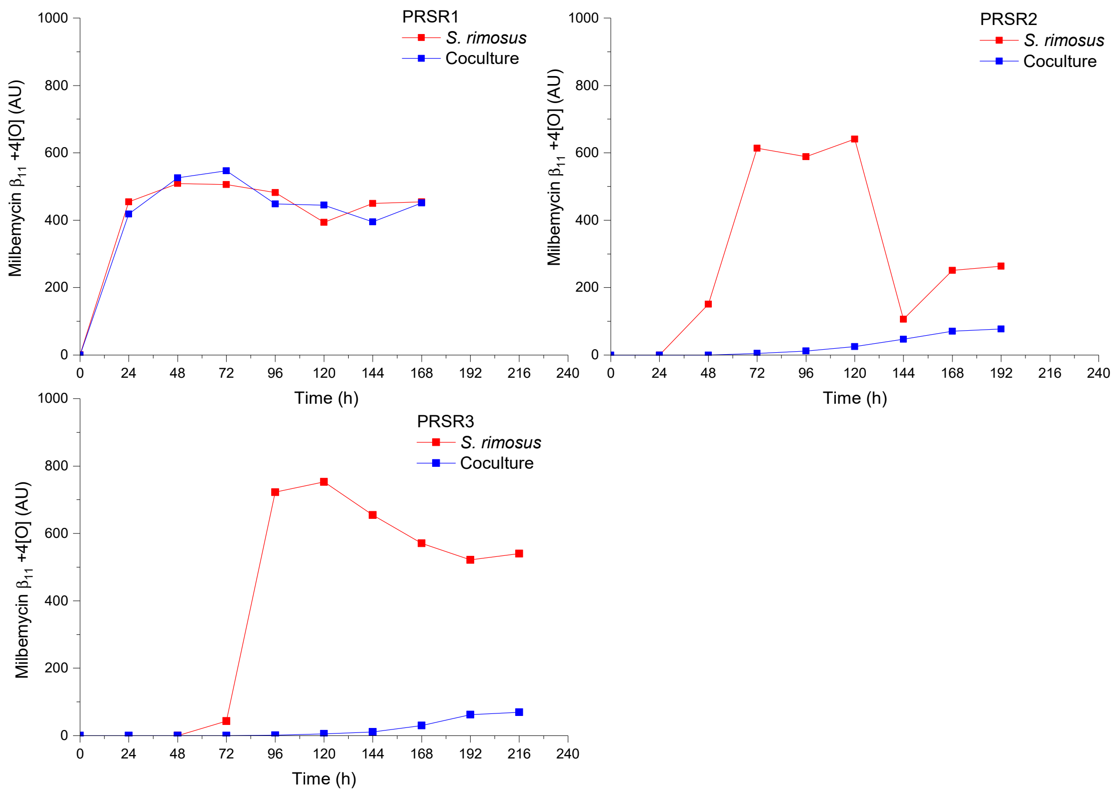

Figure S35 PR◎R1, PR◎R2 and PR◎R3 profiles of milbemycin 11+4[O] levels in the "*S. rimosus* vs. *P. rubens*" cocultures and the corresponding monocultures of *S. rimosus*. AU-auxiliary units. The metabolite was tentatively identified on the basis of M◎ data, however, its identity was not confirmed due to a lack of analytical standard.

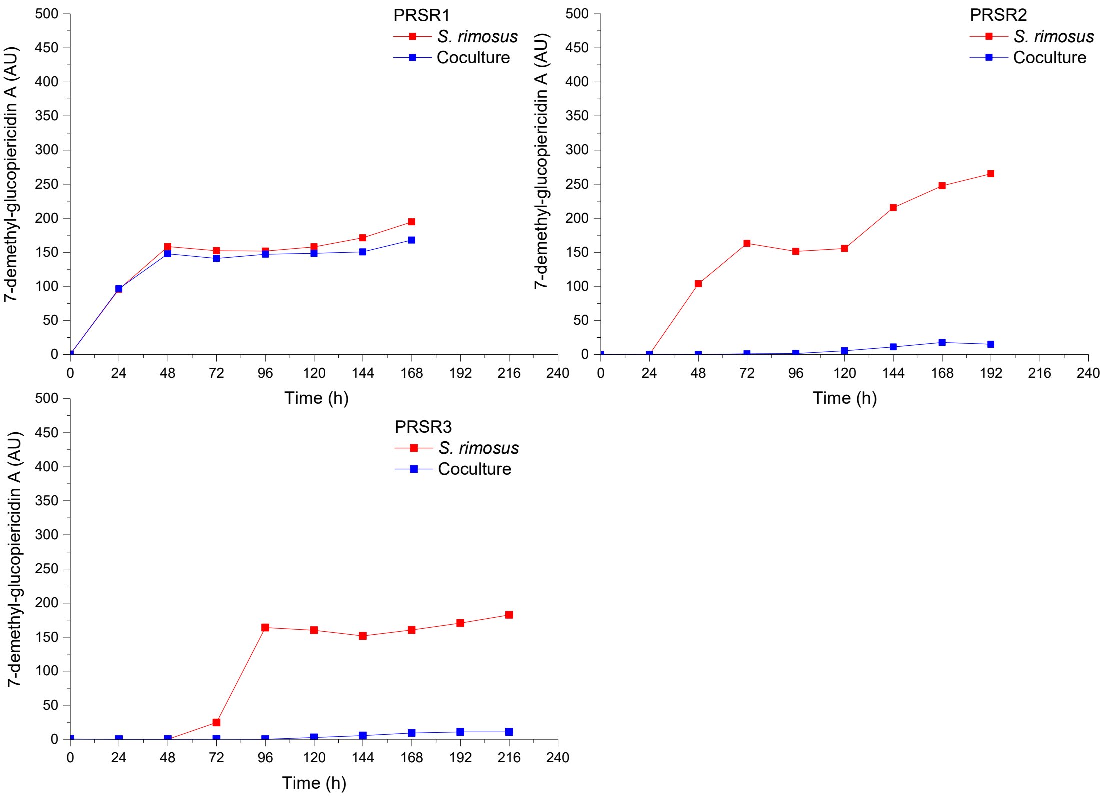

Figure S36 PR◎R1, PR◎R2 and PR◎R3 profiles of 7-demethyl-glucopiericidin A levels in the "*S. rimosus* vs. *P. rubens*" cocultures and the corresponding monocultures of *S. rimosus*. AU-auxiliary units. The metabolite was tentatively identified on the basis of M◎ data, however, its identity was not confirmed due to a lack of analytical standard.

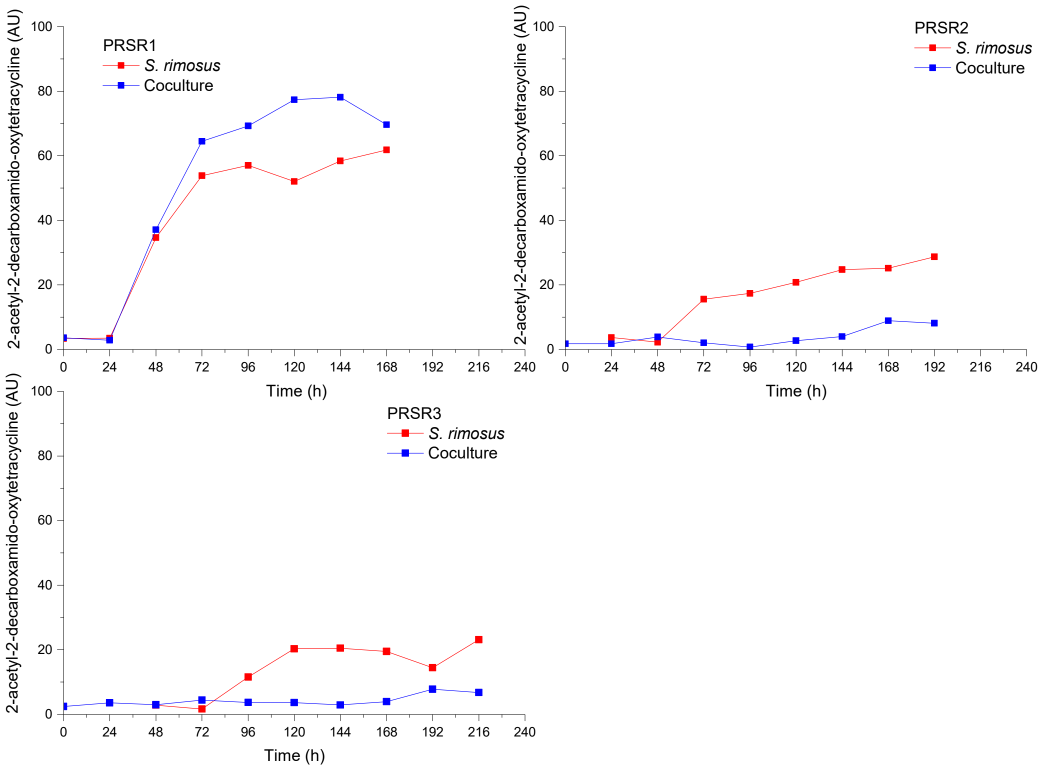

Figure S37 PR⊚R1, PR⊚R2 and PR⊚R3 profiles of 2-acetyl-2-decarboxamido-oxytetracycline (ADOTC) levels in the "*S. rimosus* vs. *P. rubens*" cocultures and the corresponding monocultures of *S. rimosus*. AU-auxiliary units. The metabolite was tentatively identified on the basis of M⊚ data, however, its identity was not confirmed due to a lack of analytical standard.

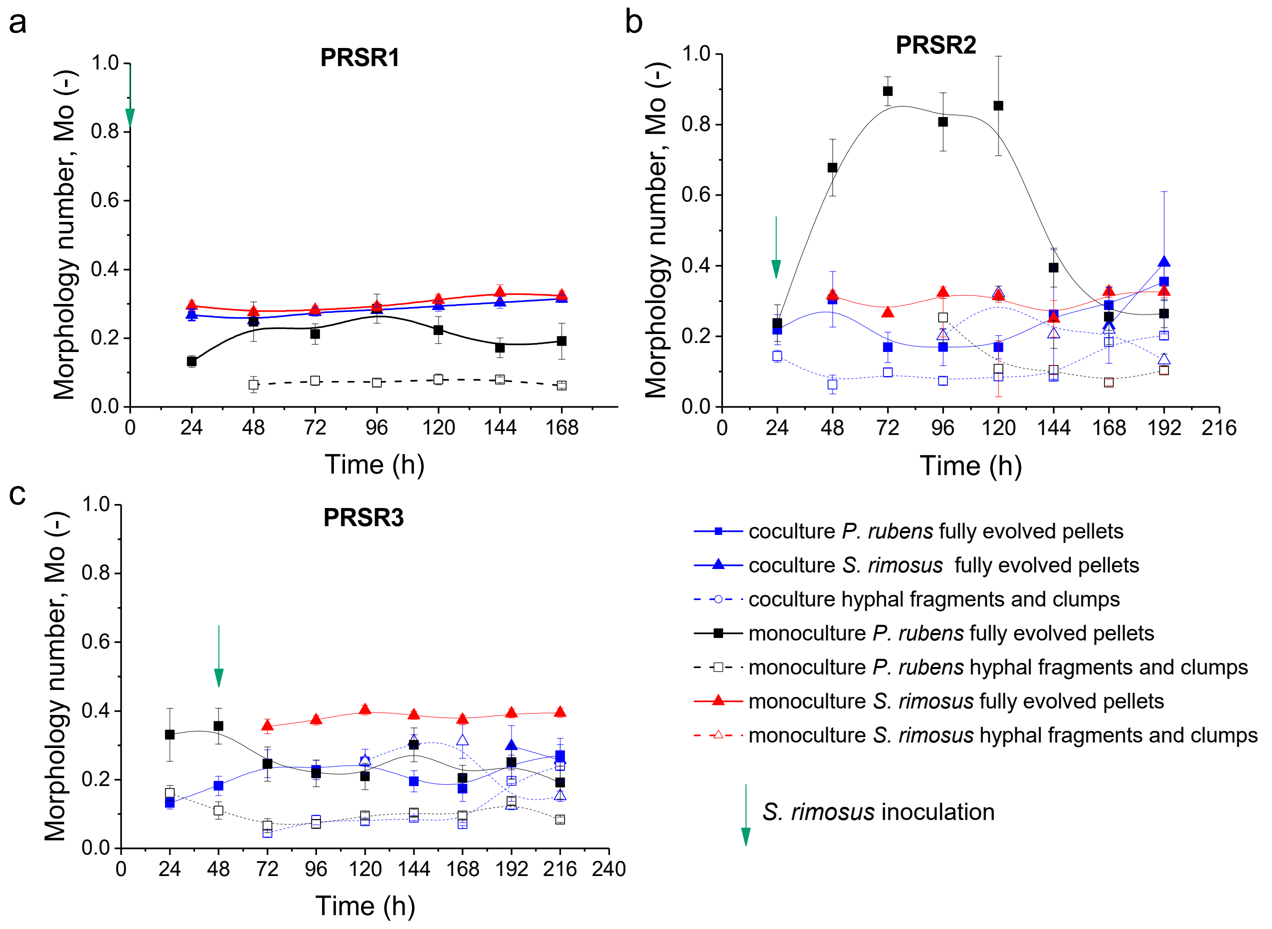

Figure S38 The values of morphology number (Mo) recorded over the course of PR⊚R1 (a), PR⊚R2 (b) and PR⊚R3 (c) experiments for the cocultures, monocultures of *P. rubens* and monocultures of *S. rimosus*.

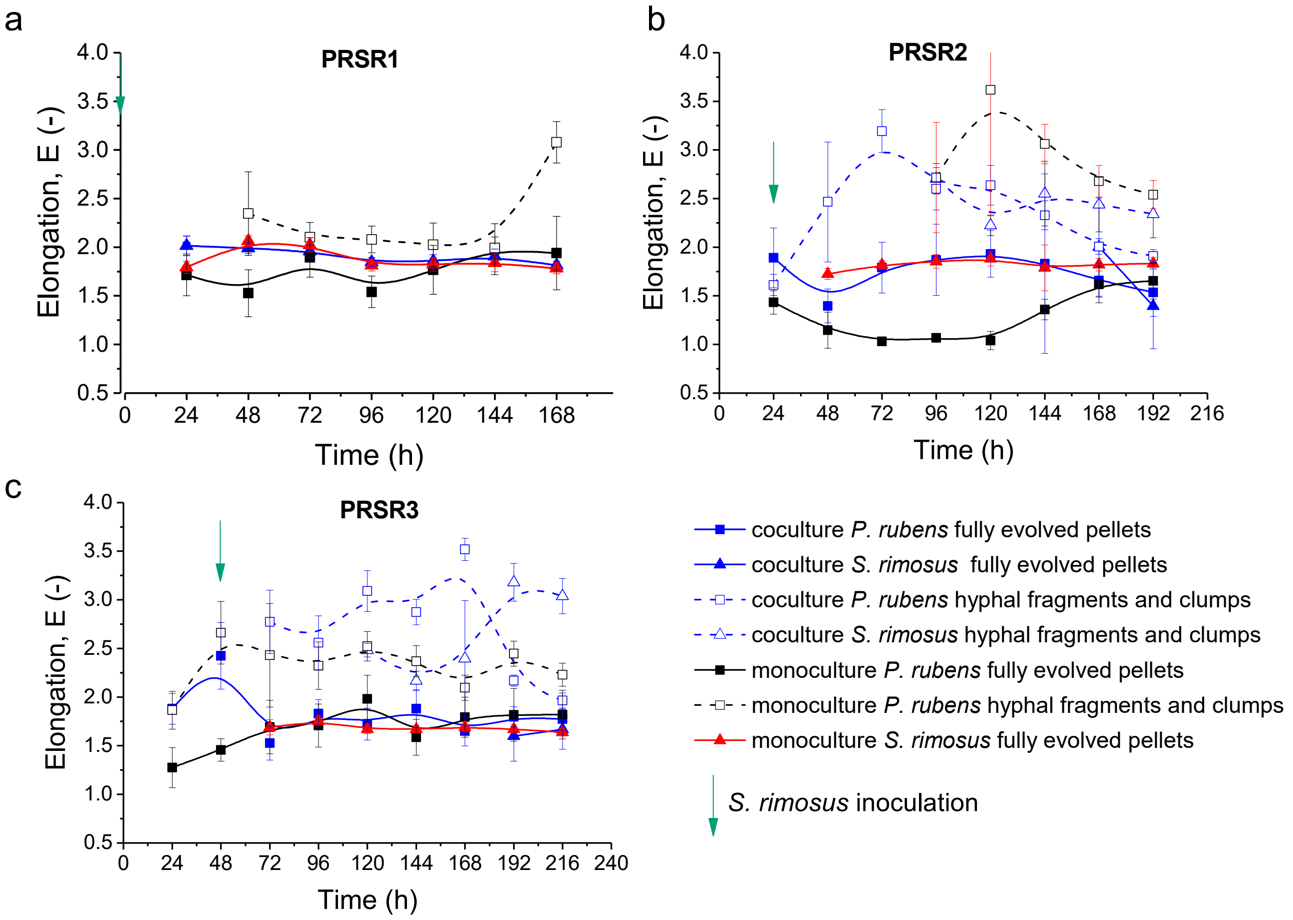

Figure S39 The values of elongation (E) recorded over the course of PR⊚R1 (a), PR⊚R2 (b) and PR⊚R3 (c) experiments for the cocultures, monocultures of *P. rubens* and monocultures of *S. rimosus*.

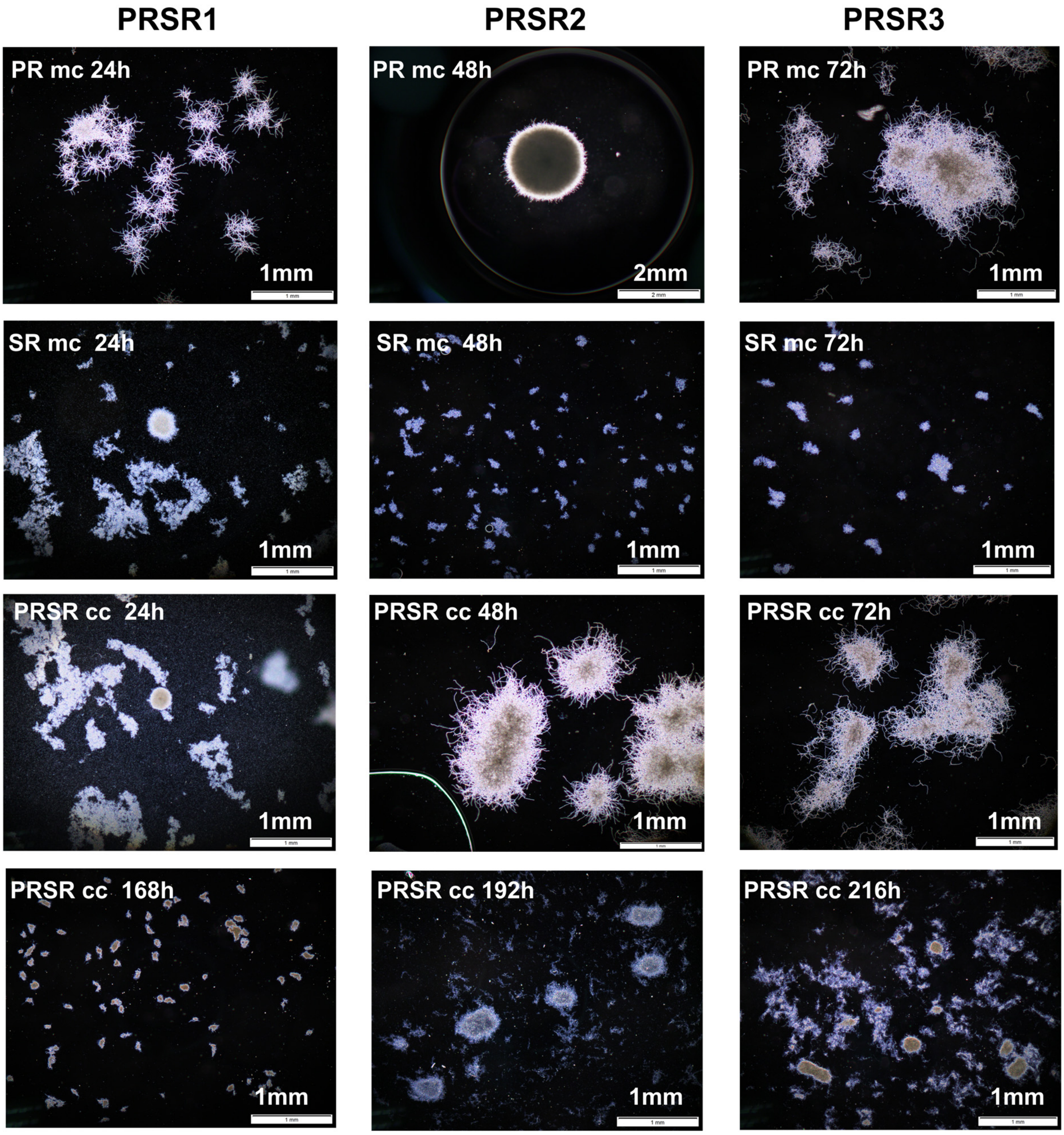

Figure S40 ◎elected microscopic images corresponding to the PR◎R1, PR◎R2, and PR◎R3 experiments. The samples were drawn at the indicated time points. PR – *P. rubens*; ◎R mc– monoculture of *S. rimosus*; PR mc – monoculture of *P. rubens*; PR◎R cc – coculture of *P. rubens* and *S. rimosus*.
